# Self-single-atomization of active sites in electrochemical ammonia synthesis unveiled by machine learning potential

**DOI:** 10.1093/nsr/nwag417

**Published:** 2026-07-09

**Authors:** Jun Long, Chenyu Yang, Huan Li, Hao Li, Jianping Xiao

**Affiliations:** State Key Laboratory of Catalysis, Dalian National Laboratory for Clean Energy, Dalian Institute of Chemical Physics, Chinese Academy of Sciences, Dalian 116023, China; State Key Laboratory of Catalysis, Dalian National Laboratory for Clean Energy, Dalian Institute of Chemical Physics, Chinese Academy of Sciences, Dalian 116023, China; University of Chinese Academy of Sciences, Beijing 100049, China; State Key Laboratory of Catalysis, Dalian National Laboratory for Clean Energy, Dalian Institute of Chemical Physics, Chinese Academy of Sciences, Dalian 116023, China; State Key Laboratory of Catalysis, Dalian National Laboratory for Clean Energy, Dalian Institute of Chemical Physics, Chinese Academy of Sciences, Dalian 116023, China; State Key Laboratory of Catalysis, Dalian National Laboratory for Clean Energy, Dalian Institute of Chemical Physics, Chinese Academy of Sciences, Dalian 116023, China; University of Chinese Academy of Sciences, Beijing 100049, China

**Keywords:** self-single-atomization, electrochemical ammonia synthesis, density functional theory calculation, machine learning potential, Grand Canonical Monte Carlo

## Abstract

Electrochemical nitric oxide (NO) reduction reaction (eNORR) has emerged as a promising route for the synthesis of green ammonia. Fe was observed experimentally with great activity and selectivity for eNORR to ammonia. However, we found that electrochemical performance over iron is not due to its intrinsic activity, but from an adaptive capability of self-single-atomization in operando conditions. We first performed Grand Canonical Monte Carlo simulations driven by a machine learning potential, where we explored ∼2.5 million possible active structures and their evolution with increasing electrode potential. Microkinetic modeling indicates that Fe surface will be predominantly covered by adsorbed N* species in reaction, resulting in the formation of FeN*_x_* motifs by self-single-atomization. Statistical results indicate that the top sites of isolated FeN*_x_* motifs contribute higher activity of ammonia synthesis than bridge and hollow sites. Furthermore, based on the self-single-atomized active sites, we reproduced and rationalized the variation trends of experimental Faradaic efficiency of different products with electrode potential, verifying the reliability of the proposed active sites herein. Hence, we propose to make use of the self-single-atomization capability of catalysts under working conditions to construct active sites beyond ammonia synthesis.

## Teaser

The authors unveiled the in situ generation of single-atom active sites on Fe surface during electrochemical NO reduction through multiscale theoretical simulations framework.

## INTRODUCTION

Ammonia (NH_3_) plays an essential role in the development of modern agriculture and industry. In agriculture, it serves as the primary raw material for nitrogenous fertilizer production. In chemical industry, ammonia is a precursor to produce nitric acid and a high-density chemical energy carrier. Conventionally, ammonia synthesis relies on the Haber–Bosch process, which suffers from high reaction temperature and pressure and massive emission of carbon dioxide. Although the electroreduction of dinitrogen (N_2_) to ammonia (eNRR) can avoid the above problems, the low activity and selectivity of eNRR remain the bottleneck [1].

In recent years, electrochemical nitric oxide (NO) reduction reaction (eNORR) has been proposed as a promising route for sustainable ammonia synthesis [2,3], which often has a much higher Faradaic efficiency (FE) and yield rate of NH_3_ than eNRR. In previous studies, lots of research interest was focused on the electroreduction of high-concentration NO to overcome the limit of mass transfer of NO in aqueous solution. Cu has been identified as the most active and selective transition metal to catalyze electroreduction of high-concentration NO to NH_3_ [3,4], which can achieve a 93.5% FE of NH_3_ at −0.9 V vs. reversible hydrogen electrode (RHE) with 100% NO as feeding gas. However, NO is from exhausted gas or plasma-driven NO*_x_* synthesis [5,6]. As a result, the NO concentration is very low in practice. Hence, a variety of catalysts beyond Cu were developed for NO electroreduction at low concentrations, including metals and alloys [7–13], metal oxides [14–17], phosphides [18,19], sulphides [20,21], and carbon-based materials [22,23]. Among them, Fe exhibits great performance under various low concentrations of NO (1%, 5%, and 10%) owing to the superior yield rate and FE of NH_3_, as shown in Fig. S1a. At 1% NO concentration, the NH_3_ yield rate on Fe can achieve 140.7 μmol cm^−2^ h^−1^ with the FE of 96% at −0.6 V vs. RHE [8]. Furthermore, the selectivity of eNORR on Fe shows an interesting potential dependence. The dominant product turns from N_2_O to N_2_, NH_3_, and H_2_ successively as the potential changes from 0.3 to −0.8 V vs. RHE (Fig. S1b), which is poorly understood.

In this work, we employed the Grand Canonical Monte Carlo (GCMC) simulations driven by a machine learning potential (MLP) to investigate the dynamic structural evolution of Fe in eNORR. We found that the Fe surface would be self-single-atomized by interacting with the adsorbed N* species, resulting in the FeN*_x_* single-atom sites. The FeN*_x_* sites can boost the NH_3_ production activity by weakening the bonding strength with reaction intermediates, explaining well the experiments. Moreover, based on the self-single-atomized active sites, the observed potential-dependent selectivity of eNORR on Fe was also rationalized via microkinetic modeling. This work unveils the adaptive capability of Fe catalyst in working conditions by self-single-atomization to enhance its activity.

## RESULTS AND DISCUSSION

### Poor activity of pristine Fe

**Figure 1.**
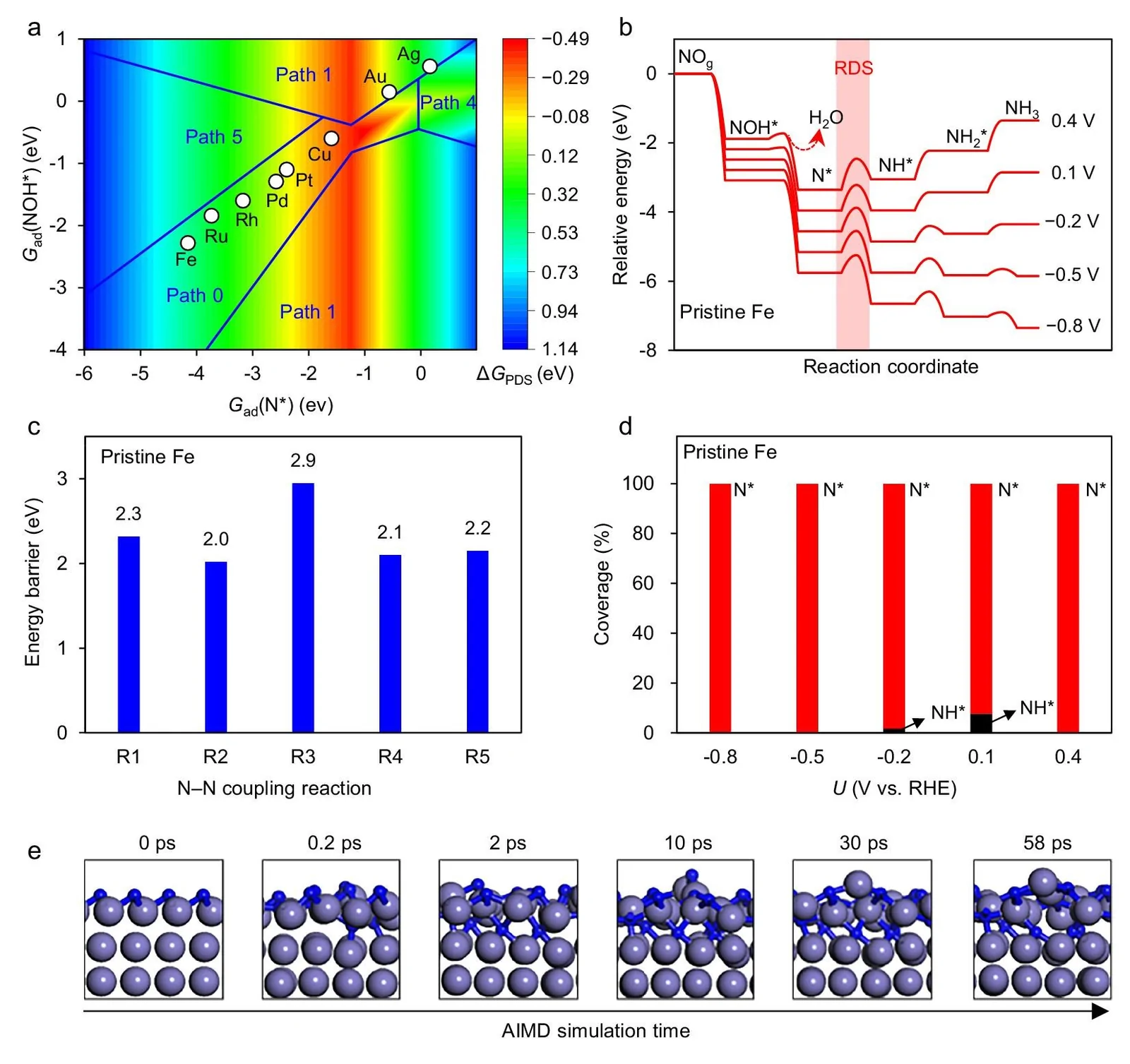
(a) (Quasi) activity and mechanism map of eNORR to NH_3_ on transition metals using *G*_ad_(N*) and *G*_ad_(NOH*) as descriptors. The color represents the reaction free energy of the potential-determining step (Δ*G*_PDS_). The *G*_ad_ of relevant intermediates on Ag, Au, Cu, Pt, Pd, Rh, and Ru are adopted from ref [3]. (b) Free energy diagrams of eNORR to NH_3_ on pristine Fe at varying potential. The ‘RDS’ denotes the rate-determining step. (c) Energy barriers of key N–N coupling reactions for N_2_O and N_2_ production on pristine Fe. R1: 2N*→N_2_*, R2: NO*+NOH*→ N_2_O_2_H*, R3: N*+NO*→N_2_O*, R4: 2NOH*→N_2_O_2_H_2_*, R5: N*+NOH*→N_2_OH*. (d) Coverage of intermediates for eNORR on Fe resulted from microkinetic simulations at varying potential. (e) AIMD simulation for the structural evolution of the Fe surface under the adsorption of a monolayer of N*.

We first investigated the intrinsic activity of pristine Fe using Fe (110) surface as the model by density functional theory (DFT) calculations (Supporting Information S1.1). The catalytic reaction phase diagram (CatRPD) package [24,25] (Supporting Information S1.2) was employed to construct the (quasi) activity and mechanism map of eNORR to NH_3_ based on the scaling relations (Fig. S2) between adsorption free energies (*G*_ad_) of intermediates. As shown in Fig. 1a, the intrinsic activity of pristine Fe for eNORR to NH_3_ is inferior to Cu, Pt, and Ru, against the statistical experimental results in Fig. S1a. According to CatRPD analysis (Fig. 1a), path 0 (see Table S1 for details) is identified as the most thermodynamically favorable reaction pathway for eNORR to NH_3_ on pristine Fe. Moreover, by the method of electric field controlling constant potential (EFC–CP) [26,27] (Supporting Information S1.3), the electrochemical barriers of the elementary steps in path 0 to path 5 were calculated (Figs S3 and S4), based on which the turnover frequencies (TOF) of different pathways were calculated by microkinetic modeling (Supporting Information S1.4). The results indicate that path 0 is also kinetically preferred (Fig. S5). Figure 1b plots the free energy diagrams of eNORR to NH_3_ on pristine Fe at various potentials. At 0.4 V vs. RHE, the formation of NOH* and N* is exothermic, while the successive protonation of N* to NH*, NH_2_*, and NH_3_ is endothermic, with the highest barrier of 0.9 eV for protonation of N*. With the increase of overpotential (more negative potential), the reaction free energies of N*, NH*, and NH_2_* protonation get lower and all of them become exothermic at −0.5 V vs. RHE. Hence, in light of thermodynamics, the reaction can only occur spontaneously when the potential is more negative than −0.5 V vs. RHE on pristine Fe. However, the NH_3_ production on Fe was experimentally observed at −0.2 V vs. RHE [8], which is superior to pristine Fe. Furthermore, within the potential range of −0.1 to 0.3 V vs. RHE, N_2_O and N_2_ are the dominant products in experiments (Fig. S1b), while the energy barriers of the key N–N coupling reactions are above 2 eV on pristine Fe (Figs 1c and S6), which are nearly insurmountable at ambient reaction conditions. Note that herein the Eley–Rideal-like (E–R) mechanism of adsorbed N* coupling with NO gas (NO_g_) was not considered on pristine Fe because the NO adsorption (NO_g_+*→NO*) and protonation-associated adsorption (NO_g_+(H^+^+e^−^)+*→NOH*) are much more feasible than NO_g_ coupling with N* (Fig. S7).

Then, the microkinetic modeling was performed (Supporting Information S1.4, Table S2) for eNORR to NH_3_ on pristine Fe, which indicates that the Fe will be predominantly covered by adsorbed N* with the coverage >90% at different potentials (Fig. 1d). That is because the production of N* from NO is facile, while the conversion of N* to NH* is difficult, as indicated by the highest barrier (Fig. 1b). In this case, the adsorbed N* species will be accumulated on the Fe surface in the practical eNORR process. An *ab initio* molecular dynamics (AIMD) simulation (Supporting Information S1.5) was performed to detect the structural evolution of Fe under the adsorption of a monolayer N* at ambient temperature (298 K), which indicates that the N* can diffuse to the subsurface (Fig. 1e). As N* is continuously produced from NO reduction, and the active surface structures under reaction conditions are still unrevealed without statistical analysis.

### Structural reconstruction of Fe in eNORR

**Figure 2.**
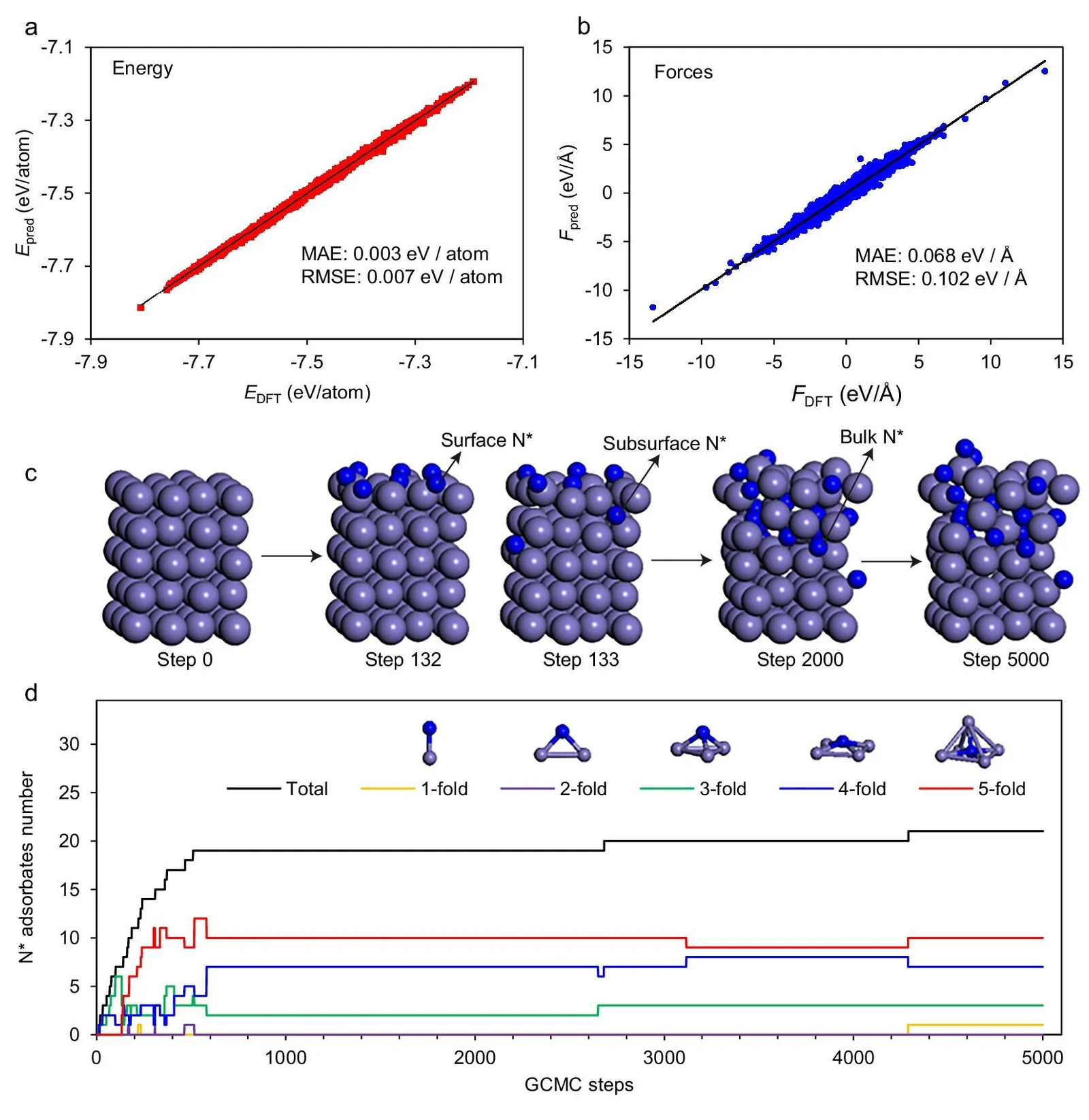
(a and b) Performances of energies and forces of MLP on testing sets vs. DFT. (c) Representative structural evolution of the Fe surface considering the adsorption and migration of N* at −0.5 V vs. RHE by MLP-driven GCMC. (d) Variation of total N* species and that with different coordination numbers with GCMC steps. The inserts display representative structures of N* with different coordination numbers.

In this section, the GCMC method was employed to sample the possible structures of Fe surface in eNORR, which was driven by MLP to accelerate the extensive structural optimizations. The costs of time of the MLP and DFT-driven GCMC simulation are compared in Fig. S8. Similar methods have been used in previous studies [28–30]. The MLP was trained by recursively embedded atom neural network (REANN) method [31,32] based on the datasets prepared primarily by AIMD simulations (Supporting Information S1.6, Table S3). The performances of the MLP on testing sets are shown in Fig. 2a and b. By MLP-driven GCMC (Supporting Information S1.7, Fig. S9), the N* adsorption and dynamic diffusion on Fe (110) facet was simulated under different electrode potentials, where the chemical potential (*μ*) of nitrogen was referenced to NO, H^+^+e^−^ and H_2_O (Supporting Information S1.8, Eq. S29). Figure 2c illustrates a representative structural evolution of Fe surface derived from a 5000-step GCMC simulation at −0.5 V vs. RHE. At the early stage from step 0 to 132, increasing N* atoms are adsorbed on the Fe surface, reaching a coverage of about 58%. Subsequently, the surface N* species begin to diffuse into the subsurface and bulk, which increases with the proceeding of GCMC simulation. Figure 2d shows the number of total N* species (black) vs. GCMC step, which increases quickly at the beginning stage while changing very sluggishly after 600 steps. In addition, the N* atoms with different coordination numbers are also analyzed in Fig. 2d, where the 1–4-fold N* can be mainly assigned to surface N* species, while 5-fold N* represents its location in subsurface or bulk. The 5-fold N* is about 1/2 of the total after 5000-step GCMC simulation. Furthermore, to obtain a statistical distribution of the diverse structures, we performed large-scale GCMC simulations at different applied potentials (−0.8, −0.5, −0.2, 0.1, and 0.4 V vs. RHE). Note that the MLP-GCMC simulations were primarily used to explore accessible stable and metastable configurations, rather than to establish a rigorously equilibrated ensemble, hence all GCMC simulations were run for 5000 steps. At every potential, 100 independent GCMC simulations were conducted, and the distribution of the number of added N atoms are presented in Fig. S11. By MLP-acceleration, 2.5 million structures have been explored during the large-scale GCMC simulations.

Given that Fe is oxyphilic and might undergo hydroxylation in aqueous electrolytes, we further investigated the competition between the formation of FeN*_x_* and Fe(OH)*_x_*. We first calculated the adsorption free energies (Δ*G*_ads_) of N* and OH* with different coverages (referenced to NO, H_2_O, and H^+^+e^−^) on Fe (110) surface at different potentials, which represents the first step of FeN*_x_* formation and hydroxylation of Fe, respectively. As shown in Fig. S12, the N* adsorptions with different coverages [0.25, 0.5, 0.75, and 1 monolayer (ML)] are all much more favorable than OH* adsorptions under −0.8 to 0.4 V vs. RHE, suggesting that the formation of FeN*_x_* should be preferred than hydroxylation. Nevertheless, the adsorptions of OH* are also exothermic when the potential > −0.6 V vs. RHE, suggesting that hydroxylation is also possible. Therefore, we further conducted GCMC to simulate the competitive accumulation of OH* and N* simultaneously. Since hydrogen was not included in our MLP, the GCMC simulations were driven by DFT calculations. We considered the potentials of −0.2, −0.5, and −0.8 V vs. RHE, which are in the main potential window for ammonia production on Fe. Figure S13 presents the number of accumulated N* and OH* species vs. GCMC steps and corresponding surface structures. At −0.2 V vs. RHE (Fig. S13a), although the adsorptions of OH* and N* occur simultaneously, the ratio of OH*/N* is about 1/4 after 2500 GCMC-steps, indicating the dominant behavior of nitridation. Moreover, at −0.5 and −0.8 V vs. RHE (Fig. S13b and c), only nitridation occurs while the OH* adsorption is not observed in the GCMC simulation. These results agree well with the energy trends of OH* and N* adsorption (Fig. S12). As the applied potential becomes more negative, the adsorption of OH* is more endothermic, leading to the elimination of hydroxylation at −0.5 and −0.8 V vs. RHE. Since −0.5 V is the optimal potential for ammonia production on Fe, we mainly investigated the effects of FeN*_x_* formation on the surface structure and its catalytic nature in the following studies.

### Identification of active sites

**Figure 3.**
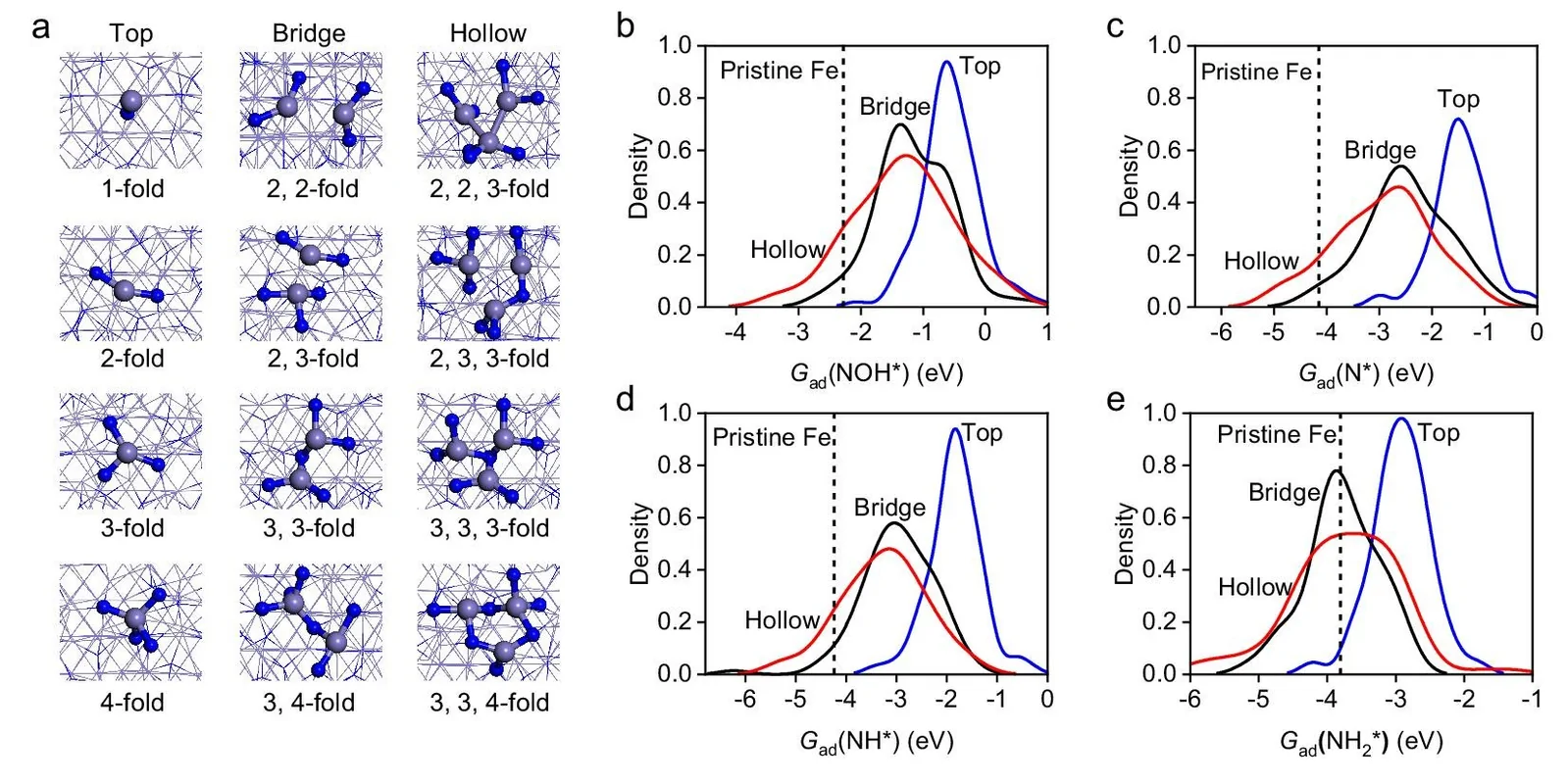
(a) Examples of the top, bridge, and hollow sites with different N coordination numbers on FeN*_x_* motifs. (b–e) Kernel density estimate of *G*_ad_ of NOH* (b), N* (c), NH* (d), and NH_2_* (e) on a hundred of top, bridge, and hollow sites, respectively. The vertical dashed lines represent adsorption energy on pristine Fe.

During structural evolution of nitridation, the surface Fe atoms can coordinate with N*, generating different FeN*_x_* motifs and reaction sites with diverse local structures, including top, bridge, and hollow sites, as shown in Fig. S14. Moreover, for each type of reaction site, the N coordination number of Fe can also be different, ranging from 1 to 4; thus a variety of reaction sites can be acquired (see Fig. 3a for some examples). We selected a hundred of top, bridge, and hollow sites with various local structures, respectively, to investigate their eNORR activity to NH_3_. The adsorption free energies (*G*_ad_) of reaction intermediates (NOH*, N*, NH*, and NH_2_*) on these sites were calculated (Fig. S15, Table S4) and the Kernel density estimate (KDE) of *G*_ad_ were analyzed. As shown in Fig. 3b–e, on top sites (the blue), the distribution of *G*_ad_ of each intermediate is totally weaker than that on pristine Fe (vertical dashed line). At bridge (black) and hollow (red) sites, however, the peak positions of *G*_ad_ of different intermediates are all shifted to more negative values than that on top sites. It indicates the stronger adsorption of intermediates on the former two sites. The peak positions of *G*_ad_(NH_2_*) on bridge and hollow sites are even comparable with pristine Fe (Fig. 3e). In short, the low eNORR activity to NH_3_ on pristine Fe can be attributed to the strong adsorption affinity to intermediates, which causes the difficulty in successive protonation of N* to NH_3_. Therefore, the weakened intermediates adsorptions at top sites on FeN*_x_* motifs can primarily increase the eNORR activity [33].

**Figure 4.**
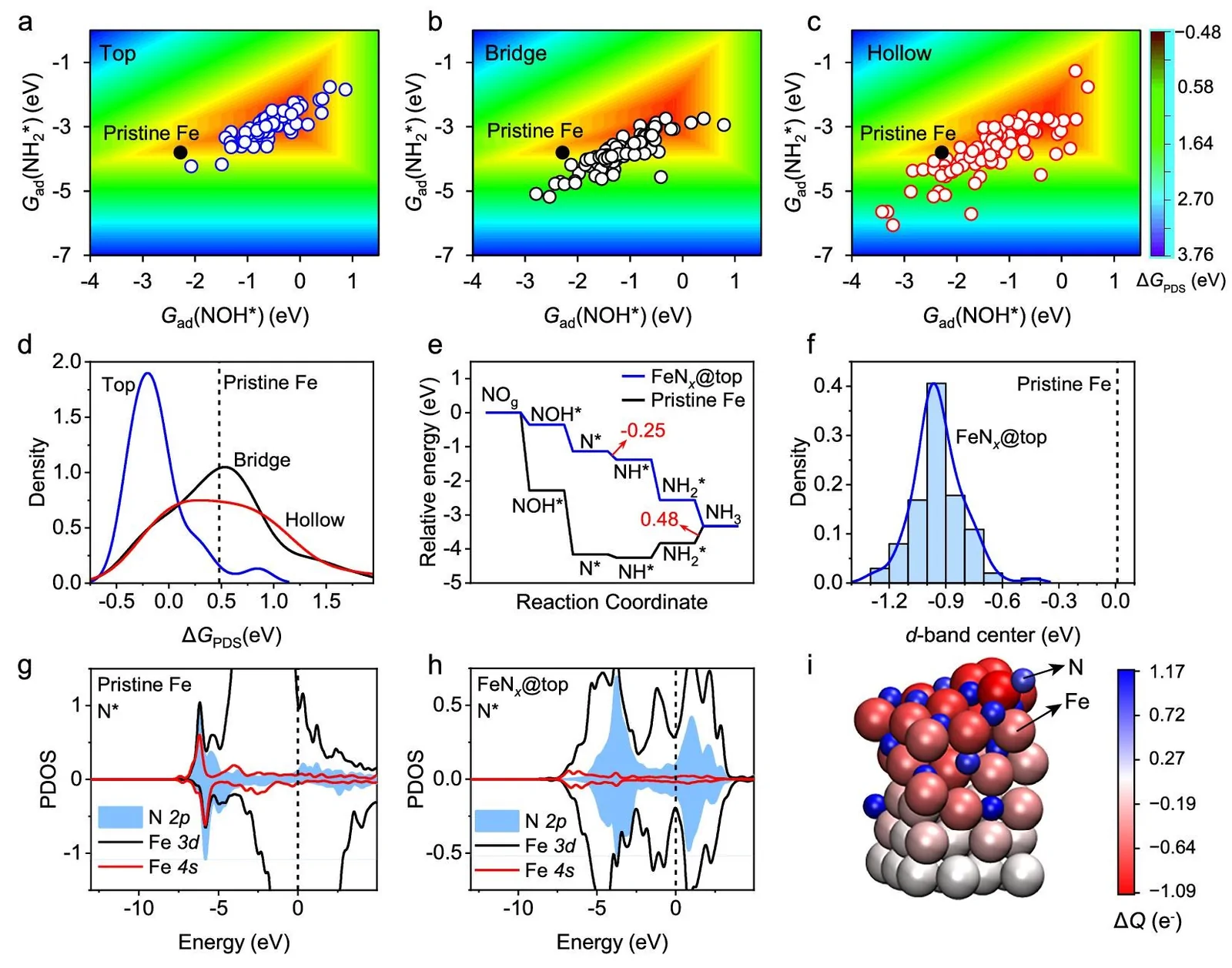
(a–c) (Quasi) activity map of eNORR to NH_3_ for top, bridge, and hollow sites of FeN*_x_* motifs using *G*_ad_(NOH*) and *G*_ad_(NH_2_*) as descriptors. (d) KDE analysis of Δ*G*_PDS_ over a hundred of top, bridge, and hollow sites of FeN*_x_* motifs, respectively. (e) Free energy diagrams of eNORR to NH_3_ on pristine Fe and FeN*_x_*@top. The numbers indicate corresponding Δ*G*_PDS_. (f) KDE analysis of ε_d_ of Fe atoms for different FeN*_x_*@top. (g and h) PDOS of Fe and adsorbed N* on pristine Fe and FeN*_x_*@top. The dashed line represents Fermi-level. (i) Bader charge analysis for FeN*_x_* motifs. Red and blue represent loss and acceptance of electrons, respectively.

The eNORR activity of the 100 top, bridge, and hollow sites of FeN*_x_* motifs were depicted using *G*_ad_(NOH*) and *G*_ad_(NH_2_*) as the descriptors (Fig. 4a–c). For top sites (Fig. 4a), almost all of them exhibit higher eNORR activity than pristine Fe and most of them are located around the peak area of activity, explaining the high NH_3_ yield rate in experiments. For bridge and hollow sites, some of their activities are improved, while the others are comparable or inferior to pristine Fe (Fig. 4b and c). To further elucidate the active sites of FeN*_x_* motifs for eNORR to NH_3_, we plot the KDE distribution of calculated Δ*G*_PDS_ of top, bridge and hollow sites in Fig. 4d (see details in Table S5). As shown, the top sites boost the activity remarkably with the density peak of Δ*G*_PDS_ at about −0.25 eV, much superior to pristine Fe (Δ*G*_PDS_ = 0.48 eV). While the peak positions of Δ*G*_PDS_ of bridge and hollow sites are very close to that of pristine Fe, indicating the similar eNORR activity. Therefore, the top sites on FeN*_x_* motifs (denoted as FeN*_x_*@top) are identified as more active sites than bridge and hollow sites for eNORR on Fe electrode. Figure 4e compares the free energy changes of eNORR to NH_3_ on pristine Fe and a representative FeN*_x_*@top site at 0 V vs. RHE. On pristine Fe, the protonation of NH* and NH_2_* are endothermic, while all elementary steps are exothermic on FeN*_x_*@top owing to the weaker adsorption to intermediates. To understand the electronic origin of enhanced activity of FeN*_x_*@top, the *d*-band center (ε_d_) [34] of Fe atoms of the 100 top sites were computed. As shown in Fig. 4f, the Kernel density peak of ε_d_ of FeN*_x_*@top occurs at −0.95 eV, much lower than that of pristine Fe (0.01 eV), explaining the generally weaker adsorption affinity of FeN*_x_*@top. Specifically, the projected density of states (PDOS) indicates the N 2*p* electron of adsorbed intermediates (NOH*, N*, NH*, and NH_2_*) can not only interact strongly with Fe 3*d*, but also Fe 4*s* states (Figs 4g and S16a–c) on pristine Fe. On FeN*_x_*@top, however, the interactions between N 2*p* and Fe 4*s* states are declined (Figs 4h and S16d–f), weakening the Fe–N bonding strength. The Bader charge analysis indicates electrons will transfer from Fe to N atom on FeN*_x_* motifs (Fig. 4i), which may cause the loss of Fe 4*s* electrons and decline the bonding between Fe 4*s* and N 2*p* states.

### In situ self-single-atomization

**Figure 5.**
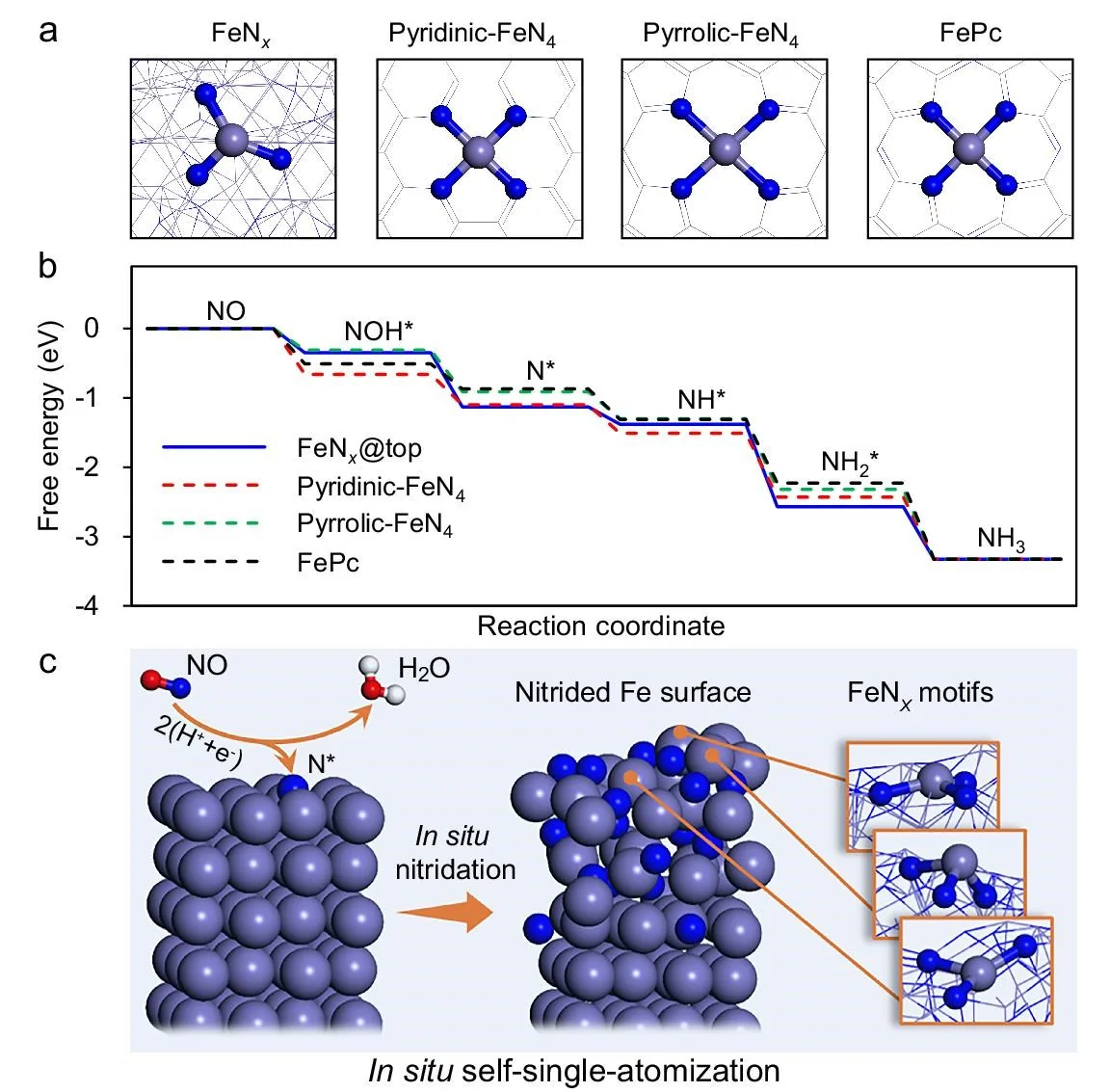
(a) Local active center of FeN*_x_* motifs, graphene-supported pyridinic-FeN_4_, pyrrolic-FeN_4_, and FePc SACs. (b) Free energy diagrams of eNORR on FeN*_x_*, pyridinic-FeN_4_, pyrrolic-FeN_4_, and FePc SACs. (c) Scheme illustration of *in situ* construction of FeN*_x_* single-atom catalysts on the Fe electrode during the eNORR process.

As shown in Fig. 5a, the isolated FeN*_x_* motifs display similar local structures and catalytic characteristics with the single-atom catalysts (SACs) because the top sites of FeN*_x_* are primary active sites. To further verify the SACs-like catalytic characteristics of FeN*_x_* motifs, we compared the free energy profiles of eNORR on a representative FeN*_x_*@top site with a graphene-supported pyridinic- and pyrrolic-FeN_4_ SAC, and a Fe phthalocyanine (FePc) SAC, as shown in Fig. 5b. For each intermediate (NOH*, N*, NH*, and NH_2_*), the adsorption free energies on FeN*_x_*, pyridinic-FeN_4_, pyrrolic-FeN_4_, and FePc are similar with the difference below 0.35 eV. In addition, the adsorption configurations of different intermediates on FeN*_x_* are also similar with that on FeN_4_ SACs (Fig. S17). Moreover, the Δ*G*_PDS_ of eNORR on FeN*_x_*@top (−0.25 eV) is also close to that on pyrrolic-FeN_4_ (−0.31 eV) and FePc (−0.36 eV). Therefore, the FeN*_x_* motifs can be viewed as *in situ* constructed single-atom catalysts on Fe electrode surface during the eNORR process, as illustrated in Fig. 5c, which boosts the eNORR activity significantly. Note that although the generated FeN_*x*_ centers show similar structures to the typical Fe–N–C SACs, the minor Fe–Fe interactions might still persist at the reconstructed surface.

### Potential-dependent selectivity on FeN_*x*_ motifs

In experiments, the selectivity of different products (N_2_O, N_2_, NH_3_, and H_2_) of eNORR exhibited a potential-dependent behavior on Fe electrode and a high FE of NH_3_ was obtained at −0.6 to −0.4 V vs. RHE (Fig. S1b). Our previous studies demonstrated that reaction kinetics plays a critical role in the selectivity regulation of eNORR [24,35–37]; hence we calculated the relevant reaction barriers of elementary steps in eNORR on a representative FeN*_x_*@top site. For electrochemical steps, the energy barriers were calculated via the EFC–CP method (Supporting Information S1.3, Figs S18 and S19), which can explicitly incorporate the effects of solvent and electric field. For NH_3_ production, the rate-determining step (RDS) is N* protonation on pristine Fe, while changes to NOH* protonation on FeN*_x_*@top (Fig. S20a) and the latter barrier is lower at various potentials (Fig. S20b), which leads to the higher reaction rate of eNORR to NH_3_ on FeN*_x_*@top (Fig. S20c). In addition, for N_2_O and N_2_ production, the thermochemical N–N coupling reactions are essential. These barriers were first evaluated via the climbing-image nudged elastic band (CI–NEB) method [38,39] using the vacuum surface (Fig. S21). As shown in Table S6, the different N–N coupling barriers on FeN*_x_* motifs are all significantly lower than that on pristine Fe, which is more reasonable to understand the high selectivity of N_2_O and N_2_ at low overpotential. Furthermore, since the reaction N* + NO_g_ → N_2_O* and N* + NOH* → N_2_OH* + * are more feasible (barrier < 0.15 eV) than the other N–N coupling reactions on FeN*_x_* motifs, the barriers of them were further verified by the EFC–CP method. As shown in Table S7, the N*–NO_g_ coupling is barrierless and the barriers of N*–NOH* coupling are in the range of 0.10 to 0.12 eV at different potentials, which are very close to the results from vacuum surface. Then the N*–NO_g_ and N*–NOH* couplings are considered as the possible mechanisms for N_2_O and N_2_ production in following microkinetic simulations. For hydrogen evolution reaction (HER), the Volmer–Heyrovsky mechanism was considered on FeN*_x_* motifs, and the bridge site was more thermodynamically preferred than top and hollow site (Fig. S22). Hence the bridge site was considered as active site to calculate the barriers of HER.

**Figure 6.**
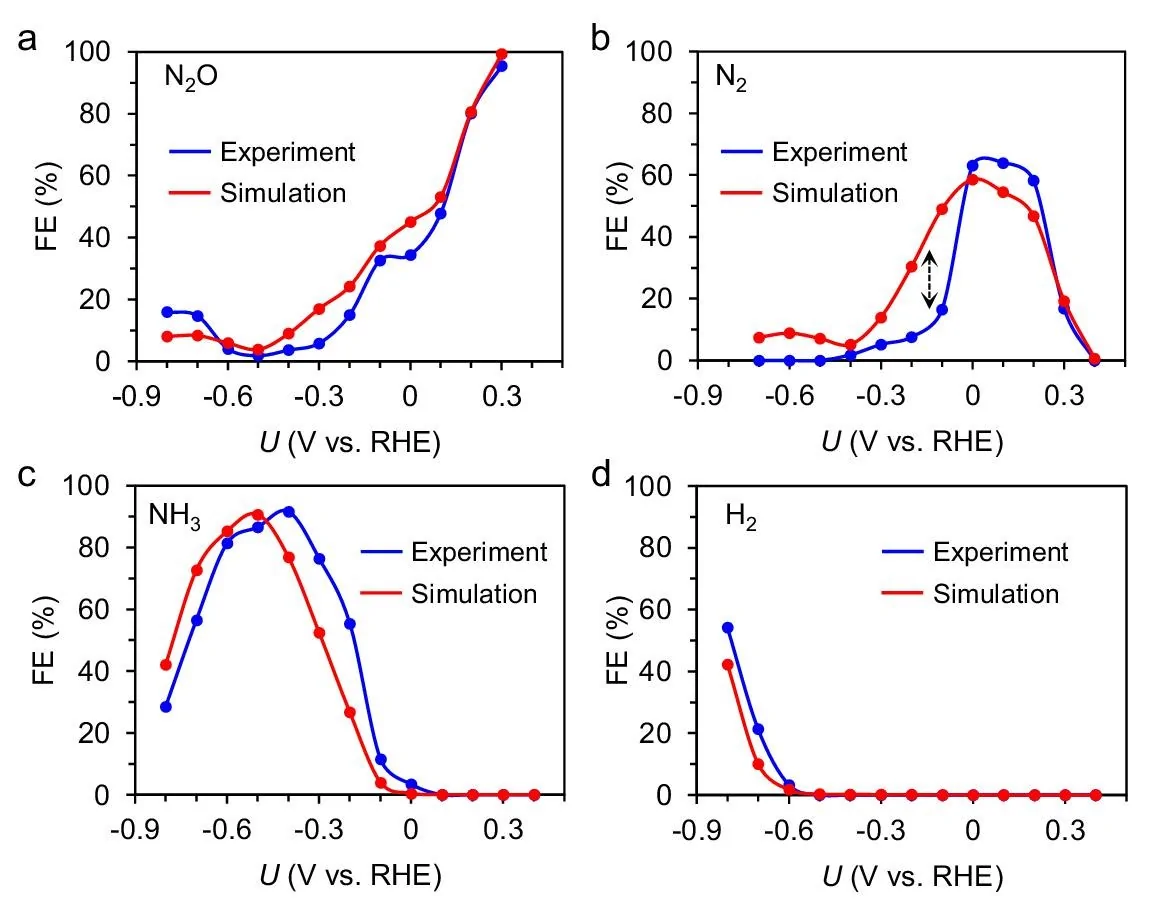
Comparisons between simulated FE of different products on FeN*_x_* motifs and experimental results. (a) N_2_O, (b) N_2_, (c) NH_3_, and (d) H_2_. The experimental data are adopted from ref [8].

Next, the microkinetic (MK) modeling (Supporting Information S1.4, Table S8) was performed at different potentials based on the calculated reaction free energies and barriers on FeN*_x_* motifs. According to the resulting turnover frequency (TOF) from MK, the FE of different products was further calculated with Equation S15, which reproduced the experimental selectivity trends successfully. As shown in Fig. 6a–d, with the change of applied potential, the calculated FE of N_2_O, N_2_, NH_3_, and H_2_ on FeN*_x_* motifs show qualitatively consistent trend compared with experimental results on Fe electrode. Specifically, in both theoretical modeling and experiment, the FE of N_2_O is declined gradually with the increase of overpotential (more negative potential) (Fig. 6a); the FE of N_2_ and NH_3_ exhibit parabola-like trends vs. potential, with the peaks at about 0 and −0.5 V vs. RHE, respectively (Fig. 6b and c); while the FE of H_2_ shows increasing trend below −0.6 V vs. RHE (Fig. 6d). The qualitative agreement between calculated and experimental selectivity trends of different products verifies the reliability of our active sites model. Nevertheless, there is a quantitative discrepancy between the experimental and simulated FE of N_2_ from 0.2 to −0.1 V vs. RHE (Fig. 6b). According to above study, the FeN*_x_* surface can adsorb a few OH* at −0.2 V vs. RHE, hence we further investigated the effect of adsorbed OH* on N*–NOH* coupling, which is responsible for N_2_ production. We found the N*–NOH* coupling barrier increases from 0.1 to 0.35 eV when a OH* is adsorbed on FeN*_x_* (Fig. S23), which explains the actually lower selectivity of N_2_ at −0.2 V in experiment.

To unveil the underlying reasons for potential-dependence of selectivity, we first analyzed the competition between N_2_O and N_2_ at low overpotential (0.1–0.4 V vs. RHE). As shown in Fig. S24a and b, the N_2_O and N_2_ production first follow the same pathway for NO_g_ reduction to N*, and then undergo the N*–NO_g_ and N*–NOH* coupling mechanisms, respectively. At different potentials, the barriers of N*–NO_g_ and N*–NOH* coupling nearly keep unchanged because these coupling processes are thermochemical reactions without proton and electron transfer. At 0.4 V vs. RHE, the coverage of adsorbed NOH* species (*θ*_NOH*_) is very low (Fig. S24c) since NOH* formation from NO_g_ is endothermic (Fig. S24a), causing a low reaction rate for N*–NOH* coupling (Fig. S24c) and N_2_ production. With the increase of overpotential, the NOH* formation becomes more exothermic (Fig. S24d), and the *θ*_NOH*_ increases gradually to 0.47 at 0.1 V vs. RHE (Fig. S24c), which improves the N*–NOH* coupling rate significantly (Fig. S24c). As a result, the N_2_ selectivity increases while N_2_O selectivity declines from 0.4 to 0.1 V vs. RHE (Fig. 6a and b). Then, with the further increase of overpotential, the FE of NH_3_ begins to increase while N_2_ decreases below 0.1 V vs. RHE (Fig. 6b and c). As shown in Fig. S25a–c, the competition between NH_3_ and N_2_ production is mainly determined by the competition between N* protonation and N*–NOH* coupling. With more negative potentials, the barrier of N* protonation (leading to NH_3_ production) decreases gradually since it is an electrochemical reaction, while the barrier of thermochemical N*–NOH* coupling (leading to N_2_ production) keeps nearly unchanged (Fig. S25d). Hence the selectivity of NH_3_ will increase with more negative potentials while N_2_ selectivity will decrease. The N* protonation barrier becomes lower than N*–NOH* coupling below −0.2 V vs. RHE, at which the FE of NH_3_ surpasses N_2_ and reaches the optimal (92%) at about −0.5 V vs. RHE (Fig. 6c). Below −0.5 V vs. RHE, the NH_3_ FE begins to decrease while HER becomes increasingly predominant (Fig. 6c and d). We compared the barriers of the rate-determining step of HER and NH_3_ production at varying potentials, which are Volmer reaction [(H^+^+e^−^) + * → H*] and NOH* protonation [NOH* + (H^+^+e^−^) → N* + H_2_O], respectively. As shown in Fig. S26, although the barrier of Volmer reaction and NOH* protonation are both decreased with more negative potentials, the former exhibits a higher slope than the latter, which can be attributed to the high charge transfer coefficient (*β*) of Volmer reaction as we have revealed in previous study [36]. Therefore, the Volmer reaction becomes more competitive and the FE of HER increases at more negative potentials. According to the above discussions, the underlying reasons for the variation of eNORR selectivity with potential are understood well. Overall, we have rationalized the outstanding activity and potential-dependent selectivity of eNORR to NH_3_ on Fe electrode based on the FeN*_x_* motifs obtained from GCMC simulations.

## CONCLUSIONS

In this work, we first studied eNORR on pristine Fe, and found that it shows low activity for NH_3_ production due to the strong adsorption affinity to reaction intermediates, contrary to previous experiments. By microkinetic modeling, we found the Fe surface will be covered by a monolayer of adsorbed N* species in the reaction process, which will migrate to the Fe subsurface according to AIMD simulation. Hence, we further investigated the dynamic structural evolution of Fe surface by MLP-accelerated GCMC simulations. Through 500 independent GCMC simulations, we found the Fe surface will be self-single-atomized in the eNORR process, generating diverse surface structures and reaction sites with different atomic environments. The top sites of the single-atomized FeN*_x_* motifs were identified as the main active sites, which boost the eNORR activity significantly, explaining the great performance of Fe in experiments. At last, based on the self-single-atomized FeN*_x_* sites, we calculated the electrochemical barriers of eNORR and conducted microkinetic modeling, which reproduced the potential-dependent selectivity of N_2_O, N_2_, NH_3_, and H_2_ in experiments, verifying the reliability of the single-atomized active site. Overall, we have unveiled the self-adaptive capability of Fe under working conditions via self-single-atomization, which inspires more rational design of active sites beyond ammonia synthesis.

## Supplementary Material

nwag417_Supplemental_File

## References

[1] Andersen SZ, Colic V, Yang S et al. A rigorous electrochemical ammonia synthesis protocol with quantitative isotope measurements. Nature 2019; 570: 504–8. 10.1038/s41586-019-1260-x 31117118

[2] Long J, Li H, Xiao J. The progresses in electrochemical reverse artificial nitrogen cycle. Curr Opin Electrochem 2023; 37: 101179. 10.1016/j.coelec.2022.101179

[3] Long J, Chen S, Zhang Y et al. Direct electrochemical ammonia synthesis from nitric oxide. Angew Chem Int Ed 2020; 59: 9711–8. 10.1002/anie.202002337 32189423

[4] Ko BH, Hasa B, Shin H et al. Electrochemical reduction of gaseous nitrogen oxides on transition metals at ambient conditions. J Am Chem Soc 2022; 144: 1258–66. 10.1021/jacs.1c10535 35014265

[5] Vervloessem E, Aghaei M, Jardali F et al. Plasma-based N_2_ fixation into NO*_x_*: insights from modeling toward optimum yields and energy costs in a gliding arc plasmatron. ACS Sustain Chem Eng 2020; 8: 9711–20. 10.1021/acssuschemeng.0c01815

[6] Patil BS, Peeters FJJ, van Rooij G J et al. Plasma assisted nitrogen oxide production from air: using pulsed powered gliding arc reactor for a containerized plant. AlChE J 2017; 64: 526–37. 10.1002/aic.15922

[7] Kim D, Shin D, Heo J et al. Unveiling electrode–electrolyte design-based NO reduction for NH_3_ synthesis. ACS Energy Lett 2020; 5: 3647–56. 10.1021/acsenergylett.0c02082

[8] Cheon S, Kim WJ, Kim DY et al. Electro-synthesis of ammonia from dilute nitric oxide on a gas diffusion electrode. ACS Energy Lett 2022; 7: 958–65. 10.1021/acsenergylett.1c02552

[9] Wang D, Fan G, Luan D et al. Ru-incorporation-induced phase transition in Co nanoparticles for low-concentration nitric oxide electroreduction to ammonia at low potential. Adv Mater 2024; 36: 2408580. 10.1002/adma.202408580 39506426

[10] Meng J, Cheng C, Wang Y et al. Carbon support enhanced mass transfer and metal–support interaction promoted activation for low-concentrated nitric oxide electroreduction to ammonia. J Am Chem Soc 2024; 146: 10044–51. 10.1021/jacs.4c00898 38557014

[11] Lin Y, Liang J, Li H et al. Bi nanodendrites for highly efficient electrocatalytic NO reduction to NH_3_ at ambient conditions. Mater Today Phys 2022; 22: 100611. 10.1016/j.mtphys.2022.100611

[12] Bunea S, Coppens M, Urakawa A. Electrochemical conversion of NO to NH_3_ in a PEM cell. ACS Catal 2023; 13: 11345–51. 10.1021/acscatal.3c01600

[13] Li Y, Cheng C, Han S et al. Electrocatalytic reduction of low-concentration nitric oxide into ammonia over Ru nanosheets. ACS Energy Lett 2022; 7: 1187–94. 10.1021/acsenergylett.2c00207

[14] Chen K, Zhang Y, Xiang J et al. p-Block antimony single-atom catalysts for nitric oxide electroreduction to ammonia. ACS Energy Lett 2023; 8: 1281–8. 10.1021/acsenergylett.2c02882

[15] Li Z, Ma Z, Liang J et al. MnO_2_ nanoarray with oxygen vacancies: an efficient catalyst for NO electroreduction to NH_3_ at ambient conditions. Mater Today Phys 2022; 22: 100586. 10.1016/j.mtphys.2021.100586

[16] Li Z, Zhou Q, Liang J et al. Defective TiO_2−_*_x_* for high-performance electrocatalytic NO reduction toward ambient NH_3_ production. Small 2023; 19: 2300291. 10.1002/smll.202300291 36919558

[17] Liu P, Liang J, Wang J et al. High-performance NH_3_ production via NO electroreduction over a NiO nanosheet array. Chem Commun 2021; 57: 13562–5. 10.1039/D1CC06113E 34842863

[18] Mou T, Liang J, Ma Z et al. High-efficiency electrohydrogenation of nitric oxide to ammonia on a Ni_2_P nanoarray under ambient conditions. J Mater Chem A 2021; 9: 24268–75. 10.1039/D1TA07455E

[19] Liang J, Zhou Q, Mou T et al. FeP nanorod array: a high-efficiency catalyst for electroreduction of NO to NH_3_ under ambient conditions. Nano Res 2022; 15: 4008–13. 10.1007/s12274-022-4174-0

[20] Zhang L, Liang J, Wang Y et al. High-performance electrochemical NO reduction into NH_3_ by MoS_2_ nanosheet. Angew Chem Int Ed 2021; 60: 25263–8. 10.1002/anie.202110879 34519397

[21] Zhang L, Zhou Q, Liang J et al. Enhancing electrocatalytic NO reduction to NH_3_ by the CoS nanosheet with sulfur vacancies. Inorg Chem 2022; 61: 8096–102. 10.1021/acs.inorgchem.2c01112 35535516

[22] Liang J, Liu P, Li Q et al. Amorphous boron carbide on titanium dioxide nanobelt arrays for high-efficiency electrocatalytic NO reduction to NH_3_. Angew Chem Int Ed 2022; 61: e202202087. 10.1002/anie.202202087 35212442

[23] Ouyang L, Zhou Q, Liang J et al. High-efficiency NO electroreduction to NH_3_ over honeycomb carbon nanofiber at ambient conditions. J Colloid Interface Sci 2022; 616: 261–7. 10.1016/j.jcis.2022.02.074 35219191

[24] Li H, Long J, Jing H et al. Steering from electrochemical denitrification to ammonia synthesis. Nat Commun 2023; 14: 112. 10.1038/s41467-023-35785-w PMC9825404 36611030

[25] Fu X, Li J, Long J et al. Understanding the product selectivity of syngas conversion on ZnO surfaces with complex reaction network and structural evolution. ACS Catal 2021; 11: 12264–73. 10.1021/acscatal.1c02111

[26] Luan D, Xiao J. Adaptive electric fields embedded electrochemical barrier calculations. J Phys Chem Lett 2023; 14: 685–93. 10.1021/acs.jpclett.2c03588 36638320

[27] Long J, Luan D, Fu X et al. Theoretical design of the electrocatalytic urea synthesis from carbon dioxide and nitric oxides. ACS Catal 2024; 14:, 14678–87. 10.1021/acscatal.4c03785

[28] Yang C, Fu X, Luan D et al. Towards rational design of confined catalysis in carbon nanotube by machine learning and grand canonical monte carlo simulations. Angew Chem Int Ed 2025; 64**:** e202421552. 10.1002/anie.202421552 39803774

[29] Chen L, Tian Y, Hu X et al. Constant-potential MD with neural network potentials reveals cation effects on CO_2_ reduction at Au-water interfaces. JACS Au 2025; 6: 868–80. 10.1021/jacsau.5c01198 PMC12933322 41755859

[30] Sun M, Jin B, Yang X et al. Probing nuclear quantum effects in electrocatalysis via a machine-learning enhanced grand canonical constant potential approach. Nat Commun 2025; 16: 3600. 10.1038/s41467-025-58871-7 PMC12000419 40234425

[31] Zhang Y, Xia J, Jiang B. Physically motivated recursively embedded atom neural networks: incorporating local completeness and nonlocality. Phys Rev Lett 2021; 127: 156002. 10.1103/PhysRevLett.127.156002 34677998

[32] Zhang Y, Xia J, Jiang B. REANN: a PyTorch-based end-to-end multi-functional deep neural network package for molecular, reactive, and periodic systems. J Chem Phys 2022; 156: 114801. 10.1063/5.0080766 35317591

[33] Medford AJ, Vojvodic A, Hummelshøj JS et al. From the Sabatier principle to a predictive theory of transition-metal heterogeneous catalysis. J Catal 2015; 328: 36–42. 10.1016/j.jcat.2014.12.033

[34] Norskov JK, Abild-Pedersen F, Studt F et al. Density functional theory in surface chemistry and catalysis. Proc Natl Acad Sci USA 2011; 108: 937–43. 10.1073/pnas.1006652108 PMC3024687 21220337

[35] Guo P, Luan D, Li H et al. Computational insights on structural sensitivity of cobalt in NO electroreduction to ammonia and hydroxylamine. J Am Chem Soc 2024; 146: 13974–82. 10.1021/jacs.4c01986 38723620

[36] Long J, Guo C, Fu X et al. Unveiling potential dependence in NO electroreduction to ammonia. J Phys Chem Lett 2021; 12: 6988–95. 10.1021/acs.jpclett.1c01691 34283618

[37] Li L, Luan D, Long J et al. Understanding the selectivity differences of NO electroreduction on Ag and Au electrodes. J Phys Chem Lett 2025; 16: 3447–53. 10.1021/acs.jpclett.5c00656 40152685

[38] Henkelman G, Jónsson H. Improved tangent estimate in the nudged elastic band method for finding minimum energy paths and saddle points. J Chem Phys 2000; 113: 9978–85. 10.1063/1.1323224

[39] Henkelman G, Uberuaga BP, Jónsson H. A climbing image nudged elastic band method for finding saddle points and minimum energy paths. J Chem Phys 2000; 113: 9901–4. 10.1063/1.1329672

