# Supplementary material for: Self-single-atomization of active sites in electrochemical ammonia synthesis unveiled by machine learning potential
Source: Natl Sci Rev. 2026 Jul 9;13(17):nwag417. doi: 10.1093/nsr/nwag417 (PMC13591637; doi:10.1093/nsr/nwag417)
Supplement: Supplementary file 1 [file nwag417_supplemental_file.pdf]

Supporting Information for

Self-single-atomization of active sites in electrochemical ammonia synthesis unveiled by machine learning potential

Jun Long[1], Chenyu Yang[1,2], Huan Li[1], Hao Li[1], Jianping Xiao[1,2,*]

1. State Key Laboratory of Catalysis, Dalian National Laboratory for Clean Energy, Dalian Institute of Chemical Physics, Chinese Academy of Sciences, Zhongshan Road 457, Dalian 116023, P. R. China.

2. University of Chinese Academy of Sciences, Beijing 100049, P. R. China.

This PDF file includes:

S1 to S3

Figure S1 to S26

Table S1 to S10

S1. Methodology

S1.1 Density functional theory (DFT) calculations

In this study, all the DFT calculations were conducted via the Vienna Ab initio Simulation Package (VASP)[1-2], with the exchange-correlation functional of revised Perdew – Burke – Ernzerhof (rPBE)[3] under the generalized-gradient approximation. The spin effect of electron was considered in all DFT calculations. The convergence criterion was set to 0.05 eV/Å for residual force. A cutoff energy of 400 eV was adopted with the Gaussian smearing width of 0.2 eV. The Van der Waals interactions were considered with the DFT-D2 method[4]. The solvation effect was considered for calculating the adsorption free energies of intermediates via the VASPsol method[5] (**Table S9**), with the dielectric constant of 78.5. For pristine Fe slab, a (3×3×1) of Monkhorst-Pack k-point mesh grid was employed for Brillouin zone sampling. A Fe (110) slab with the size of (3×3) and thickness of 4 layers (the bottom two layers were fixed in relaxation) was used to model the pristine Fe catalyst, and a vacuum (15 Å) was introduced to avoid the interaction between periodically adjacent slabs. For the other metals (Ag, Au, Cu, Pt, Pd, Rh, and Ru) in Figure 2a, the adsorption energies of intermediates were adopted from our previous study[6]. The transition states of elementary steps were directly computed by the method of climbing-image nudged elastic band (CI-NEB)[7-8] to determine the reaction barriers, which avoid the introduction of reorganization energy (λ). The adsorption free energies (G_{ad}) of intermediates 0 V vs. RHE were calculated referenced to NO, H_2, and H_2O. Firstly, we calculated the free energy potential (G) of H, O, and N referenced to the free energy of NO, H_2 and H_2O by following equations:

$$G(H) = 0.5G(H_2) \quad \text{(Eq. S1)}$$

$$G(O) = G(H_2O) - G(H_2) \quad \text{(Eq. S2)}$$

$$G(N) = G(NO) - [G(H_2O) - G(H_2)] \quad \text{(Eq. S3)}$$

Then, the G_{ad} of intermediates were calculated by:

$$G_{ad} = [E_{slab*ad} + ZPE - TS] - E_{slab} - [xG(N) + yG(O) + zG(H)] \quad \text{(Eq. S4)}$$

where the $E_{slab*ad}$ and E_{slab} represent the DFT energies of the slab with adsorbates and clean slab, respectively. ZPE and S are zero-point energy and entropy of the adsorbates. T is temperature. x, y, and z are the number of N, O, and H atoms in the adsorbates.

S1.2 Catalytic reaction phase diagram (CatRPD)

The CatRPD method[9] was adopted to estimate the activity trend and mechanism of different metal for eNORR to ammonia at thermodynamic level in this work, which has been applied successfully in eNORR study in our previous studies[10-11]. In CatRPD analysis, all possible reaction pathways of target reaction are first enumerated by an algebraic algorithm according to the provided elementary steps. Then, according to the adsorption free energies (G_{ad}) of intermediates, the reaction free energies (ΔG) of all elementary steps in each pathway can be calculated. For a given pathway, the most endothermic elementary step can be identified via an internal comparison between every elementary step in this pathway, and its ΔG (ΔG_{max}) can describe the activity of this pathway. After that, the minimum ΔG_{max} identified from external comparison among all possible pathways (defined as ΔG_{PDS}) can be used to describe the (quasi) activity of the reaction, as expressed in the following equation.

$$\Delta G_{PDS} = \min_i[\max_j(\Delta G_{(i,j)})] \qquad \text{(Eq. S5)}$$

where j and i represents the elementary steps of a given path and all possible paths, respectively. Furthermore, according to the scaling relations of G_{ad} (**Figure S2**), the ΔG_{RPD} can be described by the G_{ad} of important intermediates to construct the (quasi) activity map, as shown in Figure 2a. More details of this algorithm can be found in our previous works[9, 12].

S1.3 Electric field controlling constant potential (EFC-CP) calculations

S1.3.1 Framework of EFC-CP method. In this work, a "electric field controlling constant potential" method was employed to calculate the relevant electrochemical barriers, which was developed by our group recently[13]. In calculation, we added a water layer on the catalyst surface to simulate the electrochemical interface. In this case, the catalyst surface will be charged due to the spontaneous electron transfer from water layer to catalyst, inducing an intrinsic electric field ($\vec{E}_{int}$) inside Helmholtz layer. The $\vec{E}_{int}$ can be calculated with

$$\vec{E}_{int} = \vec{n}_z \frac{\sigma}{\epsilon_{HP}} \qquad \text{(Eq. S6)}$$

where the σ represents excess charge density on catalyst surface. ϵ_{HP} is the dielectric constant inside Helmholtz layer, which can be determined using a fitting method (**S1.3.2**), and $\vec{n}_z$ is the unit vector in z-direction. For conventional simulation of protonation step using CI-NEB

method, the surface charge density of electrode will vary with the proton transfer across Helmholtz layer, causing the variation of $\vec{E}_{int}$. In EFC-CP method, we applied a self-adaptive electric field ($\vec{E}_{app}$) in calculation to offset the change of $\vec{E}_{int}$ and keep the total electric field ($\vec{E}_{tot}$) constant according to the following equation.

$$\vec{E}_{tot} = \vec{E}_{app} + \vec{E}_{int} \quad \text{(Eq. S7)}$$

As there is a one-to-one correspondence between electrode potential and $\vec{E}_{tot}$ inside Helmholtz layer, which is described by Bikerman−Poisson−Boltzmann (BPB) equation, the constant $\vec{E}_{tot}$ means constant potential. In this way, the constant potential calculations can be realized. More details of this method can be found in our previous studies[13-14]. For electrochemical barriers calculations, the EFC-CP method was incorporated to the CI-NEB framework to search the transition states at constant potential.

S1.3.2 Fitting dielectric constant (ϵ_{HP}). For a given solid-liquid interfaces model, the ϵ_{HP} is mainly determined by the specific chemical environments at the interface of the electrode. In this study, the N* protonation reaction [N*+(H^++e^-)→NH*] was selected as the model reaction to calculate the ϵ_{HP} of pristine Fe and FeN_x motifs. First, different values (4.5, 5.5, 6.5, and 7.5) of ϵ_{HP} were adopted to calculate the reaction energy (ΔE, energy difference between final and initial state) of N* protonation reaction. For each ϵ_{HP}, four different electrode potential ($\emptyset_M$), -1, -0.5, 0, and 0.5 V vs. potential of zero charge ($\emptyset_{PZC}$) were considered. In this case, we can obtain a linear relationship between ΔE and $\emptyset_M$ at each ϵ_{HP} as follow:

$$\Delta E = a\emptyset_M + b \quad \text{(Eq. S8)}$$

where the slope a and intercept b are dependent on ϵ_{HP}. The slope will be equal to the charge transfer (q) between initial and final states of the reaction if the Helmholtz model is assumed. However, in the calculation of ΔE, the charge of initial state is uncertain since the $H^{+\delta}$ is at the interfaces, not in the bulk solution. Hence a correction item ΔE^{corr} was applied to ΔE according to the Frumkin correction[15] as follow:

$$\Delta E^{corr} = q\emptyset_{rp} + (1 - q)(\emptyset_M - \emptyset_{PZC}) \quad \text{(Eq. S9)}$$

where $\emptyset_{rp}$ refers to the potential at the reaction plane. With ΔE^{corr}, the ΔE can be written as:

$$\Delta E = a^{corr}\emptyset_M + b^{corr} \quad \text{(Eq. S10)}$$

The corrected ΔE describes the energy change of a proton coupled electron transfer from bulk solution to electrode, hence a^{corr} should be equal to 1, which was used as the criterion to determining ϵ_{HP} (**Figure S3b** and **Figure S12b**).

S1.3.3 Internal reversible hydrogen electrode. In above, the mentioned electrode potential $\emptyset_M$ is referenced to potential of zero charge. To compare with experiment, we defined an internal reversible hydrogen electrode (IRHE), at which the explicitly calculated reaction free energy of hydrogen evolution reaction (ΔG_{HER}) is zero. Since the IRHE has an equivalent theoretical definition with the reversible hydrogen electrode (RHE), it is directly comparable with experimental potential. Moreover, according to our previous work[13], the model-dependence and dipole effect can be eliminated by using IRHE. To determine the $\emptyset_{IRHE}$, we calculated the ΔG_{HER} at different $\emptyset_M$ (vs. $\emptyset_{PZC}$) and a linear correlation between ΔG_{HER} and $\emptyset_M$ (vs. $\emptyset_{PZC}$) can be obtained, based on which the $\emptyset_{IRHE}$ can be extrapolated when $\Delta G_{HER} = 0$ (**Figure S3c** and **Figure S12c**).

S1.4 Microkinetic modelling

The microkinetic modelling in this study was performed using the Python-based Catalysis Rate Analysis Package (CatRAP) developed by our group recently. Based on the law of mass action, a general expression of reaction rate (r) for an elementary step is given as

$$r = k^+ \prod [R_i]^{v_i} - k^- \prod [P_i]^{v_i} \qquad \text{(Eq. S11)}$$

where v_i is the corresponding stoichiometric coefficient of component R_i (reactant) and P_i (product), k^+ and k^- are the forward and backward rate constants. According to the transition state theory, the $k^{+/-}$ can be derived with the following equation.

$$k = \frac{k_B T}{h} \exp\left(\frac{-\Delta G^{\neq,0}}{k_B T}\right) \qquad \text{(Eq. S12)}$$

where k_B, T, and h represent the Boltzmann constant, temperature, and Planck constant. And $\Delta G^{\neq,0}$ is the change in standard molar Gibbs free energy between the initial and transition state. In microkinetic modelling, all the catalytic sites were assumed to be equivalent, and a single site can only adsorb a single adsorbate. According to the mass balance for the fractional coverages, we can give

$$\sum_{i} \theta_i = 1 \quad \text{(Eq. S13)}$$

where θ_i is the fractional coverage of adsorbates or empty sites. A mean-field approximation was considered, so that the topology of catalyst surface can be neglected, and all the adsorbates are able to react with each other. The steady state approximation was also adopted in our calculation, which requires the change of coverages of reactive intermediates over time to be zero, as the following equation.

$$\frac{\partial \theta_i}{\partial t} = 0 \quad \text{(Eq. S14)}$$

In CatRAP, a modified Newton's method was employed to improve the solution precision of the constructed ordinary differential equations (ODEs), with an initial guess from the iteration of ODEs. The tolerance of ODE integration was set to be 1.0e-5, and the Newton's method was set to be 1.0e-100. When the iteration failed to converge, the algorithm would select the solution estimate from the iteration that achieved the minimum residual.

Based on the obtained TOF from microkinetic modelling, the FE of different products in eNORR can be calculated with the following equation.

$$FE_i(\%) = \frac{n_i TOF_i}{\sum n_i TOF_i} \times 100 \quad \text{(Eq. S15)}$$

where n_i refers to the required electron transfer number for the formation of product i.

S1.5 Ab initio molecular dynamics (AIMD) simulation

The AIMD simulation was performed to investigate structure reconstruction of Fe (110) surface after the adsorption of a monolayer of N* species. First, we optimized the adsorption of a monolayer of N* on the (4×3) slab of Fe (110) at 0 K (totally five layers with the bottom layer fixed). The converged atomic structure was used as the POSCAR of the AIMD simulation. The NVT ensemble and Nosé-Hoover thermostat were adopted in AIMD simulation, and the temperature was 298 K. The AIMD simulation ran 58 ps with the time step of 2 fs. The spin effect of electron was considered in simulation.

S1.6 Machine learning potential (MLP)

S1.6.1 MLP datasets. A MLP of Fe, N, and O system was trained to accelerate the GCMC simulation in this study. The datasets for training and test of MLP were mainly produced by the

AIMD simulations using Vienna Ab initio Simulation Package (VASP). By AIMD simulations, a variety of atomic structures and corresponding energies and forces can be obtained. In AIMD simulation, the exchange-correlation functional of revised Perdew–Burke–Ernzerhof (rPBE) was used. The cutoff energy was set to 400 eV. The spin effect of electron was considered. The NVT ensemble and Nosé-Hoover thermostat were adopted. The slabs of Fe (110) adsorbed by different number of N and/or O on the surface or subsurface were used as the initial structures of AIMD simulation. Different sizes of Fe (110) slab were considered, including (3×3×4), (4×4×4), and (4×4×5) slab, which have 36, 64, and 80 Fe atoms, respectively. Totally 45 independent AIMD simulations were conducted for these Fe-N-O structures at 500 or 1500 K. For each AIMD simulation, it ran 1000 steps (2ps) with the time step of 2 fs, among which we selected 320 and 80 structures to compose the training and test set, respectively. In addition, by random displacements of atoms, we produced 203 structures of Fe-N-O system as data augmentation, which represent relatively higher forces and energies, making the potential energy surface more general and robust. The energies and forces of these random structures were obtained by static calculation of VASP. Finally, the dataset contained 18203 data. The detailed information of the dataset was shown in **Table S3**.

S1.6.2 MLP training. The MLP was trained using recursively embedded atom neural network (REANN) model developed by Prof. Jiang et al.[16-17] based on the above dataset, which is an improved model based on the embedded atom neural network (EANN)[18] approach. In EANN method, the spatial distribution of the central atom and corresponding neighbors are described by a set of Gaussian-type orbitals (GTOs). The atomic descriptor, embedded atomic density (EAD), is generated by the square of the linear combination of GTOs (Eq. S16 – Eq. S17).

$$\varphi_{l_x l_y l_z}^{\alpha,r_s}(r) = x^{l_x} y^{l_y} z^{l_z} \, exp(-\alpha|r - r_s|^2) \quad \text{(Eq. S16)}$$

$$\rho_{L,\alpha,r_s}^{i} = \sum_{l_x,l_y,l_z}^{l_x+l_y+l_z=L} \frac{L!}{l_x! l_y! l_z!} \left(\sum_{j=1}^{n_{atom}} c_j \varphi_{l_x l_y l_z}^{\alpha,r_s}(r_{ij})\right)^2 \quad \text{(Eq. S17)}$$

where r represents the distance between central and neighbor atom. r_s, α, and L represent the center, width, and angular momenta of GTO, respectively. c_j is a coefficient depending on element. As indicated by Eq. S13, ρ^{i} is determined by these distances and angles because c_j is fixed after training. In REANN approach, the generated EADs are input to the atomic neural

network to calculate the atomic orbital coefficients when iteration number t < T, as the following equation.

$$c_j^t = g_j^{t-1}(\rho_j^{t-1}(c_j^{t-1}, r_j^{t-1})) \quad \text{(Eq. S18)}$$

The atomic energies are calculated as the following equation when t = T.

$$E_j = g_j^{T-1}(\rho_j^{T-1}(c_j^{T-1}, r_j^{T-1})) \quad \text{(Eq. S19)}$$

where $g_j^{T-1}$is the jth atomic neural network in tth iteration.

In this work, we used 8 Gaussian-type radial functions in REANN model to describe the atomic representation. The maximal angular momenta was set to 2. A hidden-layer architecture of (64×64×64×64) was employed for the neural network fitting atomic energy. The cutoff distance is 6 Å. 3 iterations were used to represent the orbital coefficients. The initial and final learning rate were 0.005 and 1×10^{-5}, respectively, and the decay factor of learning rate was 0.2. The energy coefficient (weight of energy), initial atomic forces coefficient (weight of atomic forces) and final atomic forces coefficient were set to 0.1, 50, and 0.5, respectively.

S1.7 Grand Canonical Monte Carlo (GCMC) simulation

The GCMC method were employed to simulate the nitridation of Fe surface in eNORR process. A (3×4) slab of Fe (110) surface with 5 atom layers (containing 60 Fe atoms) was employed as the initial structure, and the bottom layer atoms was fix in simulation. The flowchart of GCMC simulation is presented in **Figure S7**. Three possible Monte Carlo (MC) actions were considered, including adding N* adsorbate on Fe surface, migrating N* species from Fe surface to bulk, and moving N and Fe atoms randomly. For N adding steps, the N atoms were randomly placed on Fe surface with a distance of 1.6~2.0 Å to Fe atom. After each MC action, the structural optimization was performed using MLP and the energy of the atomic system was obtained. The acceptance probability (P) of the action was estimated according to the Metropolis-Hastings algorithm:

$$P = \begin{cases} 1, & \mu_{n+1} \leq \mu_n \\ \exp\left(-\frac{\mu_{n+1}-\mu_n}{k_B T}\right), & \mu_{n+1} > \mu_n \end{cases} \quad \text{(Eq. S20)}$$

where k_B and T are Boltzmann constant and temperature, respectively. The chemical potential (μ) of the system at step n (μ_n) is referenced to the environmental potential of N (μ_N), which is

calculated by:

$$\mu_n = E_n + \Delta x \mu_N \quad \text{(Eq. S21)}$$

where E_n is the calculated energy of the system at step n. Δx is the number of changed N atom at step n+1 relative to step n. μ_N is referenced to NO, (H^++e^-), and H_2O.

$$\mu_N = \mu_{NO} + 2\mu_{(H^+ + e^-)} - \mu_{H_2O} \quad \text{(Eq. S22)}$$

According to the computational hydrogen electrode (CHE) model[19], the chemical potential of proton-electron pair can be calculated as the following equation.

$$\mu_{(H^+ + e^-)} = \frac{1}{2}\mu_{H_2} - eU_{RHE} \quad \text{(Eq. S23)}$$

where U_{RHE} is the applied potential referenced to reversible hydrogen electrode (RHE). The GCMC simulations under different potential were conducted by changing the μ_N according to Eq. S22 and S23.

The steps of all independent GCMC simulations were set to be 5000 because the number of N* species added to Fe nearly keeps unchanged after 5000 steps. The barriers were not considered during the simulation of structural evolution by GCMC. Only the ground-states were identified using MLP in the GCMC simulation, and then the reaction barriers of electrochemical NO reduction were evaluated by DFT based on the FeN_x structures acquired from the GCMC. After the GCMC simulations, the coordination number of N* species with Fe was calculated based on the bond length cutoffs. If the bond length between two atoms $\leqslant R_A + R_B + 0.45$ (Å), they are judged to be bonded with each other, where R_A and R_B are the covalent radius of the two atoms, and 0.45 is an empirical bond length tolerance.

S1.8 Determination of the chemical potential of N in GCMC simulations

To explain how was the chemical potential of N (μ_N) in GCMC simulation determined, we performed analytical microkinetic modeling to calculate the production and consumption rate of N* species on Fe (110) surface at a given N* coverage. The elementary steps for N* production were considered as:

$$\text{R1: NO} + (H^+ + e^-) + * \rightarrow \text{NOH}*$$

$$\text{R2: NOH}* + (H^+ + e^-) \rightarrow \text{N}* + H_2O$$

The reaction rate of R1 (r_1) and R2 (r_2) can be written as:

$$r_1 = k^+ p_{NO} c_{(H^+)} \theta_* - k^- \theta_{NOH*} \quad \text{(Eq. 24)}$$

$$r_2 = k^+ \theta_{NOH*} c_{(H^+)} - k^- \theta_{N*} p_{H_2O} \quad \text{(Eq. 25)}$$

where k^+ and k^- are rate constant of the forward and backward reaction, respectively. θ represents coverage. The p_{NO} and $c_{(H^+)}$ are the partial pressure of NO and concentration of proton, respectively. The reaction energy barriers under different potentials were adopted from Table S2. The rate constant k was calculated by:

$$k = \frac{k_B T}{h} \exp\left(\frac{-G_a}{k_B T}\right) \quad \text{(Eq. 26)}$$

where k_B, T, h, and G_a denote the Boltzmann constant, temperature, Planck constant, and reaction energy barrier. To calculate the formation rate of N* at a given N* coverage, we assumed that the coverage of NOH* is at steady-state, thus we got:

$$\frac{\partial_{\theta_{NOH*}}}{\partial_t} = r_1 - r_2 = 0 \quad \text{(Eq. 27)}$$

In addition, the total coverage of intermediates and free site was assumed to be 1:

$$\theta_* + \theta_{NOH*} + \theta_{N*} = 1 \quad \text{(Eq. 28)}$$

Combining Eq. 24 to Eq. 28, we can obtain the production rates of N* at a given θ_{N*}. Then, for N* consumption, the elementary steps were considered as:

R3: $N* + (H^+ + e^-) \rightarrow NH*$

R4: $NH* + (H^+ + e^-) \rightarrow NH_2*$

R5: $NH_2* + (H^+ + e^-) \rightarrow NH_3 + *$

Employing the same assumptions and analogous equations, the consumption rates of N* were also calculated with the given θ_{N*}. The differences between the production (r_P) and consumption rates (r_C) of N* at various potentials and θ_{N*} were presented in **Figure S10**. As shown, at all considered potentials and θ_{N*}, the production rate of N* are much higher than the consumption rates by about $10^7 \sim 10^{10}$ S^{-1}, hence the consumption of N* is negligible in the formation process of FeN_x. In this case, we can assume the production of N*, namely the reaction of $NO + 2(H^+ + e^-) + * \rightarrow N* + H_2O$ is in equilibrium. Therefore, μ_N is referenced to NO, $(H^+ + e^-)$, and H_2O with the following equation:

$$\mu_N = \mu_{NO} + 2\mu_{(H^+ + e^-)} - \mu_{H_2O} \quad \text{(Eq. 29)}$$

S2. FeN_x structures after eNORR

When the applied potential is turned off, the electrochemical reactions are stopped, hence only thermochemical reactions are possible for the structural evolution of FeN_x. For adsorbed N* species, the following four possible thermochemical reactions were considered.

$$N* + NO \rightarrow N_2O + *$$

$$2N* \rightarrow N_2 + 2*$$

$$N* + H_2O \rightarrow NO + H_2 + *$$

$$4N* + 6H_2O \rightarrow 4NH_3 + 3O_2 + 4*$$

The chemical potential of N (μ_N) is determined to be -8.90, -8.36, -5.17, and -5.67 eV assuming the equilibrium of the four reactions, respectively. Using the above μ_N, we additionally performed GCMC to simulate the removal of N* from FeN_x. However, the number of N* species remains unchanged after the simulations under different μ_N, hence we concluded that the structures of generated FeN_x will remain stable when the potential is turned off.

S3. Discussing the formation of metal-N_x on the other metals during eNORR

According to our discussions about Fe, one of the prerequisites of the generation of metal-N_x structures is the accumulation of N* species on metal surface, which requires a strong N* adsorption affinity to facilitate NO reduction to N* while limit the further hydrogenation of N* to ammonia. Hence, the metals with stronger chemical reactivities than Fe are also likely to generate metal-N_x structures during eNORR, for example V, Cr, Mn, and Mo etc. For the metals less reactive than Fe, for example Co, Cu, and Ag, we additionally performed microkinetic modeling to investigate the intermediates coverages with the barriers and reaction free energies published in our previous works[20-22]. As shown in **Table S10**, the Co (111) surface is mainly covered by NH* and Cu (111) surface is mainly covered by NOH* and NH*. The coverage of N* species is negligible on both Co and Cu, hence it is difficult to generate CoN_x and CuN_x. In addition, on Ag (111), the free site (*) is dominant, hence Ag surface will remain bared during eNORR process and AgN_x will not be generated.

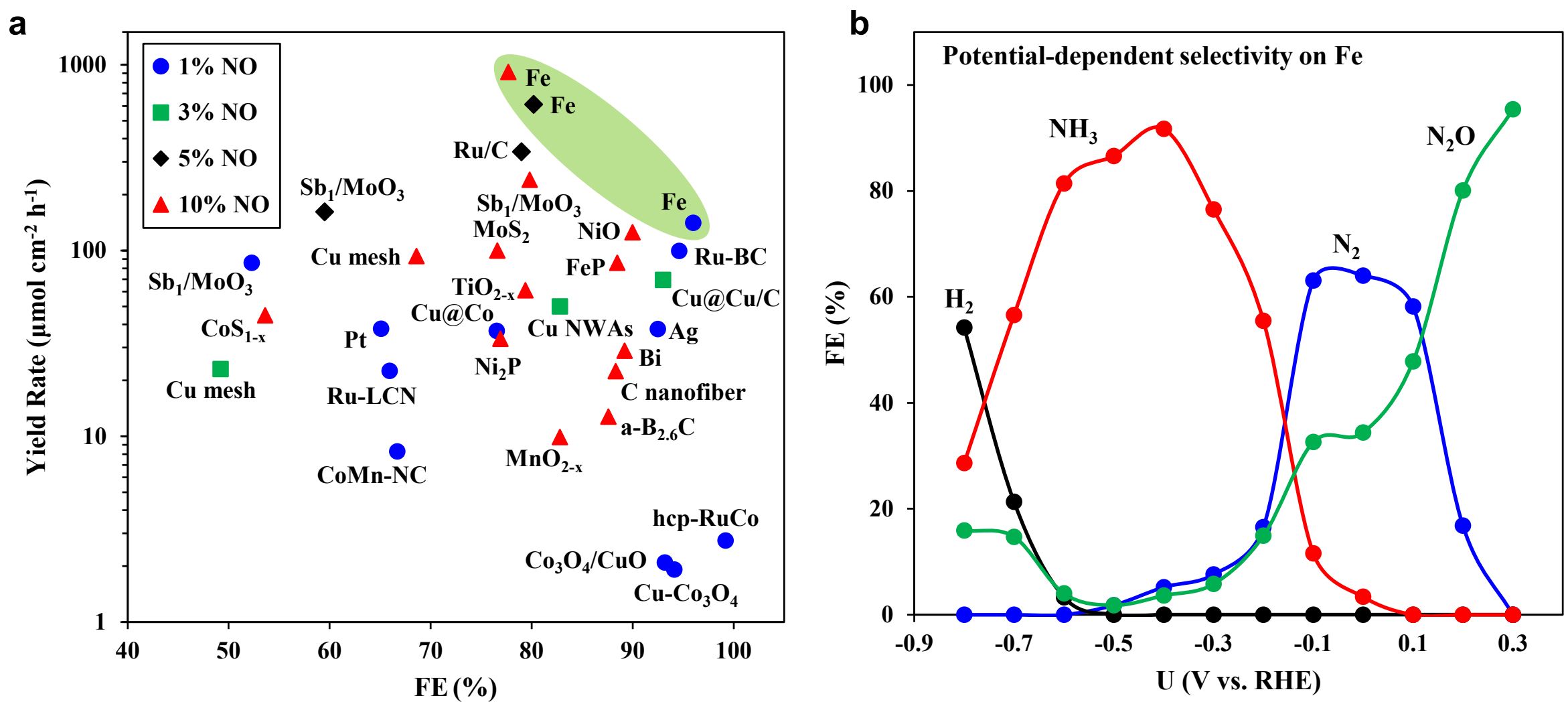

Figure S1. (a) Statistics of ammonia yield rate and FE for eNORR on various catalysts under low-concentration of NO. The data are adopted from ref.[23-39]. (b) Potential-dependent FE of different products of eNORR on Fe. The data are adopted from ref.[24].

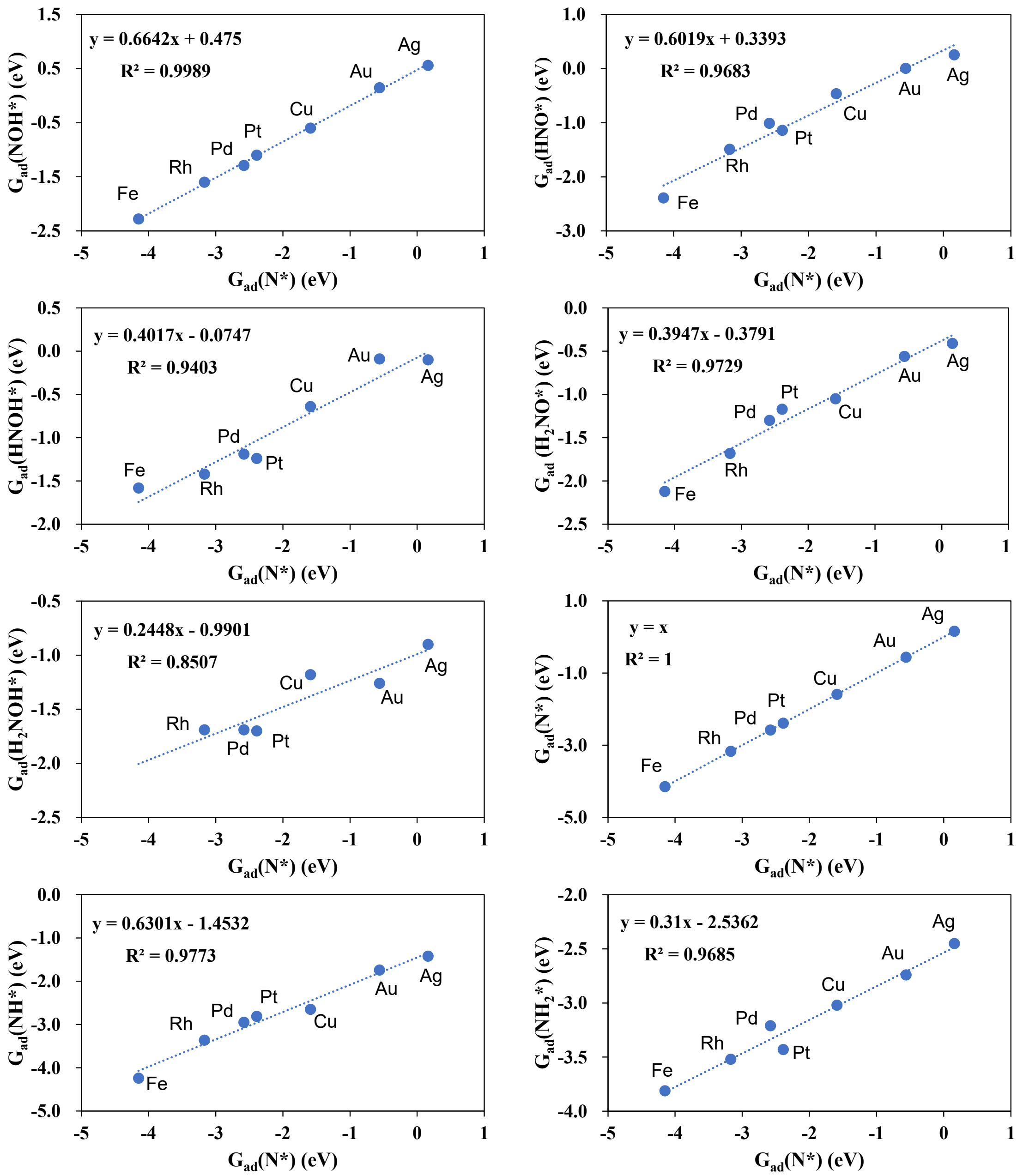

Figure S2. Scaling relations between adsorption free energies (G_{ad}) of N* and the other intermediates. Herein, the G_{ad} on Fe was calculated in this study, while the data of G_{ad} on the other metals (Ag, Au, Cu, Pt, Pd, Rh, and Ru) are recalculated from our previous work[6], referenced to the free energies of NO, H_2, and H_2O.

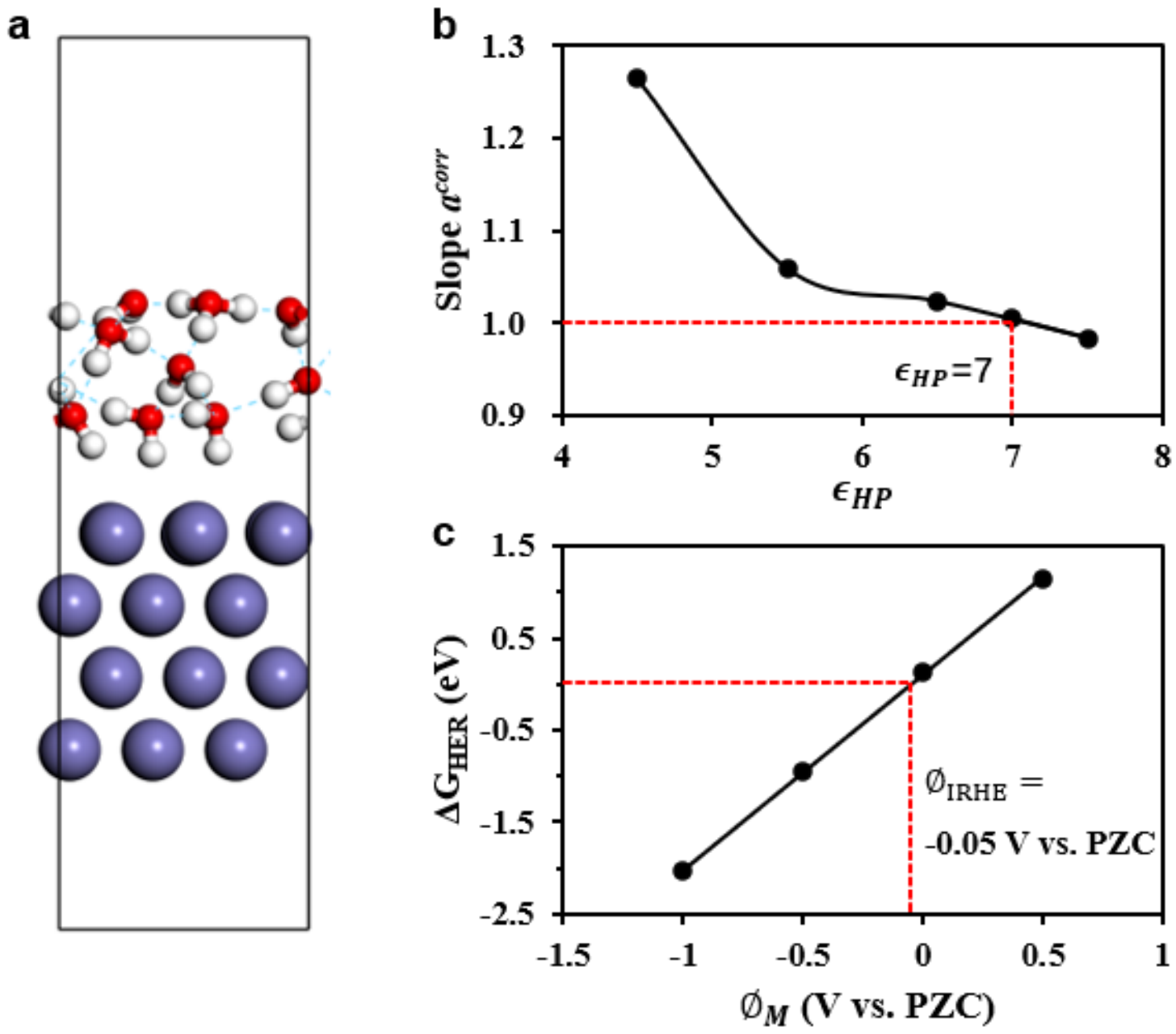

Figure S3. (a) Model of Fe (110) and water interface for electrochemical barriers calculation. (b) Fitting $\epsilon_{HP}$of Fe (110) and water interface. (c) Fitting IRHE of Fe (110) and water interface.

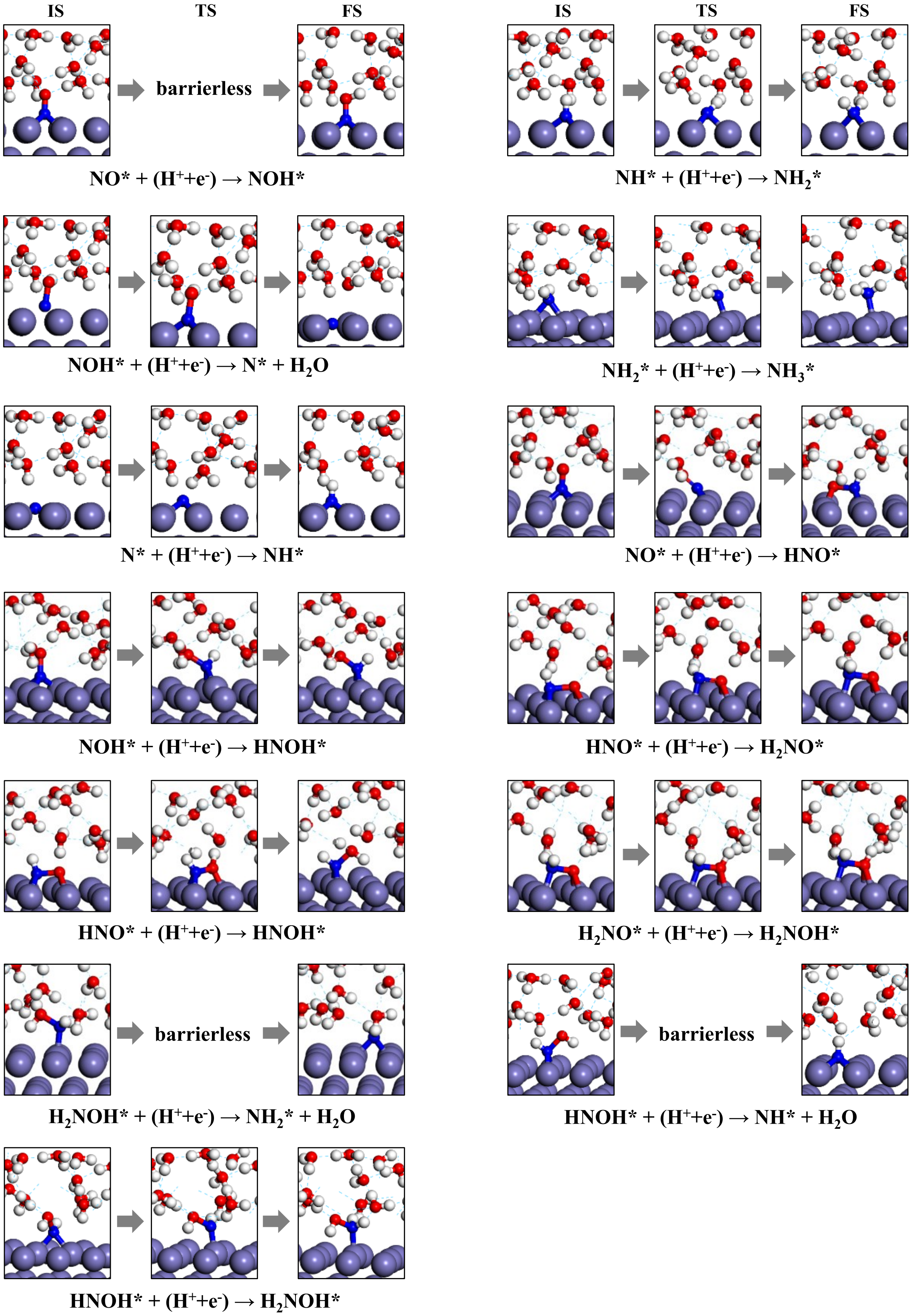

Figure S4. Atomic structures of the initial (IS), transition (TS), and final state (FS) for different elementary steps in eNORR to NH_3 on Fe (110).

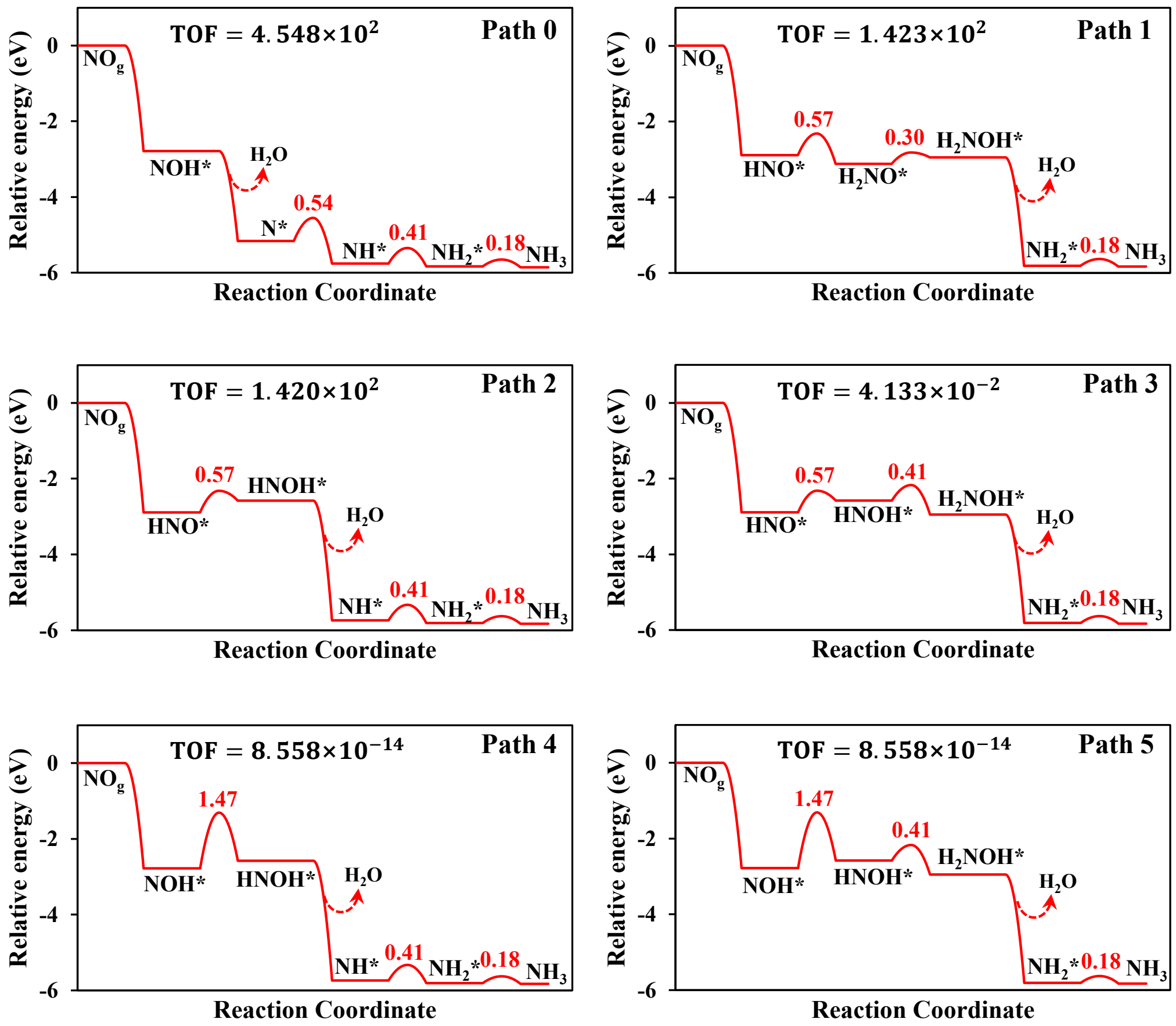

Figure S5. Free energy diagrams and corresponding TOF calculated by microkinetic modeling for eNORR to NH_3 on pristine Fe through different pathway at -0.5 V vs. RHE.

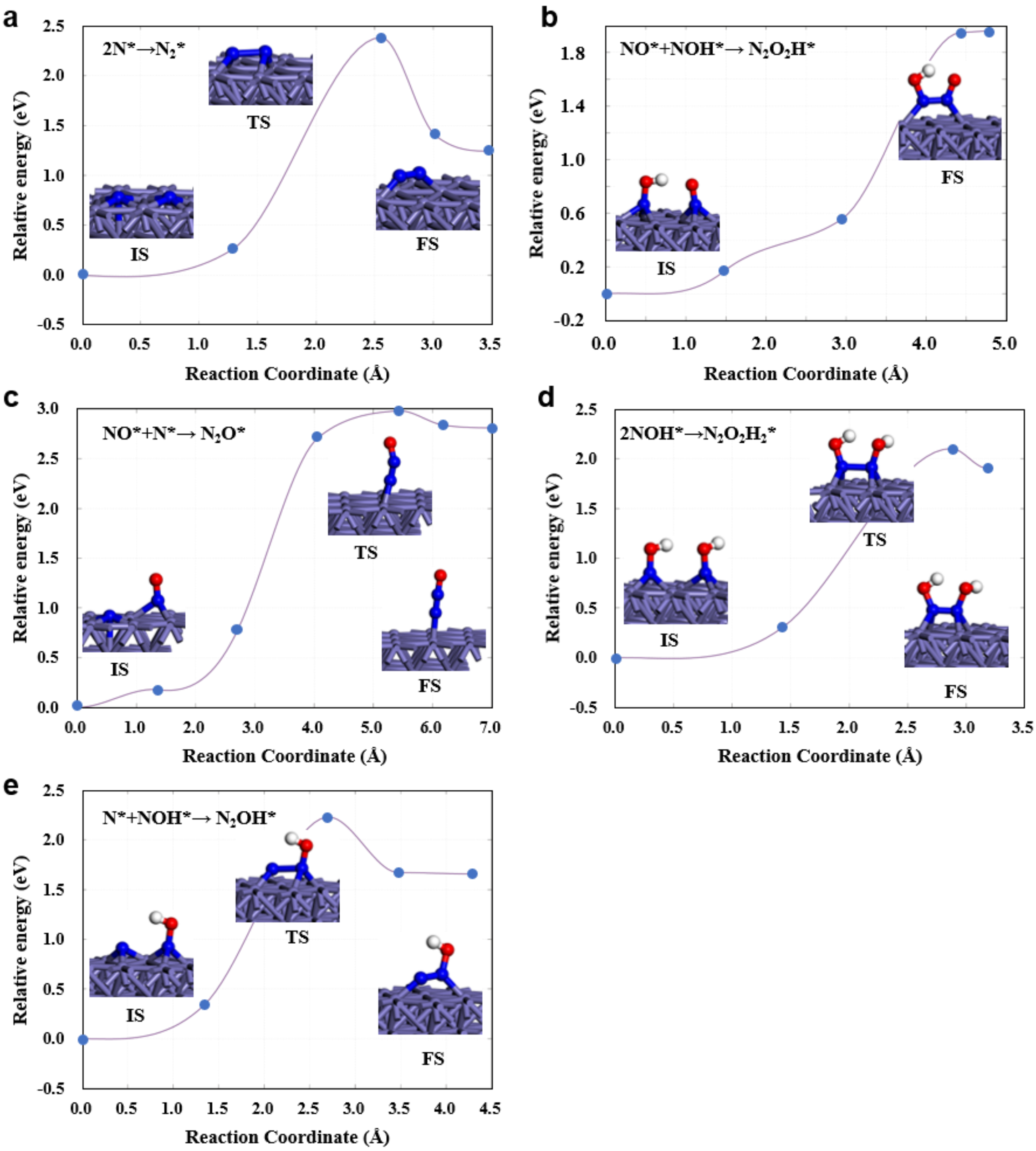

Figure S6. Energy profiles and corresponding structures of initial (TS), transition (TS), and final (FS) state for different N-N coupling reactions on pristine Fe. (a) $2N^* \rightarrow N_2^*$, (b) $NO^*+NOH^* \rightarrow N_2O_2H^*$, (c) $N^*+NO^* \rightarrow N_2O^*$, (d) $2NOH^* \rightarrow N_2O_2H_2^*$, (e) $N^*+NOH^* \rightarrow N_2OH^*$.

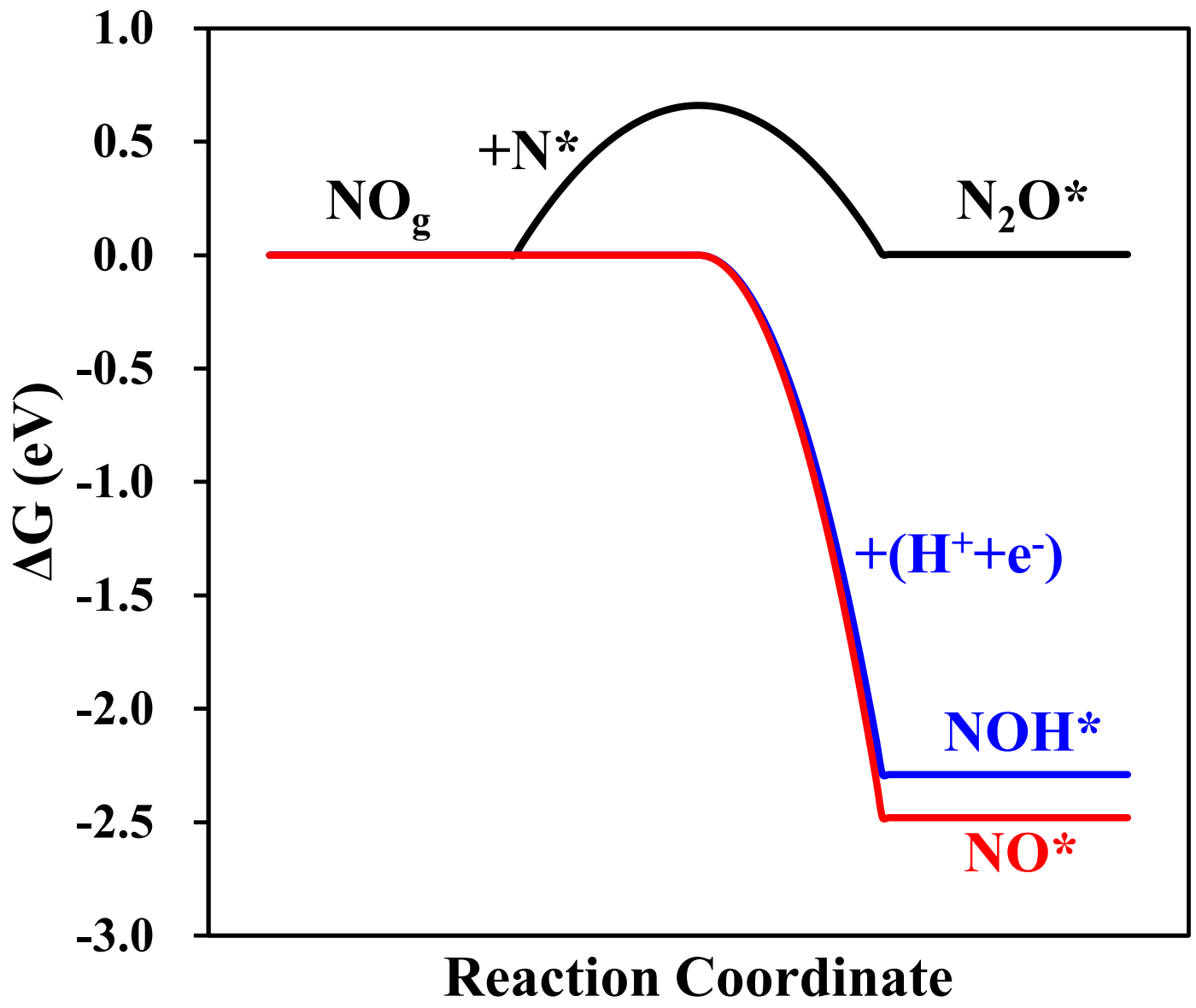

Figure S7. Comparison between NO_g coupling with N* (black), NO_g adsorption (red) and protonation associated adsorption (blue).

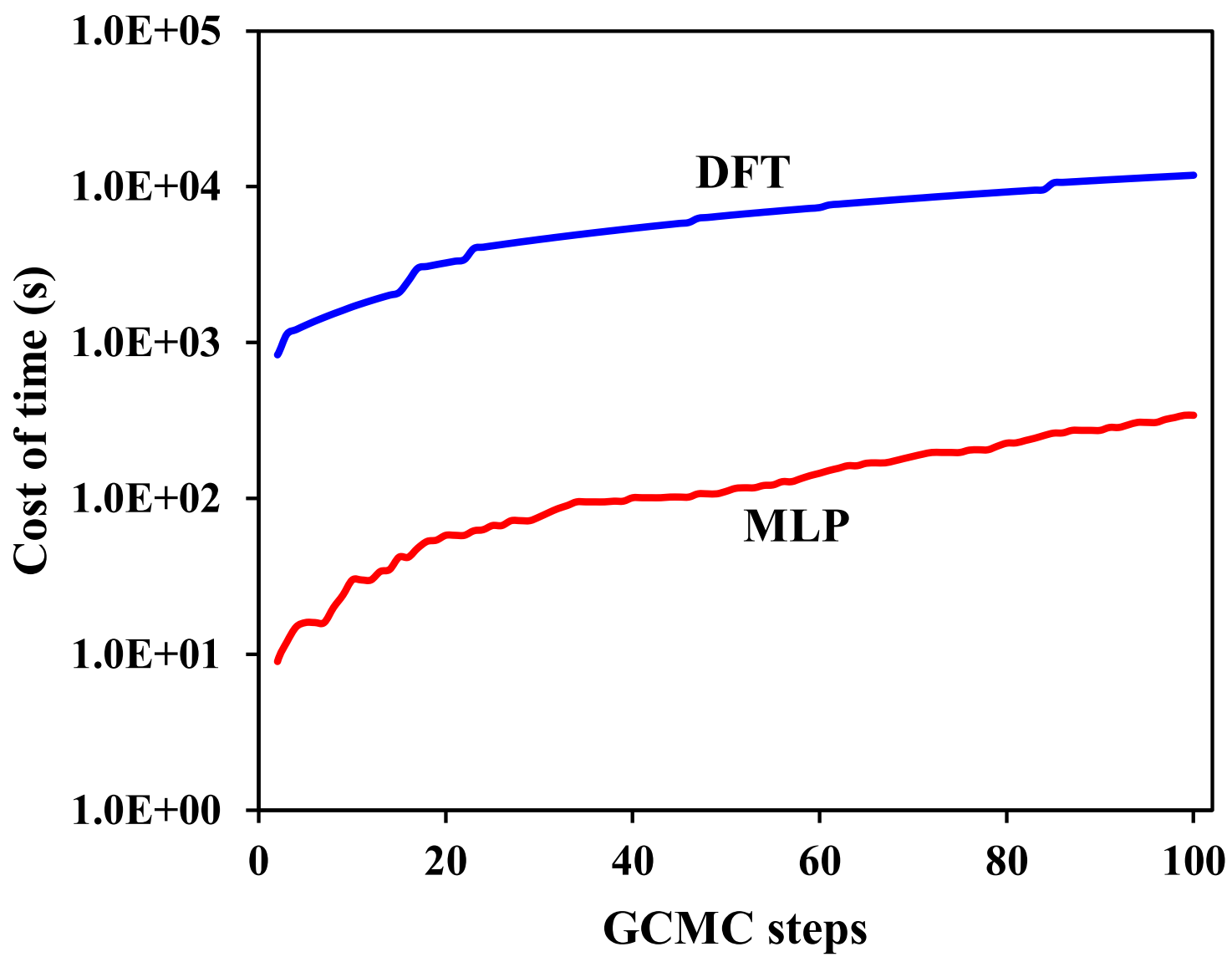

Figure S8. Comparison between the costs of time of the MLP and DFT-driven GCMC simulation.

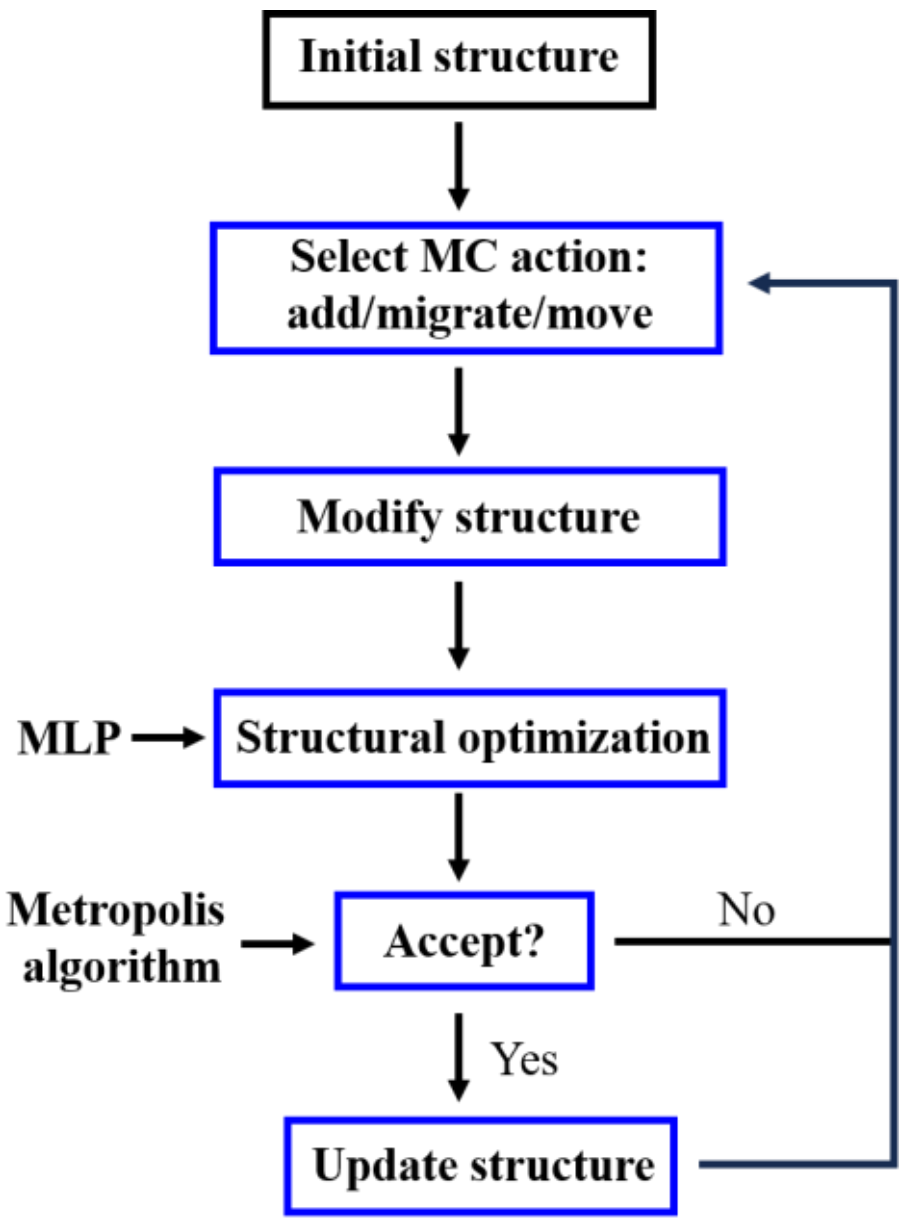

Figure S9. Flowchart of GCMC simulation.

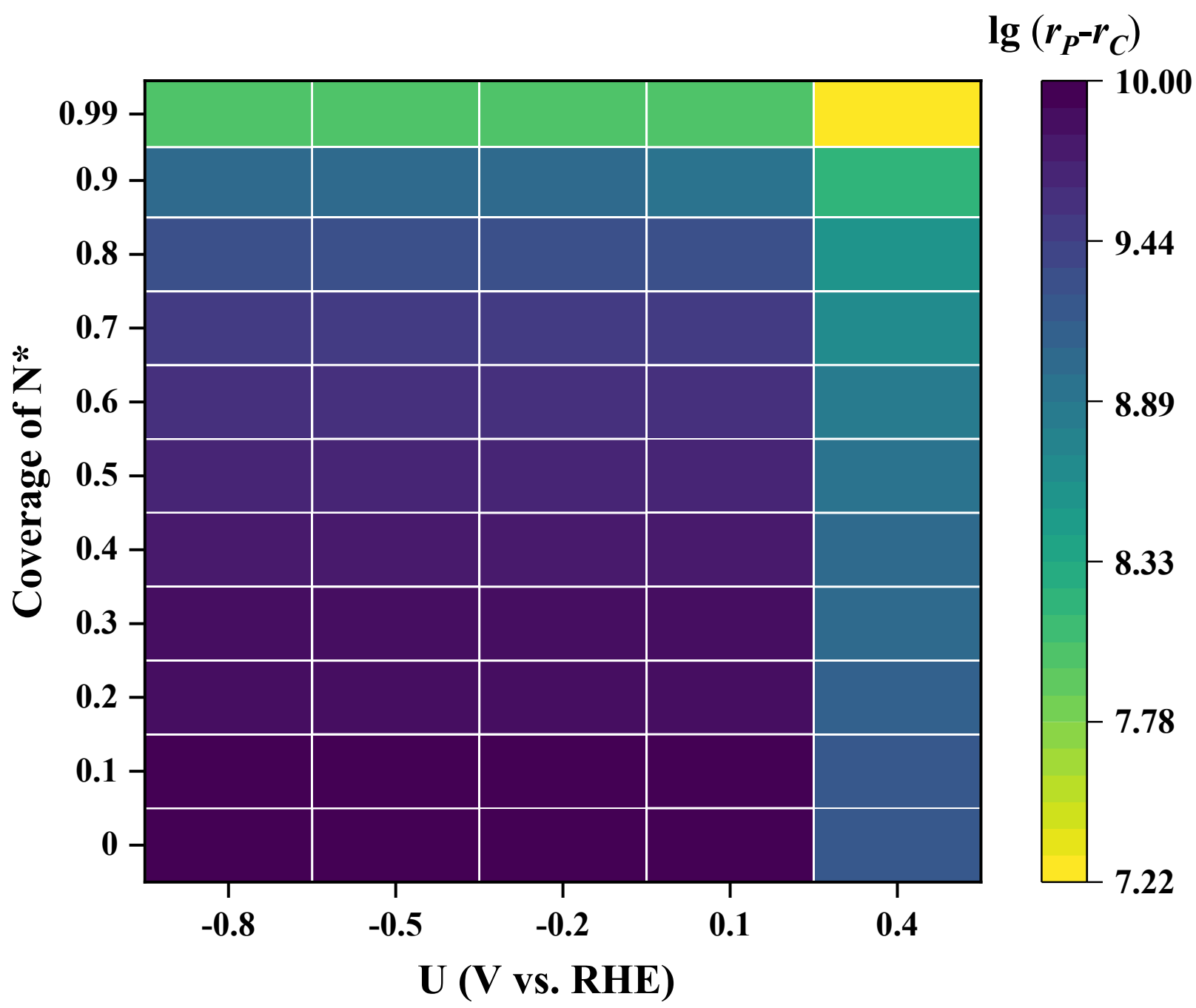

Figure S10. Difference between the production rate (r_P) and consumption rate (r_C) of N* on Fe (110) surface at different potentials and θ_{N*}.

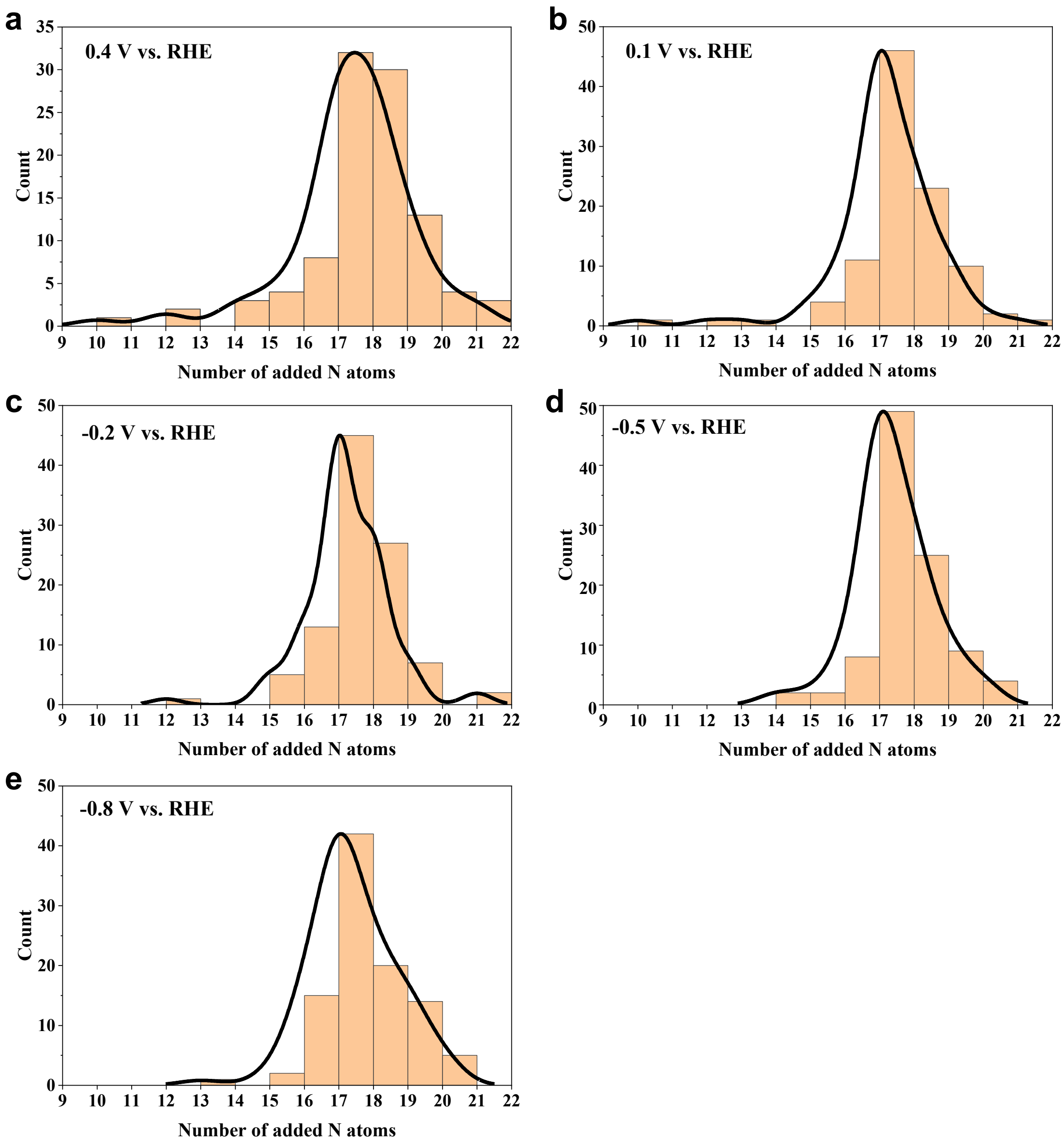

Figure S11. Distribution of the number of added N atoms from 100 independent GCMC simulation at different potentials.

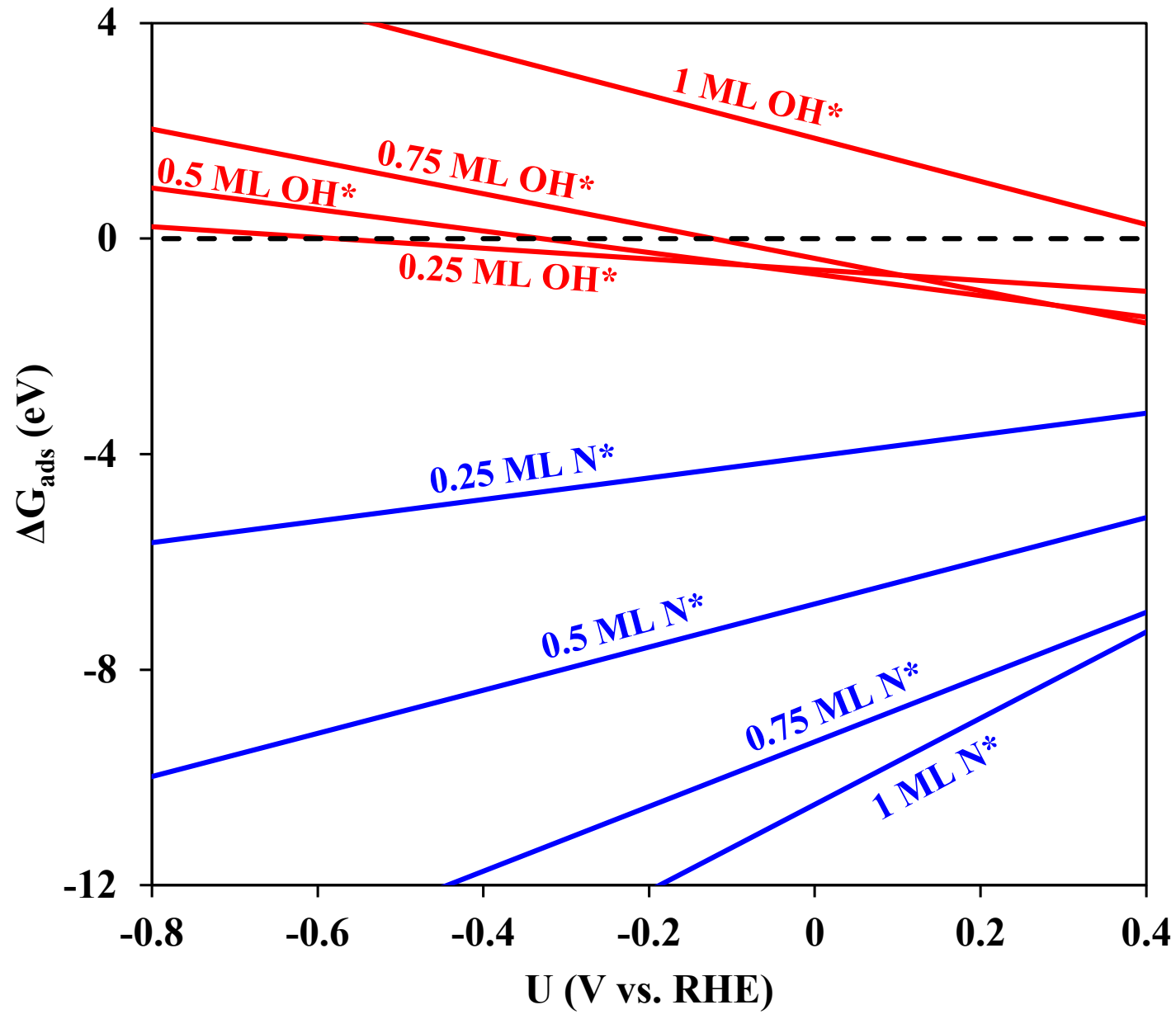

Figure S12. Adsorption free energies of N* and OH* on Fe (110) with reference to NO, H_2O, and $(H^+ + e^-)$, based on the reaction xNO + $2x$ $(H^+ + e^-)$ + x_* → xN$_*$ + $x$$H_2O$ and $x$$H_2O$ + x_* → xOH$_*$ + $x$$(H^+ + e^-)$, respectively.

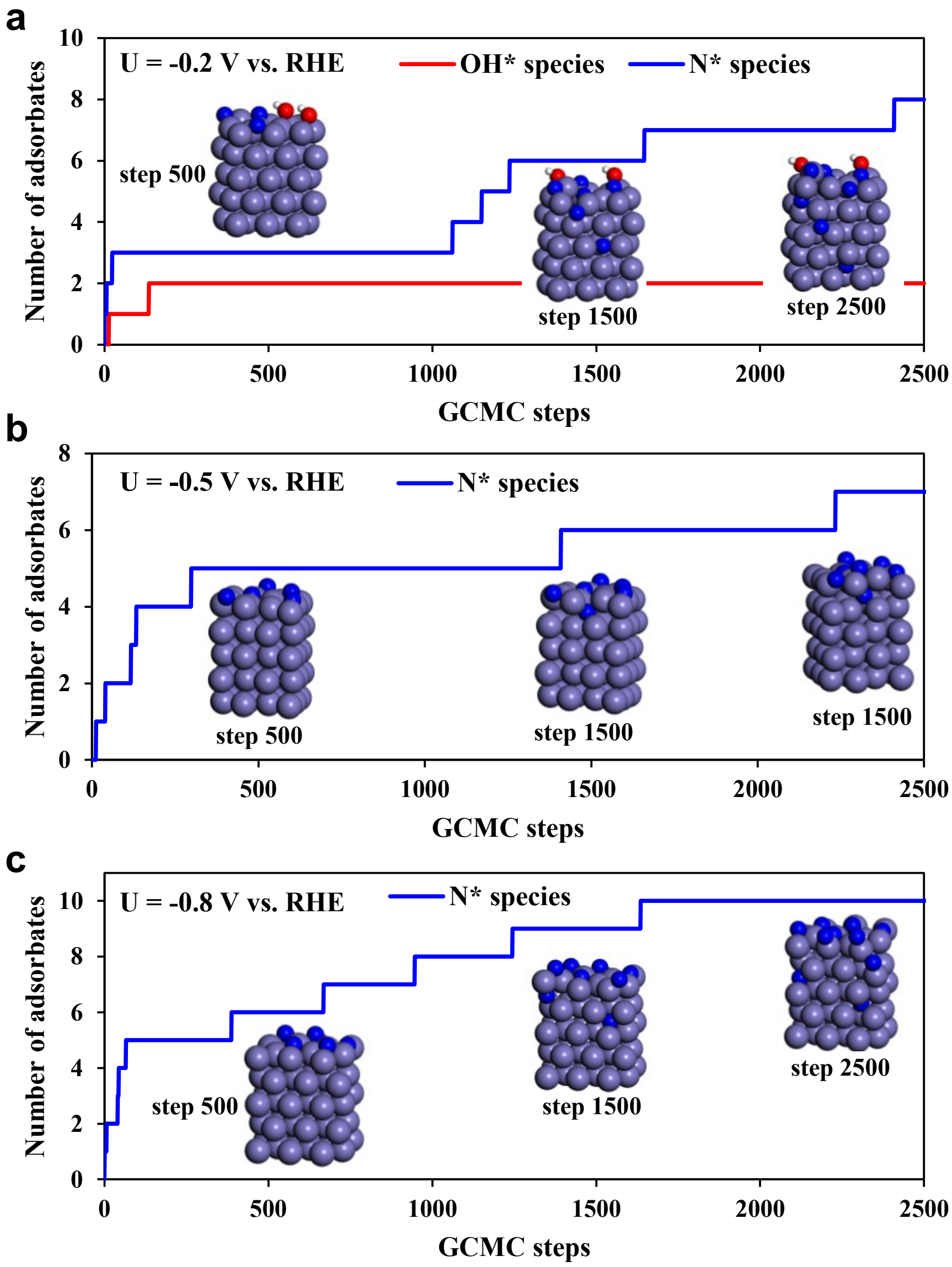

Figure S13. DFT-driving GCMC simulations for the competitive accumulations of OH* and N* on Fe at (a) -0.2, (b) -0.5, and (c) -0.8 V vs. RHE.

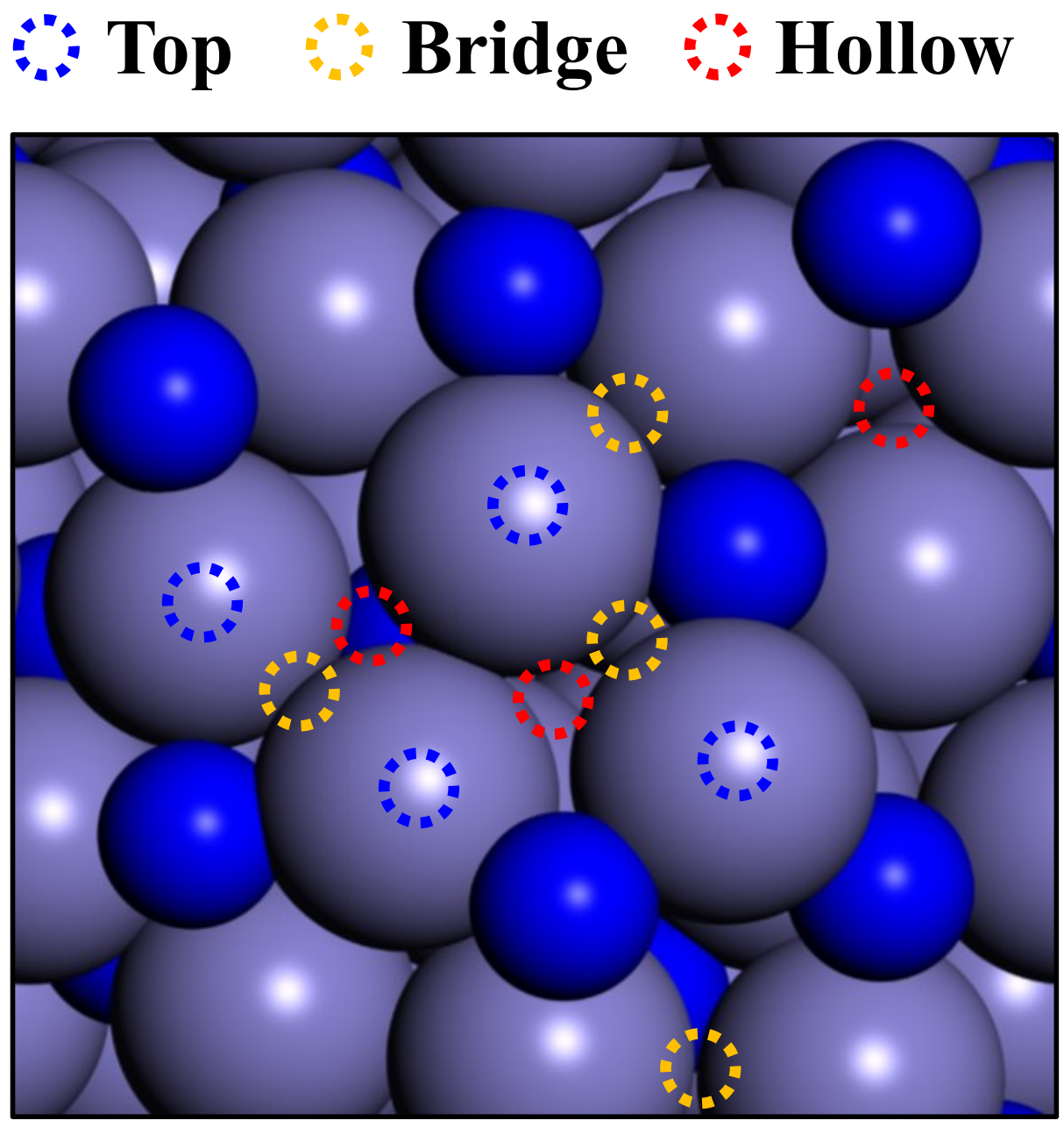

Figure S14. Classifications of reaction sites of FeN_x motifs.

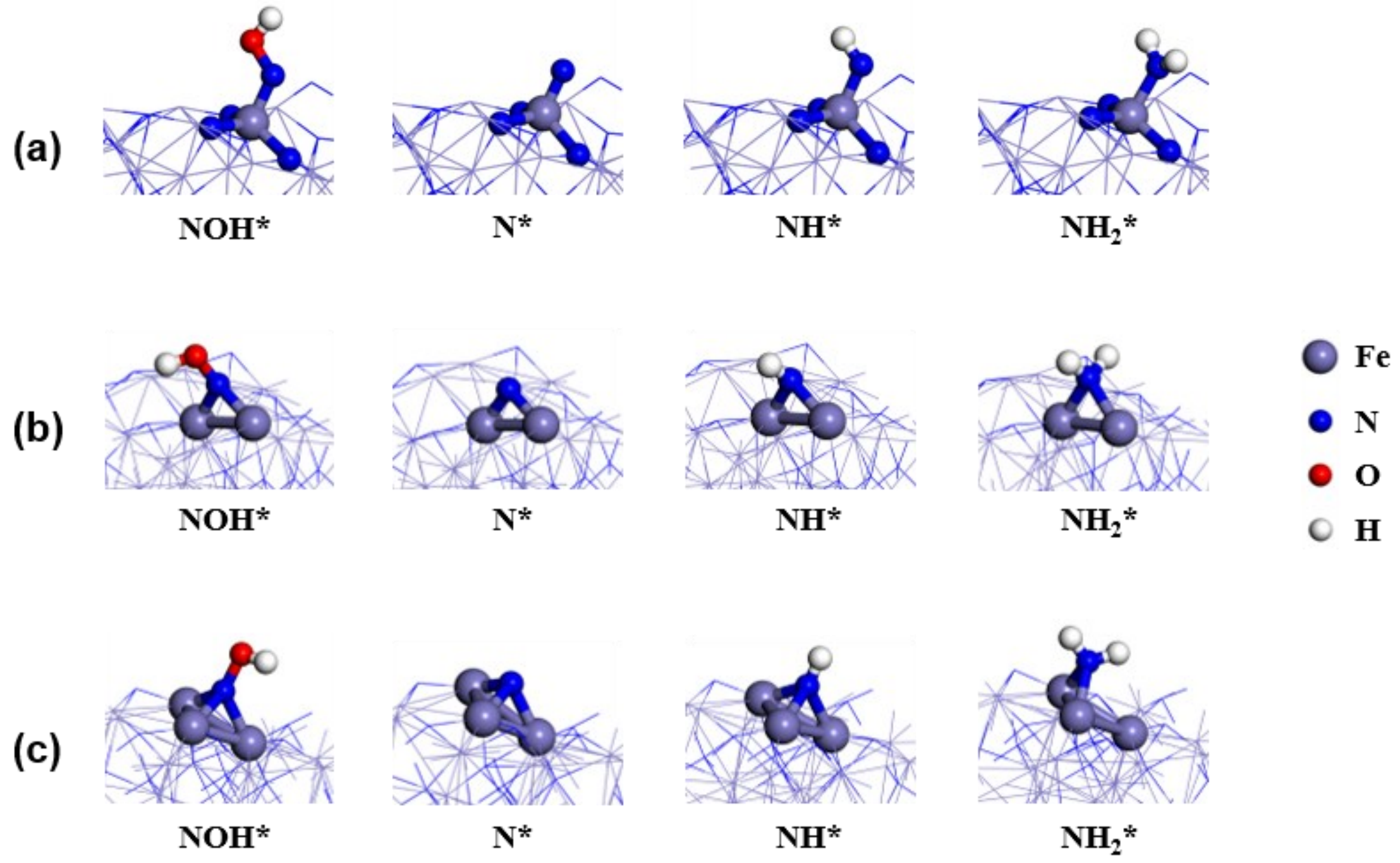

Figure S15. Examples of the adsorption structures of NOH*, N*, NH*, and NH_2* on (a) top, (b) bridge, and (c) hollow site. Note that the adsorption of NH_2* on hollow site by N coordinating with 3 Fe atoms is unstable, which will transfer to the bridge site spontaneously after structural optimization.

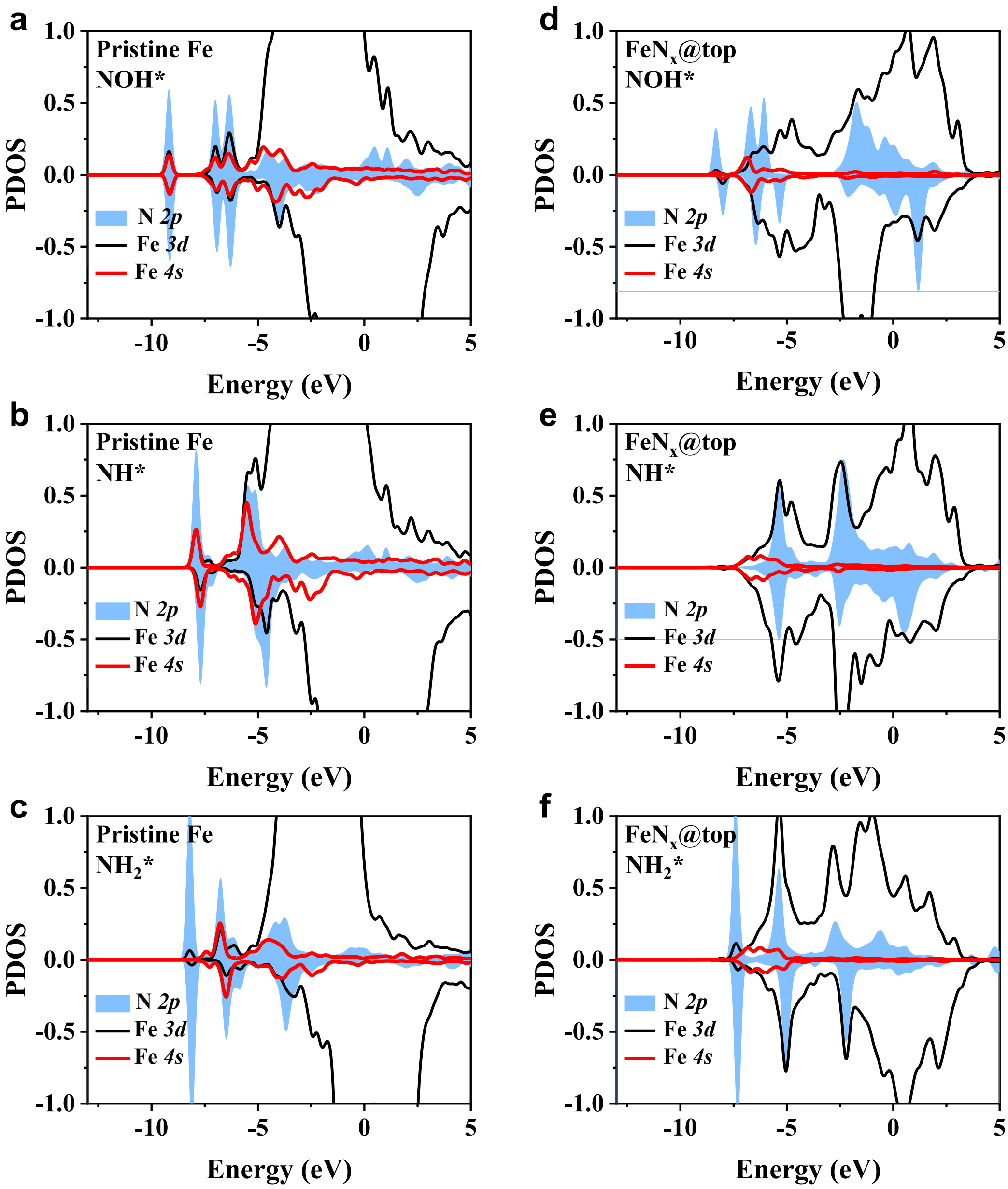

Figure S16. PDOS of Fe and adsorbed N-containing species on pristine Fe (a-c) and FeN_x@top (d-f).

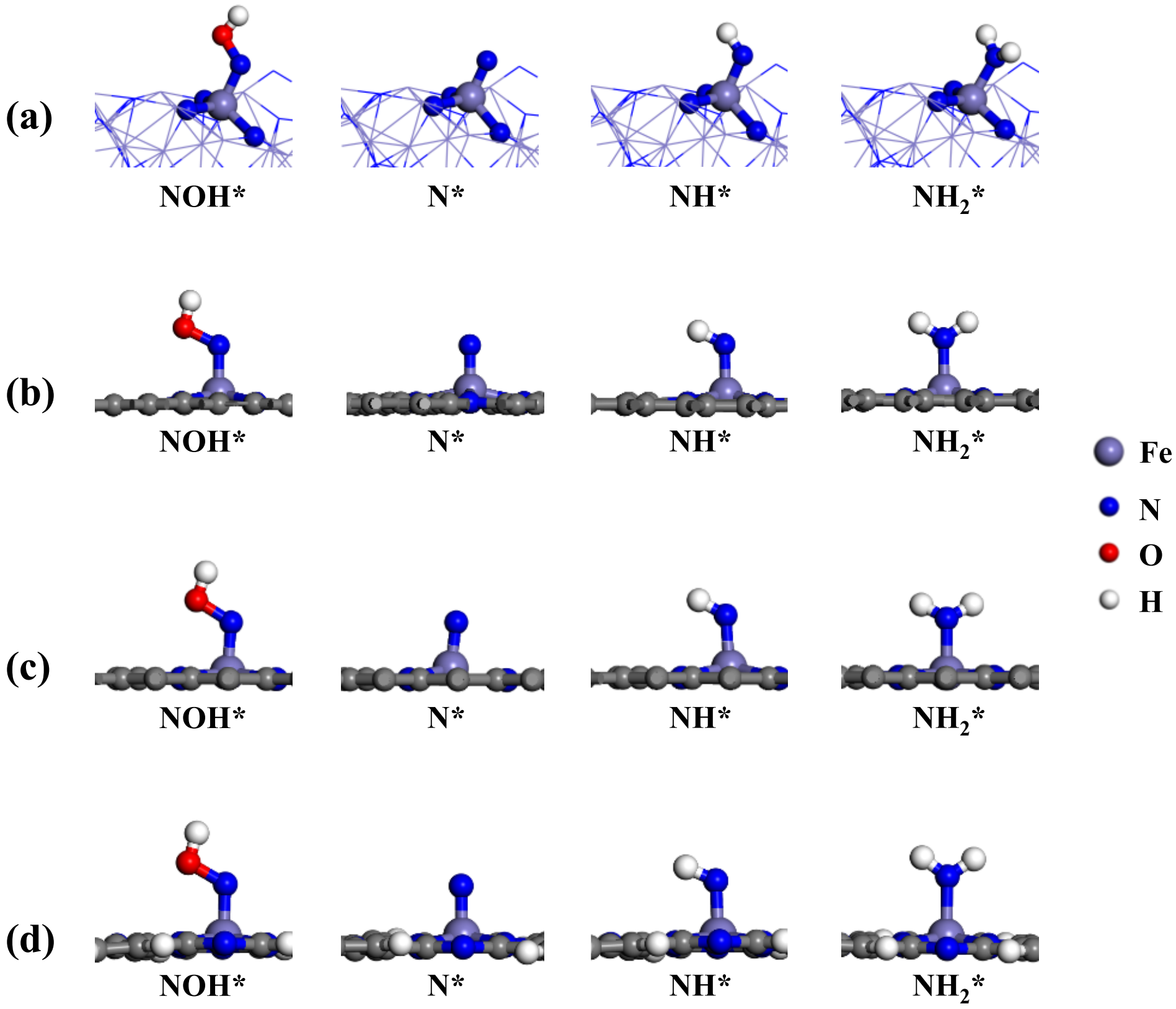

Figure S17. Adsorption configurations of different intermediates on FeN_x motifs (a), pyridinic-FeN_4 (b), pyrrolic-FeN_4 (c), and FePc (d).

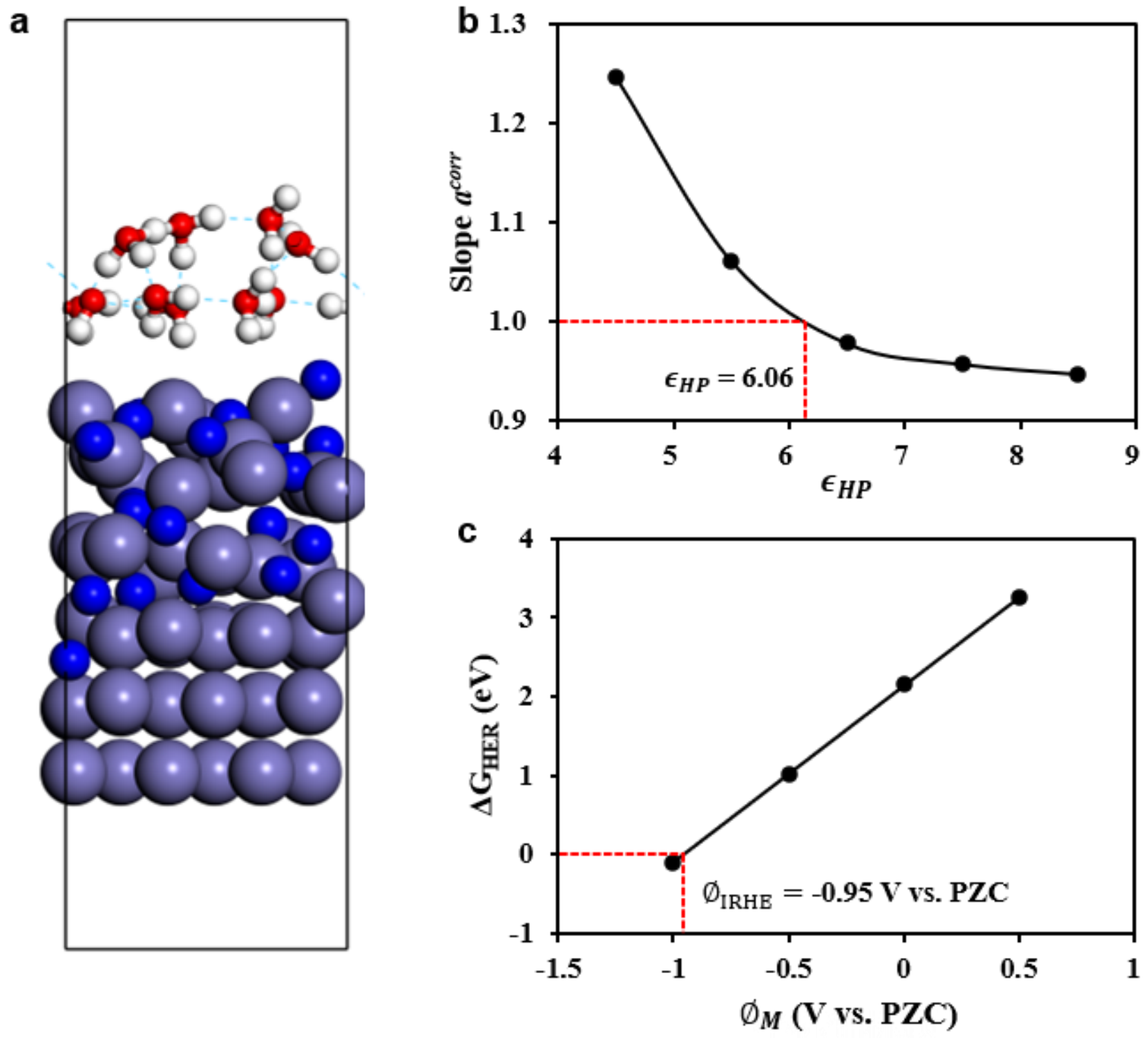

Figure S18. (a) Model of FeN_x motifs with water layer for electrochemical barriers calculation. (b) Fitting ϵ_{HP} of FeN_x motifs with water layer. (c) Fitting IRHE of FeN_x motifs with water layer.

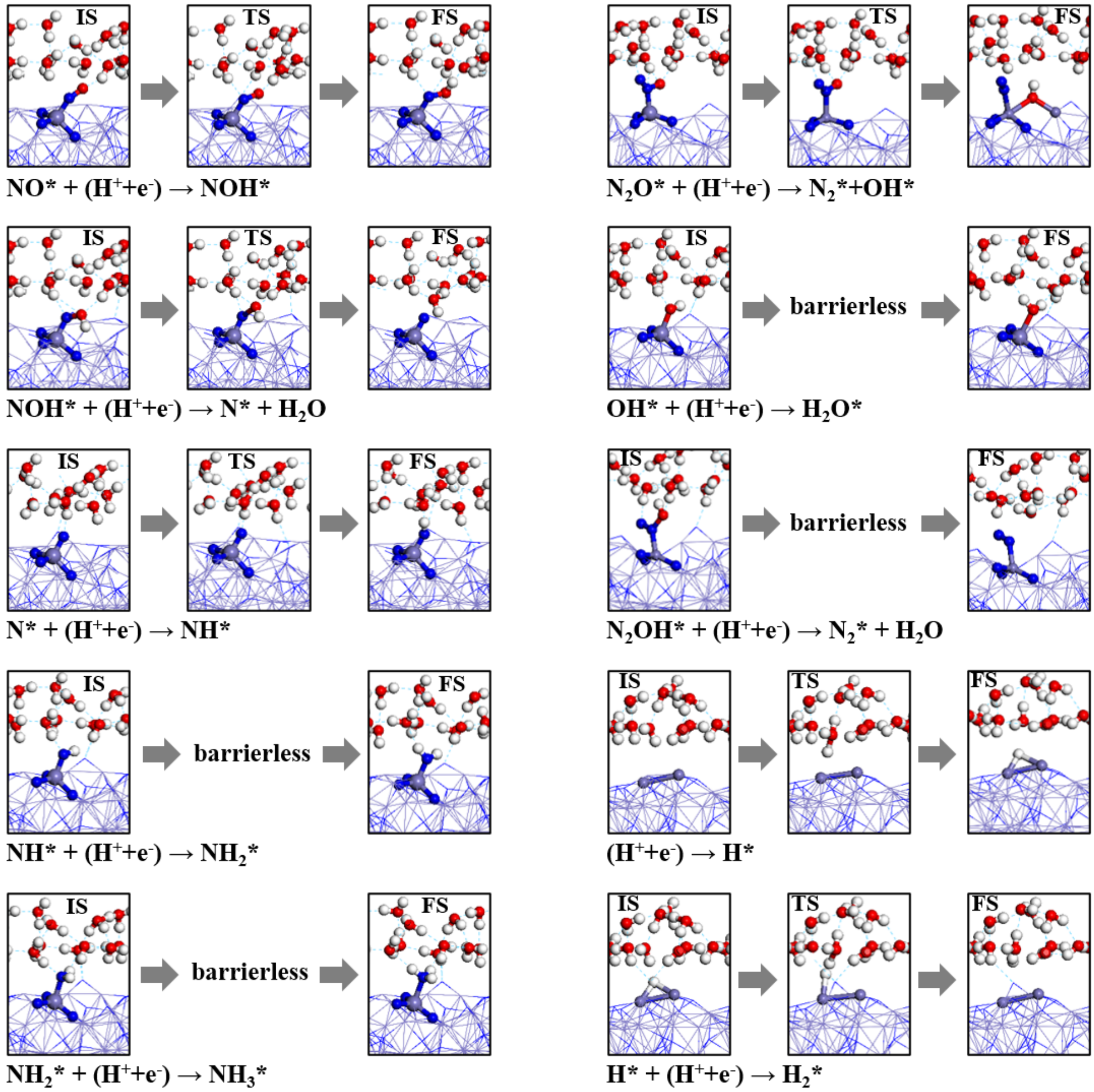

Figure S19. Atomic structures of the initial (IS), transition (TS), and final state (FS) for different electrochemical elementary steps for NH_3, N_2O, N_2, and H_2 production on FeN_x motifs.

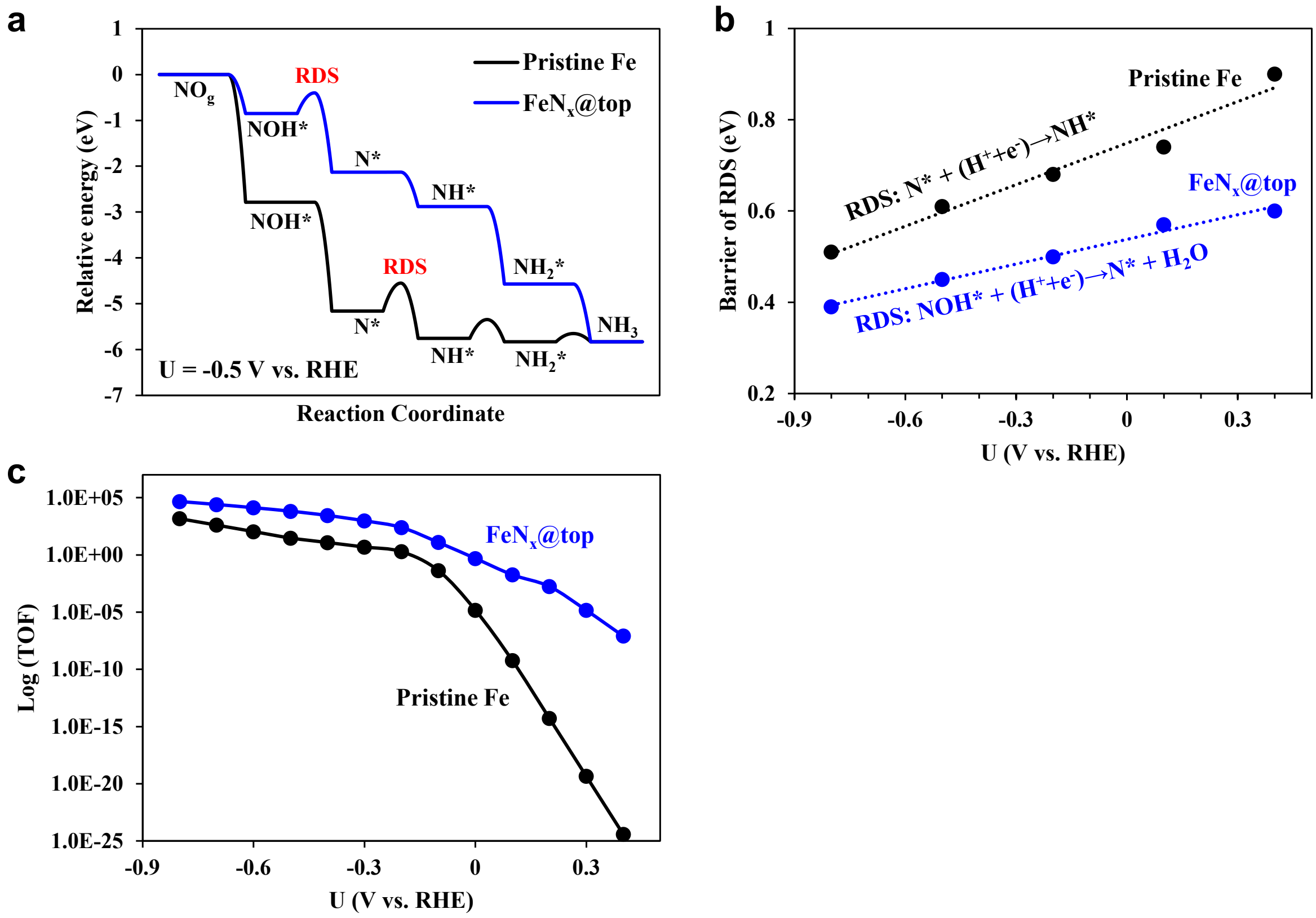

Figure S20. (a) Free energy diagrams of eNORR to NH_3 on pristine Fe and FeN_x@top at -0.5 V vs. RHE. (b) Energy barriers of RDS on pristine Fe and FeN_x@top at varying potentials. (c) TOF of eNORR to NH_3 on pristine Fe and FeN_x@top at varying applied potential.

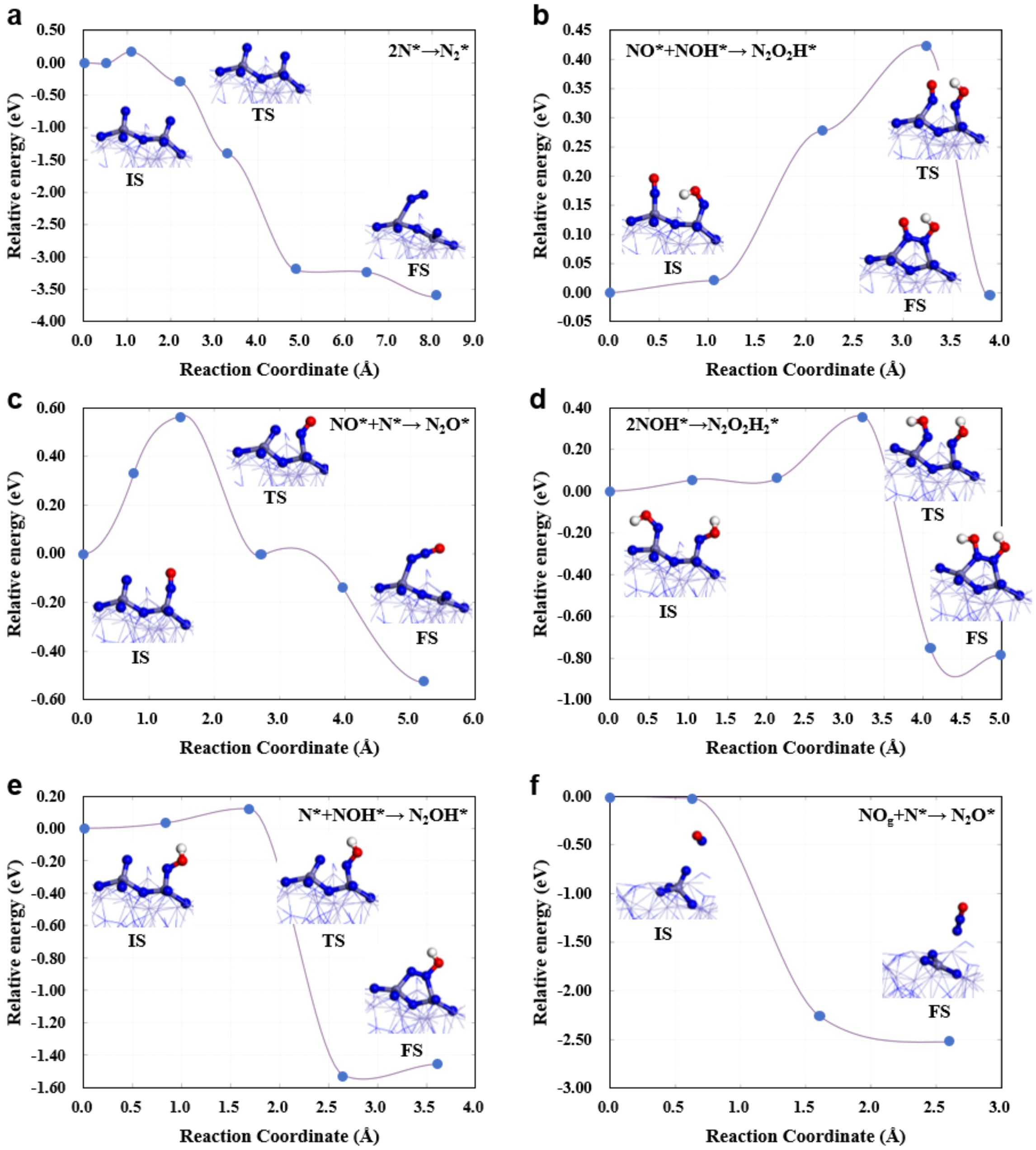

Figure S21. Energy profiles and corresponding structures of initial (TS), transition (TS), and final (FS) state for different N-N coupling reactions on FeN_x motifs. (a) $2N^*\rightarrow N_2^*$, (b) $NO^*+NOH^*\rightarrow N_2O_2H^*$, (c) $N^*+NO^*\rightarrow N_2O^*$, (d) $2NOH^*\rightarrow N_2O_2H_2^*$, (e) $N^*+NOH^*\rightarrow N_2OH^*$, (e) $NO_g+N^*\rightarrow N_2O^*$.

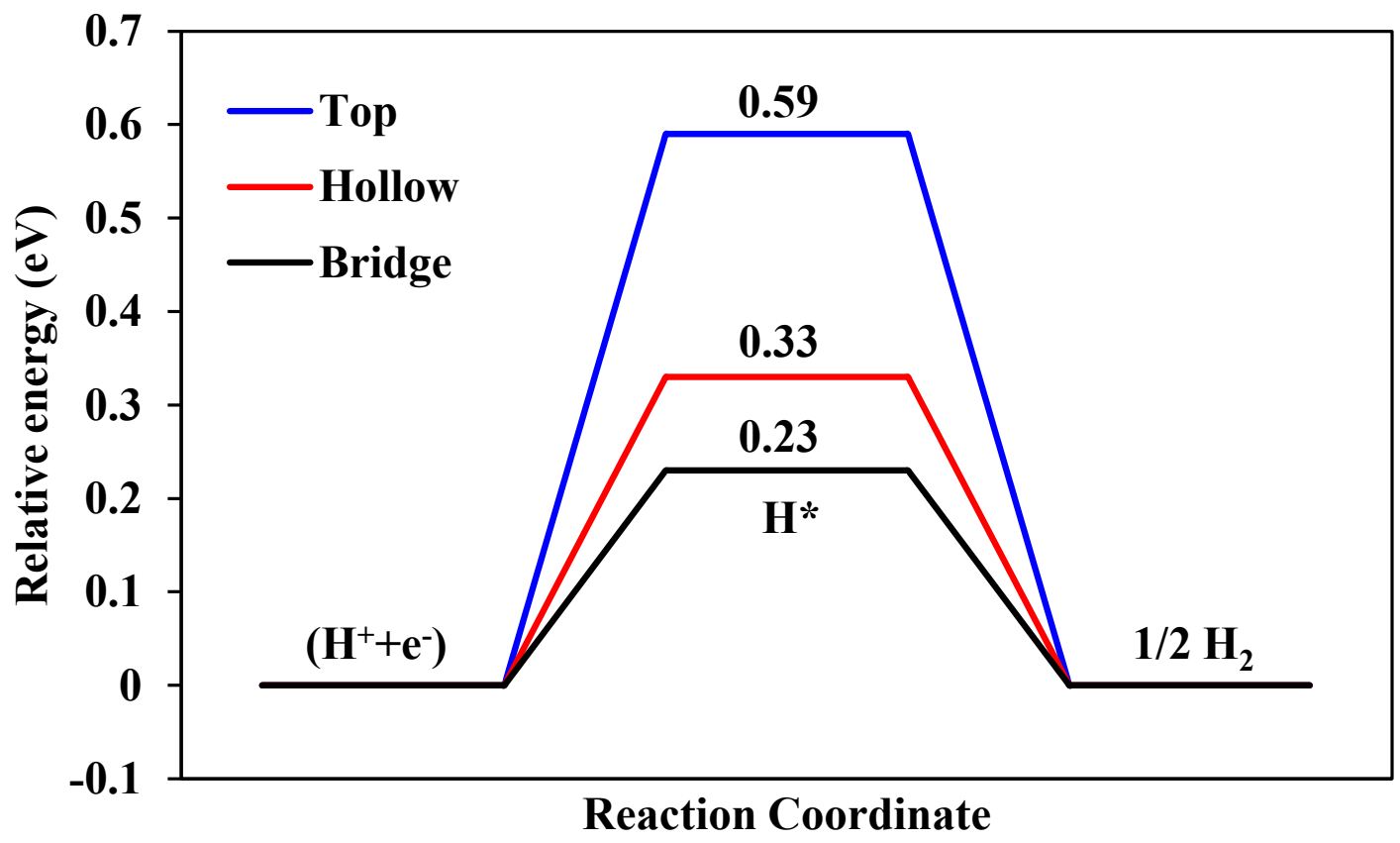

Figure S22. Free energy diagrams of HER on top, hollow, and bridge site of FeN_x motifs at 0V vs. RHE.

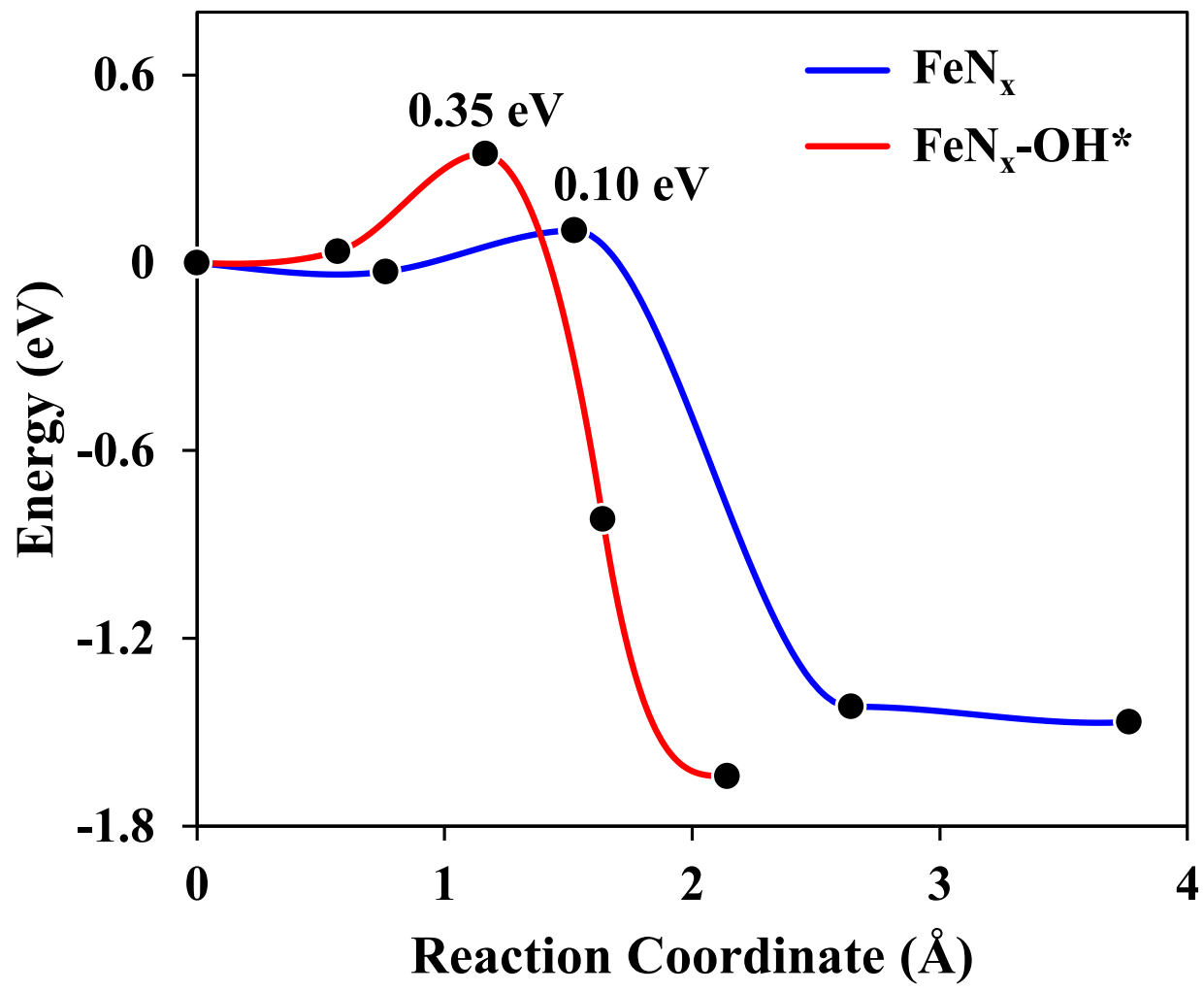

Figure S23. Energy barriers of the N*-NOH* coupling reaction (N*+NOH* → N_2OH*) on FeN_x motifs and FeN_x with an adsorbed OH* (FeN_x-OH*).

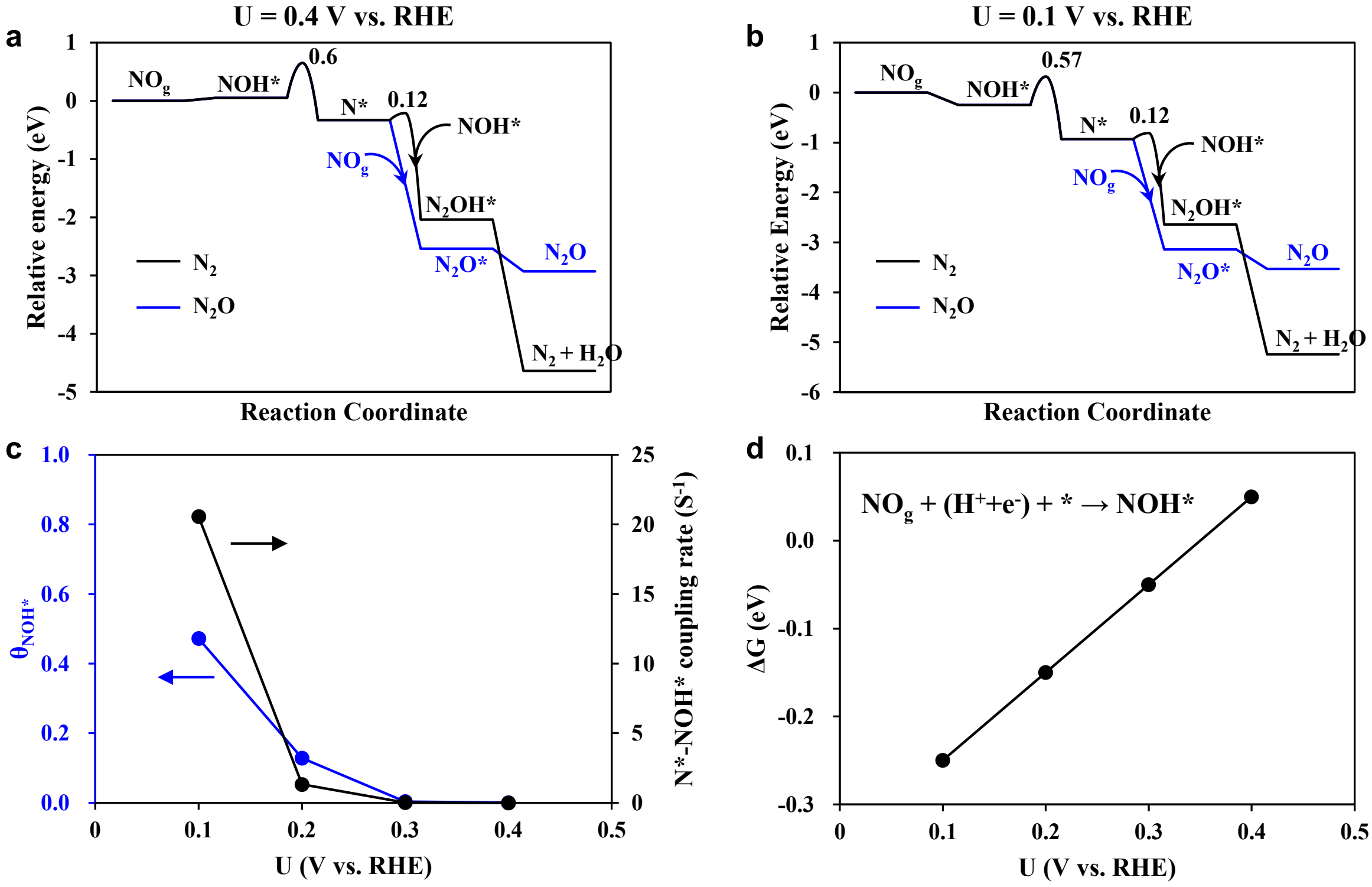

Figure S24. (a-b) Free energy diagrams for eNORR to N_2O and N_2 on FeN_x motifs at 0.4 and 0.1 V vs. RHE, respectively. (c) Variation of θ_{NOH*} (blue) and N*-NOH* coupling rate (black) with potential on FeN_x motifs. (d) Variation of NOH* formation energy with potential on FeN_x motifs.

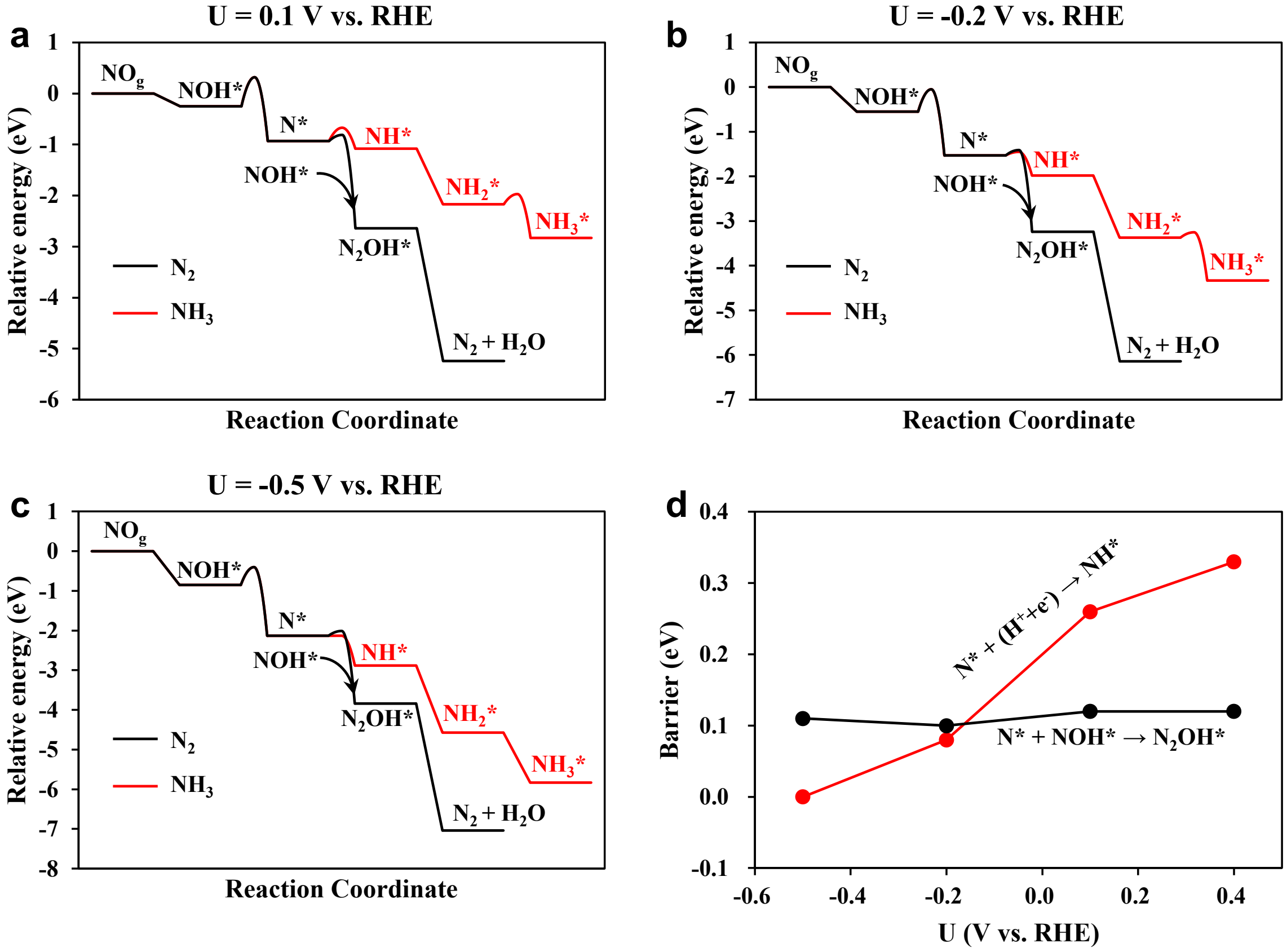

Figure S25. (a-c) Free energy diagrams for eNORR to N_2 and NH_3 on FeN_x motifs at 0.1, -0.2, and -0.5 V vs. RHE, respectively. (d) Variation of N* protonation barrier (red) and N*-NOH* coupling barrier (black) with applied potential.

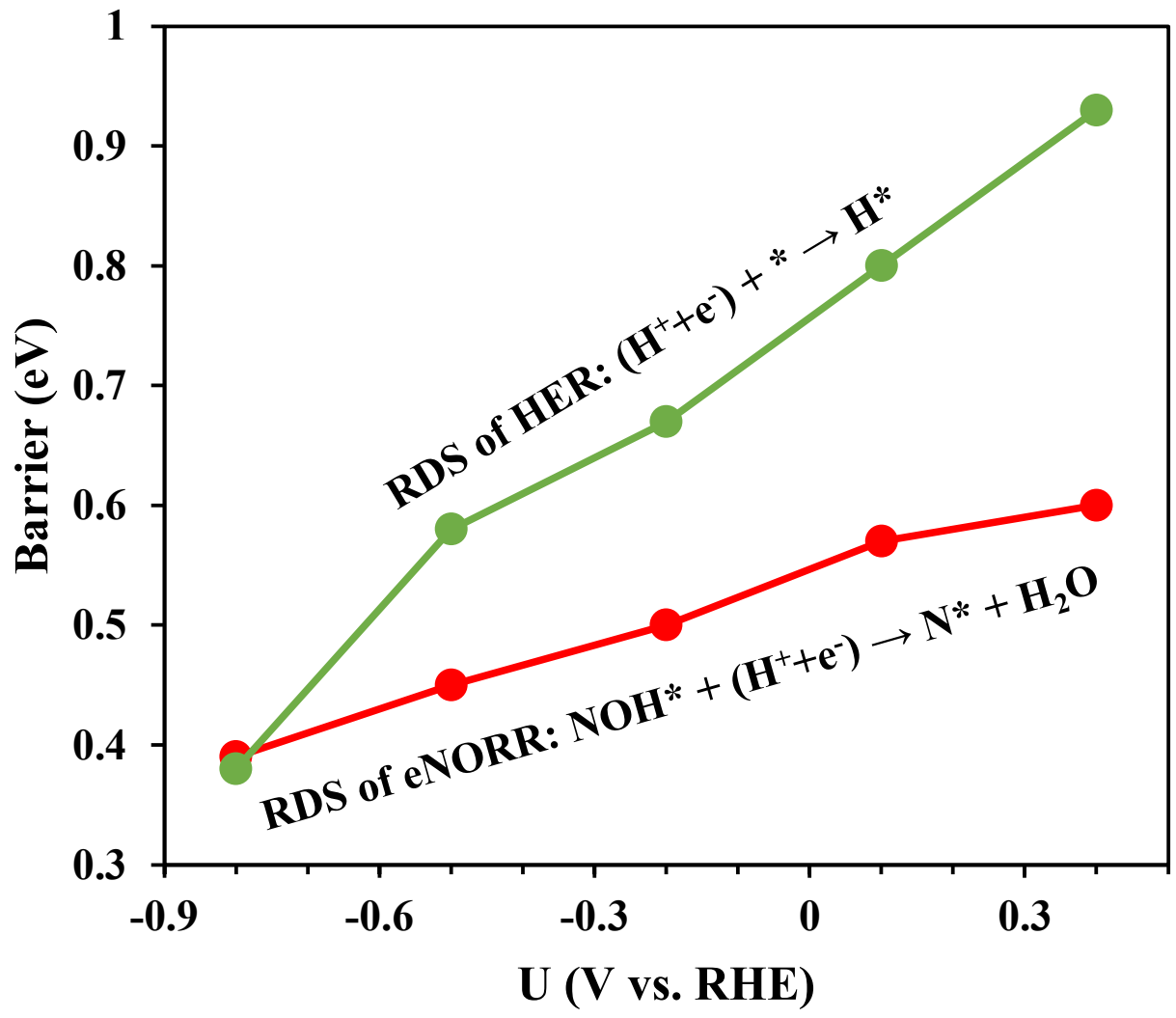

Figure S26. Variation of the energy barrier of Volmer reaction (green) and NOH* protonation (red) on FeN_x motifs with potential.

Table S1. Obtained reaction pathways for eNORR to NH_3 by CatRPD analysis

**Path 0**	$NO_g + (H^+ + e^-) + * \rightarrow NOH*$<br>$NOH* + (H^+ + e^-) \rightarrow N* + H_2O$<br>$N* + (H^+ + e^-) \rightarrow NH*$<br>$NH* + (H^+ + e^-) \rightarrow NH_2*$<br>$NH_2* + (H^+ + e^-) \rightarrow NH_3 + *$
**Path 1**	$NO_g + (H^+ + e^-) + * \rightarrow HNO*$<br>$HNO* + (H^+ + e^-) \rightarrow H_2NO*$<br>$H_2NO* + (H^+ + e^-) \rightarrow H_2NOH*$<br>$H_2NOH* + (H^+ + e^-) \rightarrow NH_2* + H_2O$<br>$NH_2* + (H^+ + e^-) \rightarrow NH_3 + *$
**Path 2**	$NO_g + (H^+ + e^-) + * \rightarrow HNO*$<br>$HNO* + (H^+ + e^-) \rightarrow HNOH*$<br>$HNOH* + (H^+ + e^-) \rightarrow NH* + H_2O$<br>$NH* + (H^+ + e^-) \rightarrow NH_2*$<br>$NH_2* + (H^+ + e^-) \rightarrow NH_3 + *$
**Path 3**	$NO_g + (H^+ + e^-) + * \rightarrow HNO*$<br>$HNO* + (H^+ + e^-) \rightarrow HNOH*$<br>$HNOH* + (H^+ + e^-) \rightarrow H_2NOH*$<br>$H_2NOH* + (H^+ + e^-) \rightarrow NH_2* + H_2O$<br>$NH_2* + (H^+ + e^-) \rightarrow NH_3 + *$
**Path 4**	$NO_g + (H^+ + e^-) + * \rightarrow NOH*$<br>$NOH* + (H^+ + e^-) \rightarrow HNOH*$<br>$HNOH* + (H^+ + e^-) \rightarrow NH* + H_2O$<br>$NH* + (H^+ + e^-) \rightarrow NH_2*$<br>$NH_2* + (H^+ + e^-) \rightarrow NH_3 + *$
**Path 5**	$NO_g + (H^+ + e^-) + * \rightarrow NOH*$<br>$NOH* + (H^+ + e^-) \rightarrow HNOH*$<br>$HNOH* + (H^+ + e^-) \rightarrow H_2NOH*$<br>$H_2NOH* + (H^+ + e^-) \rightarrow NH_2* + H_2O$<br>$NH_2* + (H^+ + e^-) \rightarrow NH_3 + *$

Table S2. Considered elementary steps of eNORR to NH_3 and corresponding barriers and reaction free energies for microkinetic modelling on pristine Fe at different potentials

Elementary steps	Barrier (eV)					Reaction free energy (eV)				
	-0.8 V	-0.5 V	-0.2 V	0.1 V	0.4 V	-0.8 V	-0.5 V	-0.2 V	0.1 V	0.4 V
$NO_g + (H^+ + e^-) + * \rightarrow NOH*$	0.00	0.00	0.00	0.00	0.00	-3.09	-2.79	-2.49	-2.19	-1.89
$NOH* + (H^+ + e^-) \rightarrow N* + H_2O$	0.00	0.00	0.00	0.05	0.16	-2.68	-2.38	-2.08	-1.78	-1.48
$N* + (H^+ + e^-) \rightarrow NH*$	0.51	0.61	0.68	0.74	0.90	-0.90	-0.60	-0.30	0.01	0.31
$NH* + (H^+ + e^-) \rightarrow NH_2*$	0.35	0.41	0.45	0.50	0.80	-0.38	-0.08	0.23	0.53	0.83
$NH_2* + (H^+ + e^-) \rightarrow NH_3 + *$	0.13	0.18	0.20	0.29	0.30	-0.33	-0.03	0.28	0.58	0.88

Table S3. Structures used to construct the dataset of MLP

Structures	AIMD temperature (K)	Training set	Test set
Fe_{36}	1500	320	80
$Fe_{36}O_3$	1500	320	80
$Fe_{36}N_2O_4$	1500	320	80
$Fe_{36}N_1$	1500	320	80
$Fe_{36}N_3O_1$	1500	320	80
$Fe_{36}N_2$	1500	320	80
$Fe_{36}N_2O_3$	1500	320	80
$Fe_{36}N_4O_5$	1500	320	80
$Fe_{36}O_1$	1500	320	80
$Fe_{36}N_3O_1$	1500	320	80
$Fe_{36}N_4O_4$	1500	320	80
$Fe_{36}N_5$	1500	320	80
$Fe_{36}N_6$	1500	320	80
$Fe_{36}N_3O_3$	1500	320	80
$Fe_{36}N_4O_6$	1500	320	80
$Fe_{36}N_4O_1$	1500	320	80
$Fe_{36}N_7O_6$	1500	320	80
$Fe_{36}O_6$	1500	320	80
$Fe_{36}N_2O_9$	1500	320	80
$Fe_{36}N_8O_5$	1500	320	80
$Fe_{36}N_3O_3$	1500	320	80
$Fe_{36}N_6O_6$	1500	320	80
$Fe_{36}N_3O_5$	500	320	80
$Fe_{36}N_4O_6$	500	320	80
$Fe_{36}N_2O_3$	500	320	80
$Fe_{36}N_5O_5$	500	320	80
$Fe_{36}N_5O_3$	500	320	80
$Fe_{36}N_3O_1$	500	320	80
$Fe_{36}N_4O_1$	500	320	80
$Fe_{36}N_2O_1$	500	320	80
$Fe_{36}N_1O_3$	500	320	80
$Fe_{36}N_5O_4$	500	320	80
$Fe_{36}N_5O_4$	500	320	80
$Fe_{36}N_3O_5$	1500	320	80
$Fe_{36}N_2O_3$	1500	320	80
$Fe_{36}N_4O_1$	1500	320	80
$Fe_{36}N_1O_3$	1500	320	80
$Fe_{36}N_5O_4$	1500	320	80

Table S3 (continued). Structures used to construct the dataset of MLP

**Structures**	**AIMD temperature (K)**	**Training set**	**Test set**
$Fe_{64}N_2O_6$	500	320	80
$Fe_{64}N_2O_2$	500	320	80
$Fe_{64}N_2O_6$	1500	320	80
$Fe_{64}N_2O_2$	1500	320	80
$Fe_{80}N_4O_5$	500	320	80
$Fe_{80}N_3O_6$	500	320	80
$Fe_{80}N_4O_5$	1500	320	80
Random structures	/	159	44

Table S4. Adsorption free energies of NOH*, N*, NH*, NH_2* on 100 different top, bridge, and hollow sites

Site number	G_{ad}(NOH*) (eV)			G_{ad}(N*) (eV)			G_{ad}(NH*) (eV)			G_{ad}(NH_2*) (eV)		
	Top	Bridge	Hollow	Top	Bridge	Hollow	Top	Bridge	Hollow	Top	Bridge	Hollow
1	-0.71	-1.24	-1.47	-1.59	-2.64	-2.65	-1.94	-2.89	-3.22	-2.78	-3.8	-3.74
2	-1.32	-1.97	-0.77	-2.14	-3.09	-1.56	-2.37	-3.5	-2.48	-3.28	-4.46	-3.06
3	-1.31	-0.66	-1.68	-1.94	-1.69	-3.11	-2.61	-2.49	-3.44	-3.17	-3.56	-3.95
4	-0.87	0.17	-1.03	-1.85	-0.6	-2.27	-2.23	-1.51	-2.93	-3.26	-2.88	-3.38
5	-0.4	-1.4	-2.34	-1.14	-2.83	-4.01	-1.66	-3.65	-4.53	-2.76	-4.28	-5.03
6	-0.59	-1.67	-2.15	-1.54	-3.47	-3.51	-1.77	-3.93	-4	-2.9	-4.15	-4.21
7	-0.61	-0.53	-1.55	-1.59	-0.9	-3.3	-1.55	-1.56	-3.48	-2.9	-3.08	-4.56
8	-0.35	-0.8	-0.92	-1.13	-2.42	-3.27	-1.38	-2.78	-3.51	-2.57	-3.49	-2.85
9	-0.4	0.79	-2.22	-1.6	-1.55	-4.12	-1.7	-2.2	-4.15	-2.71	-2.95	-5.13
10	-0.67	0.41	-0.66	-1.42	-1.05	-2.35	-1.76	-1.69	-2.74	-2.75	-2.75	-3.86
11	-0.39	-0.59	-1.49	-1.22	-1.43	-2.51	-1.45	-1.94	-3.28	-2.82	-3.2	-3.11
12	-0.23	-0.58	-0.07	-1.39	-1.69	-1.15	-1.48	-2.2	-1.77	-2.51	-3.08	-3.24
13	-0.24	-0.17	-1.44	-1.3	-1.39	-3.27	-1.39	-2.03	-3.38	-2.58	-3.08	-3.34
14	-0.89	-0.54	0.28	-1.81	-1.78	-1.47	-2.2	-2.23	-1.37	-3.64	-2.97	-2.78
15	-0.14	-0.81	-0.74	-2.29	-1.4	-1.68	-2.54	-2.06	-2.45	-2.54	-2.97	-3.45
16	-0.34	-1.3	-0.01	-0.94	-3.24	-1.7	-1.62	-3.68	-2	-2.66	-4.34	-3.14
17	-0.33	-1.36	0.17	-1.07	-2.91	-1.33	-1.4	-3.22	-1.76	-2.64	-3.94	-3.68
18	-0.48	-0.72	-0.92	-1.01	-1.9	-2.72	-1.63	-2.3	-2.87	-2.81	-3.27	-3.07
19	0.37	-1.5	-0.99	-0.96	-3.01	-2.06	-0.64	-3.29	-2.49	-2.18	-4	-3.28
20	-0.83	-1.82	-0.73	-1.75	-3.07	-2.55	-2.22	-3.47	-2.75	-3.2	-4.03	-3.44
21	-0.52	-1.32	-2.87	-1.52	-3.14	-4.16	-1.57	-3.61	-4.61	-2.68	-4.5	-5.03
22	-0.16	-1.26	-0.81	-1.03	-2.74	-2.42	-1.81	-3.2	-2.66	-3.18	-4.03	-3.03
23	-0.1	-1.77	-1.65	-0.93	-3.27	-3.22	-1.15	-3.54	-3.6	-2.43	-4.23	-4.16
24	-0.41	-1.02	-1.57	-1.32	-2.35	-3.03	-1.67	-2.71	-3.55	-2.98	-3.59	-3.67
25	-0.22	-1.33	-1.72	-1.04	-3.54	-4.85	-1.3	-3.62	-5.34	-2.83	-3.69	-5.72
26	-0.56	-1.85	-1.37	-1.23	-3.35	-2.74	-1.67	-3.48	-3.64	-2.78	-4.19	-3.04
27	-0.31	-0.45	-1.36	-1.23	-2.74	-2.63	-1.74	-3.45	-3.36	-2.62	-3.79	-4.03
28	-0.86	-1.55	-1.07	-1.67	-2.68	-2.51	-2	-3.26	-2.84	-3.06	-4.15	-3.78
29	-0.99	-1.32	-1.3	-1.84	-3.61	-2.63	-2.3	-3.67	-3.07	-3.25	-4.1	-3.48
30	-0.68	-2.79	-1.49	-1.58	-4.07	-3.51	-1.95	-4.49	-3.72	-2.82	-5.09	-3.03
31	-0.46	-1.83	-1.58	-1.64	-3.06	-3.41	-1.86	-3.54	-3.53	-2.78	-4.04	-4.05
32	-0.77	-1.22	-1.64	-1.75	-2.39	-2.98	-2.93	-2.78	-3.36	-3.28	-3.77	-4.17
33	-1.42	-1.23	-2	-2.35	-2.42	-3.4	-2.43	-2.91	-3.7	-3.34	-3.6	-4.16
34	-0.8	-0.58	-1.99	-1.76	-1.74	-3.41	-1.92	-2.17	-3.81	-2.97	-3.2	-4.19
35	-0.72	-0.61	-0.7	-1.62	-1.33	-3.53	-2.01	-2.55	-2.45	-3.13	-3.69	-3.77
36	-0.88	-0.77	-1.2	-1.7	-3.12	-2.37	-2.07	-2.89	-3.03	-3.28	-3.34	-3.64
37	-0.08	-0.78	-3.32	-1.19	-1.96	-4.94	-1.34	-2.14	-5.31	-2.42	-3.5	-5.65
38	-1.17	-0.5	-3.21	-1.83	-1.49	-4.99	-2.41	-2.46	-5.03	-3.58	-2.88	-6.07
39	-2.07	-1.08	-3.42	-3.01	-2.5	-4.86	-3.48	-2.63	-5.31	-4.24	-3.77	-5.65
40	-0.7	-1.43	-2.19	-1.45	-2.49	-3.57	-1.9	-3.01	-4.03	-3.01	-3.82	-4.01
41	-0.65	-1.58	-2.21	-1.49	-2.63	-3.79	-1.9	-3.23	-4.03	-2.93	-4.04	-4.55
42	-0.61	-0.51	-2.44	-1.32	-1.17	-4.72	-1.72	-2.07	-4.28	-2.92	-3.03	-5.18
43	-0.6	-1.41	-1.16	-1.61	-2.71	-2.46	-2.03	-3.29	-2.94	-3.39	-3.68	-3.29
44	-0.04	-1.13	-1.17	-0.93	-2.56	-2.48	-1.24	-2.76	-3.04	-2.32	-3.31	-4.13
45	-0.43	-1.32	-1.36	-0.94	-3.32	-2.95	-1.37	-3.72	-3.41	-2.97	-3.75	-4.12
46	-1.12	-1.32	-1.17	-2.08	-2.63	-2.47	-2.51	-3.03	-2.94	-3.62	-3.56	-3.06
47	-0.77	-1.4	-1.23	-1.82	-2.34	-2.46	-2.06	-2.92	-2.95	-2.88	-3.89	-3.29
48	-0.68	-0.32	0.04	-1.5	-1.67	-1.18	-1.87	-1.95	-1.84	-2.99	-2.75	-2.81
49	-0.26	-2.33	-0.48	-0.85	-3.69	-3.73	-1.34	-4.04	-2.34	-2.69	-4.72	-3.17
50	-0.67	-1.18	-2.24	-1.5	-2.56	-3.62	-1.76	-2.81	-4.12	-2.84	-3.62	-4.23
51	-0.65	-2.05	-0.1	-1.24	-4.28	-3.32	-1.84	-3.93	-2.84	-3.14	-4.7	-3.86

52	-1.33	-1.19	-0.53	-1.99	-2.53	-1.91	-2.56	-3.05	-2.45	-3.5	-3.74	-3.15
53	-1.08	-1.55	-0.81	-2.09	-2.8	-2.2	-2.44	-3.11	-2.62	-3.46	-4.05	-2.98
54	-0.39	-1.7	-0.56	-1.49	-2.69	-2.19	-1.64	-3.32	-2.28	-2.61	-4	-3
55	-0.68	-0.98	-1.52	-1.26	-2.51	-2.81	-1.83	-2.63	-3.99	-2.99	-3.66	-4.05
56	-1.29	-0.53	-0.74	-2.31	-1.7	-2.13	-2.54	-2.28	-2.64	-3.62	-3.43	-2.67
57	0.43	-0.83	-0.5	-0.31	-2.02	-2.06	-0.68	-2.65	-2.47	-2.14	-3.66	-2.74
58	-0.6	-1.12	-0.83	-1.61	-2.43	-2.74	-1.92	-2.86	-3.72	-2.67	-3.93	-2.93
59	-0.03	-1.46	-0.16	-0.9	-2.4	-1.34	-1.29	-2.91	-2.16	-2.25	-3.75	-2.86
60	-0.19	-1.26	-1.25	-1.38	-2.85	-2.64	-1.83	-3.17	-3.19	-3.07	-3.65	-3.62
61	-0.6	-0.8	-1.91	-1.74	-1.42	-3.19	-2.09	-2.09	-3.57	-3.17	-3.25	-4
62	-0.51	-2.53	-1.24	-1.5	-3.91	-2.82	-1.82	-4.29	-3.19	-2.95	-5.19	-3.23
63	-0.39	-1.61	-1.33	-1.24	-2.85	-3.11	-1.58	-3.32	-3.43	-2.7	-4.17	-4.09
64	-0.58	-1.65	-1.24	-1.54	-4.02	-2.99	-1.75	-3.37	-3.04	-2.79	-3.95	-3.71
65	-0.79	-0.6	-1.26	-1.89	-1.79	-2.71	-2.13	-2.24	-3.03	-3.16	-3.2	-3.48
66	-0.41	-0.22	-1.02	-1.6	-1.19	-2.74	-1.81	-1.89	-2.95	-2.56	-3.08	-3.59
67	-1.31	-0.73	-0.26	-2.23	-1.74	-1.85	-2.63	-2.24	-2.21	-3.65	-3.28	-2.74
68	-0.73	-0.72	-2.18	-1.59	-2.17	-4.63	-1.8	-2.78	-4.23	-3.19	-3.84	-4.42
69	-0.76	-1.75	-1.38	-1.43	-2.69	-2.65	-1.96	-3.25	-3.11	-3.19	-4.01	-3.59
70	-0.65	-1.24	-1.88	-1.44	-4.05	-2.9	-1.65	-4.46	-3.55	-2.74	-3.88	-4.21
71	-0.16	-2.43	-1.93	-0.34	-3.77	-3.43	-1.44	-4.18	-3.73	-2.74	-4.78	-3.65
72	-0.07	-0.8	-2.84	-0.96	-1.85	-4.51	-1.31	-2.4	-4.76	-2.92	-3.46	-4.38
73	0.03	-1.55	-2.23	-0.92	-2.4	-4.02	-1.31	-3.05	-4.14	-2.36	-4.33	-4.41
74	-0.32	-0.52	-2.28	-1.74	-0.95	-4.01	-2.07	-2.09	-4.45	-2.69	-3.42	-4.43
75	-0.12	-1.45	-0.49	-0.95	-2.62	-1.72	-1.2	-3.22	-2.26	-2.25	-4.06	-3.17
76	-0.64	-1.44	-1.12	-1.24	-2.79	-2.47	-3.23	-3.11	-3.13	-3.03	-3.89	-3.67
77	-0.5	-1.31	-1.61	-1.22	-2.56	-2.54	-1.58	-3.09	-3.15	-2.72	-3.92	-3.65
78	-0.97	-1.39	-2.64	-1.7	-2.57	-4.08	-1.98	-3.09	-4.5	-3.22	-3.92	-4.34
79	-0.05	-1.21	-2.43	-0.73	-2.27	-3.86	-1.13	-2.82	-4.31	-2.46	-3.86	-4.33
80	-0.6	-2.25	-2.08	-1.22	-3.23	-3.66	-1.64	-3.84	-4.06	-2.94	-4.8	-4.41
81	-0.96	-1.07	-1.32	-1.56	-2.12	-2.62	-1.9	-2.93	-3.14	-3.06	-3.84	-3.41
82	-0.7	-2.12	-0.39	-1.41	-3.13	-3.16	-1.82	-3.77	-2.16	-3.16	-4.19	-4.5
83	-0.51	-1.58	0.5	-1.16	-2.7	-1.54	-1.8	-3.22	-1.48	-2.9	-3.95	-1.77
84	-0.53	-0.52	0.27	-1.17	-2.25	-1.58	-2.02	-2.68	-1.72	-2.5	-3.01	-1.27
85	-1.04	-1.53	-1.74	-1.4	-2.76	-4.02	-2	-3.18	-3.66	-3.18	-3.9	-3.95
86	-0.77	-0.7	-2.29	-1.58	-1.76	-4.58	-1.98	-2.35	-4.13	-3.06	-3.31	-4.53
87	-1.49	-0.85	-2.43	-2.89	-2.07	-4.11	-2.4	-2.56	-4.14	-4.18	-3.47	-4.07
88	-1.05	-1.48	-1.65	-1.98	-2.61	-3.46	-2.44	-3.16	-3.66	-3.48	-4.01	-4.77
89	-0.63	-0.91	-0.59	-3.05	-2	-3.67	-2.89	-2.76	-3.05	-3.62	-3.88	-3.01
90	-0.8	-0.8	-2.11	-1.55	-1.9	-3.78	-1.82	-2.49	-3.92	-3.21	-3.48	-4.4
91	-0.31	-1.66	-0.55	-0.97	-3.15	-2.4	-2.07	-3.58	-2.25	-2.51	-4.54	-3.12
92	-0.66	-1.54	-1.23	-1.39	-2.74	-2.47	-1.97	-3.29	-2.97	-3	-4.63	-3.58
93	0.41	-1.4	-1.07	-2.09	-2.74	-2.43	-1.33	-3.22	-2.88	-2.58	-3.92	-3.8
94	-0.79	-2.04	-1.28	-1.64	-3.24	-2.49	-1.95	-3.85	-3.08	-3.05	-4.76	-3.02
95	0.57	-0.61	-1.02	-0.2	-2.2	-2.46	-0.54	-2.42	-2.8	-1.77	-3.27	-2.82
96	0.87	-0.41	-1.36	-0.05	-4.48	-2.59	-0.31	-6.23	-3.19	-1.85	-4.57	-3.89
97	0.15	-1.42	-1.99	-0.79	-2.4	-3.62	-1.84	-2.96	-3.92	-2.94	-3.97	-4.32
98	-0.82	-0.58	-0.96	-1.59	-2.24	-1.82	-2.08	-2.64	-2.74	-3.12	-3.28	-3.53
99	-0.16	-0.92	-1.69	-1.19	-1.62	-2.99	-1.41	-2.35	-3.48	-2.61	-3.52	-3.76
100	-0.65	-1.35	-0.59	-1.57	-2.33	-2.25	-1.65	-2.84	-2.46	-3	-3.92	-3.4

Table S5. ΔG_{PDS} for eNORR to NH_3 on a hundred of top, bridge, and hollow sites

Site number	ΔG_{PDS} (eV)			Site number	ΔG_{PDS} (eV)		
	Top	Bridge	Hollow		Top	Bridge	Hollow
1	-0.35	0.47	0.41	51	-0.19	1.37	0.53
2	-0.05	1.13	-0.27	52	0.17	0.41	-0.18
3	-0.16	0.23	0.62	53	0.13	0.72	-0.35
4	-0.07	0.17	0.05	54	-0.15	0.67	-0.09
5	-0.4	0.95	1.7	55	-0.34	0.33	0.72
6	-0.23	0.82	0.88	56	0.29	0.1	-0.03
7	0.04	-0.25	1.23	57	0.43	0.33	-0.27
8	-0.25	0.16	0.66	58	-0.31	0.6	0.79
9	-0.1	0.79	1.8	59	-0.03	0.42	-0.16
10	-0.34	0.41	0.53	60	-0.19	0.32	0.29
11	-0.23	-0.13	0.17	61	-0.16	-0.08	0.67
12	-0.09	-0.25	-0.07	62	-0.32	1.86	-0.04
13	-0.09	-0.17	0.04	63	-0.34	0.84	0.76
14	0.31	-0.36	0.28	64	-0.21	0.65	0.38
15	0	-0.36	0.12	65	-0.17	-0.13	0.15
16	-0.34	1.01	-0.01	66	-0.21	-0.22	0.26
17	-0.33	0.61	0.35	67	0.32	-0.05	-0.26
18	-0.48	-0.06	-0.15	68	-0.14	0.51	1.09
19	0.37	0.67	-0.05	69	-0.14	0.68	0.26
20	-0.13	0.7	0.11	70	-0.21	0.58	0.88
21	-0.05	1.17	1.7	71	-0.16	1.45	0.32
22	-0.15	0.7	-0.24	72	-0.07	0.13	1.05
23	-0.1	0.9	0.83	73	0.03	1	1.08
24	-0.35	0.26	0.34	74	-0.32	0.09	1.1
25	-0.22	0.36	2.39	75	-0.12	0.73	-0.16
26	-0.44	0.86	0.6	76	0.2	0.56	0.34
27	-0.31	0.46	0.7	77	-0.36	0.59	0.32
28	-0.27	0.82	0.45	78	-0.11	0.59	1.01
29	-0.08	0.77	0.15	79	-0.05	0.53	1
30	-0.37	1.76	0.69	80	-0.39	1.47	1.08
31	-0.22	0.71	0.72	81	-0.27	0.51	0.08
32	-0.05	0.44	0.84	82	-0.17	0.86	1.17
33	0.01	0.27	0.83	83	-0.43	0.62	0.5
34	-0.16	-0.13	0.86	84	-0.48	-0.32	0.45
35	-0.2	0.36	1.08	85	-0.15	0.57	0.62
36	-0.05	0.23	0.31	86	-0.27	-0.02	1.2
37	-0.08	0.17	2.32	87	0.85	0.14	0.74
38	0.25	-0.42	2.74	88	0.15	0.68	1.44
39	0.91	0.44	2.32	89	0.29	0.55	0.62
40	-0.32	0.49	0.68	90	-0.12	0.15	1.07
41	-0.4	0.71	1.22	91	-0.31	1.21	0.15
42	-0.4	-0.3	1.85	92	-0.33	1.3	0.25
43	0.06	0.35	-0.04	93	0.76	0.59	0.47
44	-0.04	-0.02	0.8	94	-0.28	1.43	0.06
45	-0.36	0.42	0.79	95	0.57	-0.06	-0.02
46	0.29	0.23	-0.12	96	0.87	1.66	0.56
47	-0.24	0.56	-0.04	97	0.15	0.64	0.99
48	-0.34	-0.28	0.04	98	-0.21	-0.05	0.2
49	-0.26	1.39	1.39	99	-0.16	0.19	0.43
50	-0.26	0.29	0.9	100	-0.08	0.59	0.07

Table S6. Energy barriers of different N-N coupling reactions on pristine Fe and FeN_x motifs

Reactions	Energy barriers[#] (eV)	
	Pristine Fe	FeN_x motifs
$2N^* \rightarrow N_2^* + ^*$	2.32	0.17
$NO^* + NOH^* \rightarrow N_2O_2H^* + ^*$	2.02	0.43
$N^* + NO^* \rightarrow N_2O^* + ^*$	2.95	0.57
$2NOH^* \rightarrow N_2O_2H_2^* + ^*$	2.10	0.36
$N^* + NOH^* \rightarrow N_2OH^* + ^*$	2.15	0.12
$N^* + NO_g \rightarrow N_2O^*$	0.67	0.00

[#]These barriers were calculated using the vacuum surface.

Table S7. N-N coupling barriers calculated by EFC-CP method and vacuum surface

Reactions	EFC-CP					Vacuum surface
	-0.8 V	-0.5 V	-0.2 V	0.1 V	0.4V	
$N^*+NO_g \rightarrow N_2O^*$	0	0	0	0	0	0
$N^*+NOH^* \rightarrow N_2OH^*+^*$	0.12	0.11	0.10	0.12	0.12	0.12

Table S8. Considered elementary steps and calculated barriers and reaction free energies for eNORR to NH_3, N_2, N_2O, and HER for microkinetic modelling on FeN_x@top at different potentials

Elementary steps	Barrier (eV)					Reaction free energy (eV)				
	-0.8 V	-0.5 V	-0.2 V	0.1 V	0.4 V	-0.8 V	-0.5 V	-0.2 V	0.1 V	0.4 V
$NO_g + (H^+ + e^-) + * \rightarrow NOH*$	0	0	0	0	0	-1.15	-0.85	-0.55	-0.25	0.05
$NOH* + (H^+ + e^-) \rightarrow N* + H_2O$	0.39	0.45	0.5	0.57	0.6	-1.58	-1.28	-0.98	-0.68	-0.38
$N* + (H^+ + e^-) \rightarrow NH*$	0.04	0.001	0.08	0.26	0.33	-1.05	-0.75	-0.45	-0.15	0.15
$NH* + (H^+ + e^-) \rightarrow NH_2*$	0	0	0	0	0	-1.99	-1.69	-1.39	-1.09	-0.79
$NH_2* + (H^+ + e^-) \rightarrow NH_3 + *$	0	0	0.12	0.2	0.33	-1.56	-1.26	-0.96	-0.66	-0.36
$N* + NO_g \rightarrow N_2O*$	0	0	0	0	0	-2.21	-2.21	-2.21	-2.21	-2.21
$N_2O* \rightarrow N_2O + *$	0	0	0	0	0	-0.39	-0.39	-0.39	-0.39	-0.39
$N_2O* + (H^+ + e^-) \rightarrow N_2 + *OH$	0.2	0.2	0.23	0.23	0.23	-3.33	-3.03	-2.73	-2.43	-2.13
$OH* + (H^+ + e^-) \rightarrow H_2O + *$	0	0	0	0.23	0.5	-1.32	-1.02	-0.72	-0.42	-0.12
$2N* \rightarrow N_2 + 2*$	0.17	0.17	0.17	0.17	0.17	-4.13	-4.13	-4.13	-4.13	-4.13
$N* + NOH* \rightarrow N_2OH* + *$	0.12	0.12	0.12	0.12	0.12	-1.71	-1.71	-1.71	-1.71	-1.71
$N_2OH* + (H^+ + e^-) \rightarrow N_2 + H_2O + *$	0	0	0	0	0	-3.5	-3.2	-2.9	-2.6	-2.3
$(H^+ + e^-) + * \rightarrow H*$	0.38	0.58	0.67	0.8	0.93	-0.57	-0.27	0.03	0.33	0.63
$H* + (H^+ + e^-) \rightarrow H_2 + *$	0.13	0.27	0.37	0.44	0.51	-1.03	-0.73	-0.43	-0.13	0.17

Note that the barriers under other potentials in microkinetic modelling were obtained by linear interpolation method.

Table S9. Solvation energies of different adsorbed species on Fe (110) surface

Species	Solvation energy (eV)
N*	-0.02
HNO*	-0.11
H_2NO*	-0.27
HNOH*	-0.14
NH*	-0.05
NH_2*	-0.12
H_2NOH*	-0.24
NOH*	-0.16

Table S10. Coverages of intermediates (θ) for eNORR to ammonia on Co, Cu, and Ag

**Catalysts**	$\boldsymbol{\theta_{*}}$	$\boldsymbol{\theta_{NOH*}}$	$\boldsymbol{\theta_{N*}}$	$\boldsymbol{\theta_{NH*}}$	$\boldsymbol{\theta_{NH_2*}}$
Co (111)	1.50×10^{-9}	1.50×10^{-11}	7.63×10^{-8}	0.999	1.50×10^{-11}
Cu (111)	7.64×10^{-4}	0.771	0.016	0.162	0.050
Ag (111)	0.999	3.38×10^{-13}	4.43×10^{-28}	3.44×10^{-24}	2.07×10^{-15}

References

1. Kresse G, Furthmuller J Efficient iterative schemes for ab initio total-energy calculations using a plane-wave basis set. *Phys. Rev. B* 1996; **54**: 11169-11186.

2. Kresse G, Furthmuller J Efficiency of ab-initio total energy calculations for metals and semiconductors using a plane-wave basis set. *Comput. Mater. Sci.* 1996; **6**: 15-50.

3. Zhang Y K, Yang W T Comment on "Generalized gradient approximation made simple". *Phys. Rev. Lett.* 1998; **80**: 890-890.

4. Grimme S Semiempirical GGA-type density functional constructed with a long-range dispersion correction. *Journal of Computational Chemistry* 2006; **27**: 1787-1799.

5. Mathew K, Sundararaman R, Letchworth-Weaver K et al. Implicit solvation model for density-functional study of nanocrystal surfaces and reaction pathways. *J Chem Phys* 2014; **140**: 084106.

6. Long J, Chen S, Zhang Y et al. Direct Electrochemical Ammonia Synthesis from Nitric Oxide. *Angew Chem Int Ed Engl* 2020; **59**: 9711-9718.

7. Henkelman G, Jónsson H Improved tangent estimate in the nudged elastic band method for finding minimum energy paths and saddle points. *Journal of Chemical Physics* 2000; **113**: 9978-9985.

8. Henkelman G, Uberuaga B P, JóNsson H A Climbing Image Nudged Elastic Band Method for Finding Saddle Points and Minimum Energy Paths. *Journal of Chemical Physics* 2000; **113**: 9901-9904.

9. Fu X, Li J, Long J et al. Understanding the Product Selectivity of Syngas Conversion on ZnO Surfaces with Complex Reaction Network and Structural Evolution. *ACS Catalysis* 2021; **11**: 12264-12273.

10. Shao J, Jing H, Wei P et al. Electrochemical synthesis of ammonia from nitric oxide using a copper–tin alloy catalyst. *Nature Energy* 2023; **8**: 1273-1283.

11. Li H, Long J, Jing H et al. Steering from electrochemical denitrification to ammonia synthesis. *Nature communications* 2023; **14**.

12. Guo C, Fu X, Long J et al. Toward computational design of chemical reactions with reaction phase diagram. *WIREs Computational Molecular Science* 2021; **11**.

13. Luan D, Xiao J Adaptive Electric Fields Embedded Electrochemical Barrier Calculations. *The journal of physical chemistry letters* 2023; **14**: 685-693.

14. Long J, Luan D, Fu X et al. Theoretical Design of the Electrocatalytic Urea Synthesis from Carbon Dioxide and Nitric Oxides. *ACS Catalysis* 2024; 14678-14687.

15. Van Soestbergen M Frumkin-Butler-Volmer theory and mass transfer in electrochemical cells. *Russian journal*

of electrochemistry 2012; **48**: 570-579.

16. Zhang Y, Xia J, Jiang B REANN: A PyTorch-based end-to-end multi-functional deep neural network package for molecular, reactive, and periodic systems. *J Chem Phys* 2022; **156**: 114801.

17. Zhang Y, Xia J, Jiang B Physically Motivated Recursively Embedded Atom Neural Networks: Incorporating Local Completeness and Nonlocality. *Phys Rev Lett* 2021; **127**: 156002.

18. Zhang Y, Hu C, Jiang B Embedded Atom Neural Network Potentials: Efficient and Accurate Machine Learning with a Physically Inspired Representation. *The Journal of Physical Chemistry Letters* 2019; **10**: 4962-4967.

19. NØRSKOV, J. K, ROSSMEISL et al. Origin of the overpotential for oxygen reduction at a fuel-cell cathode. *Journal of Physical Chemistry B* 2004; **108**: 17886-17892.

20. Guo P, Luan D, Li H et al. Computational Insights on Structural Sensitivity of Cobalt in NO Electroreduction to Ammonia and Hydroxylamine. *Journal of the American Chemical Society* 2024; **146**: 13974-13982.

21. Long J, Chen S, Zhang Y et al. Direct Electrochemical Ammonia Synthesis from Nitric Oxide. *Angewandte Chemie International Edition* 2020; **59**: 9711-9718.

22. Long J, Guo C, Fu X et al. Unveiling Potential Dependence in NO Electroreduction to Ammonia. *J Phys Chem Lett* 2021; **12**: 6988-6995.

23. Kim D, Shin D, Heo J et al. Unveiling Electrode–Electrolyte Design-Based NO Reduction for NH3 Synthesis. *ACS Energy Letters* 2020; **5**: 3647-3656.

24. Cheon S, Kim W J, Kim D Y et al. Electro-synthesis of Ammonia from Dilute Nitric Oxide on a Gas Diffusion Electrode. *ACS Energy Letters* 2022; **7**: 958-965.

25. Wang D, Fan G, Luan D et al. Ru-Incorporation-Induced Phase Transition in Co Nanoparticles for Low-Concentration Nitric Oxide Electroreduction to Ammonia at Low Potential. *Advanced Materials* 2024; **36**.

26. Meng J, Cheng C, Wang Y et al. Carbon Support Enhanced Mass Transfer and Metal–Support Interaction Promoted Activation for Low-Concentrated Nitric Oxide Electroreduction to Ammonia. *Journal of the American Chemical Society* 2024; **146**: 10044-10051.

27. Lin Y, Liang J, Li H et al. Bi nanodendrites for highly efficient electrocatalytic NO reduction to NH3 at ambient conditions. *Materials Today Physics* 2022; **22**: 100611.

28. Bunea S, Coppens M, Urakawa A Electrochemical Conversion of NO to NH3 in a PEM Cell. *ACS Catalysis* 2023; **13**: 11345-11351.

29. Li Y, Cheng C, Han S et al. Electrocatalytic Reduction of Low-Concentration Nitric Oxide into Ammonia over

Ru Nanosheets. *ACS Energy Letters* 2022; **7**: 1187-1194.

30. Chen K, Zhang Y, Xiang J et al. p-Block Antimony Single-Atom Catalysts for Nitric Oxide Electroreduction to Ammonia. *ACS Energy Letters* 2023; **8**: 1281-1288.

31. Li Z, Ma Z, Liang J et al. MnO2 nanoarray with oxygen vacancies: An efficient catalyst for NO electroreduction to NH3 at ambient conditions. *Materials Today Physics* 2022; **22**: 100586.

32. Li Z, Zhou Q, Liang J et al. Defective TiO2−x for High-Performance Electrocatalytic NO Reduction toward Ambient NH3 Production. *Small* 2023; **19**.

33. Liu P, Liang J, Wang J et al. High-performance NH3 production via NO electroreduction over a NiO nanosheet array. *Chemical Communications* 2021; **57**: 13562-13565.

34. Mou T, Liang J, Ma Z et al. High-efficiency electrohydrogenation of nitric oxide to ammonia on a Ni2P nanoarray under ambient conditions. *Journal of Materials Chemistry A* 2021; **9**: 24268-24275.

35. Liang J, Zhou Q, Mou T et al. FeP nanorod array: A high-efficiency catalyst for electroreduction of NO to NH3 under ambient conditions. *Nano Research* 2022; **15**: 4008-4013.

36. Zhang L, Liang J, Wang Y et al. High-Performance Electrochemical NO Reduction into NH3 by MoS2 Nanosheet. *Angewandte Chemie International Edition* 2021; **60**: 25263-25268.

37. Zhang L, Zhou Q, Liang J et al. Enhancing Electrocatalytic NO Reduction to NH(3) by the CoS Nanosheet with Sulfur Vacancies. *Inorg Chem* 2022; **61**: 8096-8102.

38. Liang J, Liu P, Li Q et al. Amorphous Boron Carbide on Titanium Dioxide Nanobelt Arrays for High-Efficiency Electrocatalytic NO Reduction to NH3. *Angewandte Chemie International Edition* 2022; **61**.

39. Ouyang L, Zhou Q, Liang J et al. High-efficiency NO electroreduction to NH3 over honeycomb carbon nanofiber at ambient conditions. *Journal of Colloid and Interface Science* 2022; **616**: 261-267.
